# Chemistry and bioactivities of natural steroidal alkaloids

**DOI:** 10.1007/s13659-022-00345-0

**Published:** 2022-06-15

**Authors:** Mei-Ling Xiang, Bin-Yuan Hu, Zi-Heng Qi, Xiao-Na Wang, Tian-Zhen Xie, Zhao-Jie Wang, Dan-Yu Ma, Qi Zeng, Xiao-Dong Luo

**Affiliations:** 1 Key Laboratory of Medicinal Chemistry for Natural Resource, Ministry of Education, Yunnan Provincial Center for Research & Development of Natural Products, School of Chemical Science and Technology, Yunnan University, Kunming, 650091 People’s Republic of China; 2 State Key Laboratory of Phytochemistry and Plant Resources in West China, Kunming Institute of Botany, Chinese Academy of Sciences, Kunming, 650201 People’s Republic of China

**Keywords:** Steroidal alkaloids, Chemistry, Bioactivities, Solanaceae, Liliaceae, Apocynaceae, Buxaceae

## Abstract

Steroidal alkaloids possess the basic steroidal skeleton with a nitrogen atom in rings or side chains incorporated as an integral part of the molecule. They have demonstrated a wide range of biological activities, and some of them have even been developed as therapeutic drugs, such as abiraterone acetate (Zytiga^®^), a blockbuster drug, which has been used for the treatment of prostate cancer. Structurally diverse natural steroidal alkaloids present a wide spectrum of biological activities, which are attractive for natural product chemistry and medicinal chemistry communities. This review comprehensively covers the structural classification, isolation and various biological activities of 697 natural steroidal alkaloids discovered from 1926 to October 2021, with 363 references being cited.

## Introduction

Steroidal alkaloids are nitrogenous derivatives of natural steroids. They are an important class of alkaloids and conventional secondary metabolites that occur in plants including Solanaceae, Liliaceae, Apocynaceae, Buxaceae, amphibians and marine organisms. Previous research results exhibited that steroidal alkaloids possess potential anticancer, anticholinergic, antimicrobial, anti-inflammatory and analgesic, anti-myocardial ischemia, anti-giogenesis effects and other activities.

Steroidal alkaloids are already launched as drugs, such as abiraterone acetate, marketed as Zytiga^®^ by Janssen Biotech (a subsidiary of Johnson & Johnson), is a steroidal antiandrogen medication approved by the Food and Drug Administration (FDA) for the treatment of metastatic castration resistant prostate cancer (mCRPC) in 2011 and metastatic high-risk castration-sensitive prostate cancer (mCSPC) in 2018 [1]. Zytiga^®^ is a blockbuster drug on the prostate cancer market, and in 2020, it generated almost $2.4 billion in sales from the Johnson & Johnson annual report, with ongoing research into its application for additional indications. Natural steroidal alkaloid cyclovirobuxine D (**203**) is the main active component of oral drug “huangyangning” tablets listed in the Chinese pharmacopeia 2015. This drug, discovered from a folk prescription in the treatment of rheumatic disease, was approved by the China Food and Drug Administration (CFDA) in 2009 to treat cardiovascular and cerebrovascular diseases, such as coronary heart disease, angina pectoris, arrhythmia, heart failure, hypertension and cardiac neurosis [2]. Several steroidal glycoalkaloids from the local plant *Solanum linnaeanum* possess activity of slowing skin cancer growth in horses and cattle, in which *α*-solamargine (**500**) and *α*-solasonine (**501**) were identified. These two active compounds were subsequently developed into a topical treatment for keratoses, basal cell carcinomas, and squamous cell carcinomas, which were marketed in Australia signed Curaderm [3].

Sheep ranchers experienced outbreaks of cyclopic lambs, leading to the discovery of cyclopamine (**432**) as a plant derived teratogen [4]. Cyclopamine was the first compound found to antagonize the Hedgehog (Hh) signaling pathway, the constitutive activation of which is intimately implicated in many human malignancies [5]. Vismodegib and sonidegib, cyclopamine derivatives and Hh pathway inhibitors, were FDA-approved for the treatment of basal cell carcinoma and acute myeloid leukemia, respectively [6]. In 2016 cyclopamine was identified as a potent inhibitor of human respiratory syncytial virus (hRSV) replication [7].

*Phyllobates terribilis* frogs, made into poison darts by Central American indigenous people, advertise their lethal armament with their gaudy colors. Batrachotoxin (**615**) is a potent neurotoxin in the skin secretions of these frogs and as a tool to study voltage-sensitive sodium channels of excitable membranes [8, 9]. Toxicity is widespread among living organisms and how these frogs avoid poisoning themselves remains a mystery. A study addressed that a single rat muscle Na^+^ channel mutation confers batrachotoxin autoresistance [10].

Some reviews related to steroidal alkaloids have been presented since 1953. For example, the chemistry of these alkaloids from the Liliaceae and Solanaceae [11–15], the Apocynaceaethe [16], the Buxaceae [17, 18], the marine organisms [19], synthesis of cephalostatins and ritterazines [20], biosynthesis of Buxaceae alkaloids [21], and biological activities [22, 23]. A comprehensive review was published in 1998 concerning the developments in the field of steroidal alkaloids [24]. In consideration of these reviews providing little information about recent research, we provide an updated review in a concise form, covering comprehensive structure classification, resources, biosynthesis and bioactivities of natural steroid alkaloids reported from 1926 to October 2021.

This review will help the scientific community understand natural steroidal alkaloids overall and compactly. We comprehensively summarize 16 structural subtypes of steroidal alkaloids along with their bioactivities and toxicity. In addition, steroidal alkaloids (**362–365**, **381**) whose names were not proposed by authors were presented only with numbers in the tables.

## Basic skeletal classification

**Fig. 1:**
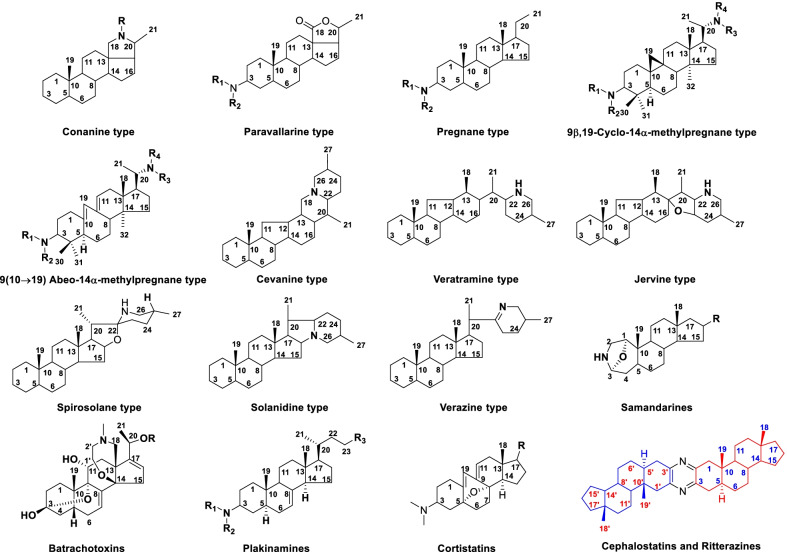
Classification of steroidal alkaloids

Steroidal alkaloids possess the basic steroidal skeleton with a nitrogen atom in rings or side chains incorporated as an integral part of the molecule [24]. In general, steroidal alkaloids can be classified into monomeric and dimeric on the basis of the carbon framework. Monomeric steroidal alkaloids, possessing a pregnane (C_21_), cyclopregnane (C_24_), cholestane (C_27_) and other carbon heterocyclic skeletons, were isolated from plants, amphibians and some marine sponges. Dimeric steroidal alkaloids, a class of bis-steroidal pyrazine alkaloids, were only found in marine organisms. Figure 1 lists the different types of natural steroidal alkaloids.

### Monomeric steroidal alkaloids

#### Pregnane alkaloids

The occurrence of 177 pregnane alkaloids (**1–177**), however, is not restricted to the Apocynaceae family, which is also found in Buxaceae, such as *Sarcococca* and *Pachysandra*.

##### Conanine type

**Fig. 2:**
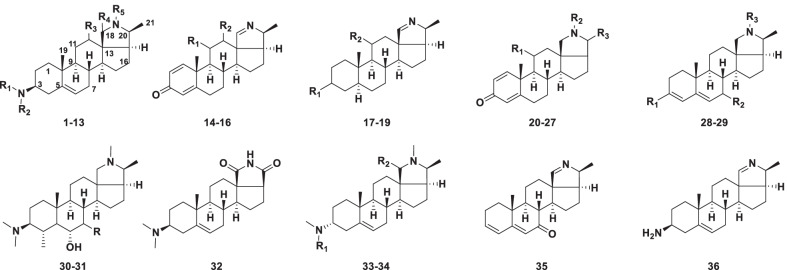
Structures of conanine type steroidal alkaloids **1–36**

Conanine type alkaloids are characteristic of an 18,20-epimino five-membered E ring, and most of them contain an amino or oxygen at C-3 (Fig. 2). Alkaloids (**1–36**) were isolated from various plants of the family Apocynaceae, such as *Holarrhena*, *Funtumia*, *Malouetia*, and *Wrightia* (Table 1).

**Table 1:** Structures and sources of conanine type steroidal alkaloids **1–36**

No	Compounds	Substitution groups and others	Sources	References
**1**	Conessine	R_1_ = R_2_ = R_5_ = CH_3_; R_3_ = R_4_ = H	*Holarrhena antidysenterica*	[25]
**2**	7*α*-Hydroxy-conessine	R_1_ = R_2_ = R_5_ = CH_3_; R_3_ = R_4_ = H; 7-*α*-OH	*H. antidysenterica*	[27]
**3**	Regholarrhenine D	R_1_ = R_2_ = CH_3_; R_3_ = R_4_ = H; R_5_ = OH	*H. antidysenterica*	[28]
**4**	Antidysentericine	R_1_ = R_2_ = CH_3_; R_3_ = R_5_ = H; R_4_ = O	*H. antidysenterica*	[29]
**5**	Isoconessimine	R_1_ = R_5_ = CH_3_; R_2_ = R_3_ = R_4_ = H	*Funtumia elastica*	[30]
**6**	Holarrhetine	R_1_ = R_2_ = R_5_ = CH_3_; R_3_ = *β*-(CH_3_)_2_C = CHCH_2_COO; R_4_ = H	*F. elastica*	[30]
**7**	Holarrhesine	R_1_ = R_4_ = H; R_2_ = R_5_ = CH_3_; R_3_ = *β*-(CH_3_)_2_C = CHCH_2_COO	*F. elastica*	[30]
**8**	Conessimin	R_1_ = R_2_ = CH_3_; R_3_ = R_4_ = R_5_ = H	*Holarrhena antidysenterica*	[31]
**9**	Conarrhimin	R_1_ = R_2_ = R_3_ = R_4_ = R_5_ = H	*H. antidysenterica*	[31]
**10**	Conimin	R_1_ = CH_3_; R_2_ = R_3_ = R_4_ = R_5_ = H	*H. antidysenterica*	[31]
**11**	Mokluangin A	R_1_ = R_3_ = R_5_ = H; R_2_ = CH_3_; R_4_ = O	*H. pubescens*	[26]
**12**	Mokluangin C	R_1_ = R_2_ = R_3_ = R_5_ = H; R_4_ = O	*H. pubescens*	[26]
**13**	*N*-Formylconessimine	R_1_ = R_2_ = CH_3_; R_3_ = R_4_ = H; R_5_ = CHO	*H. antidysenterica*	[32]
**14**	Holonamine	R_1_ = *α*-OH; R_2_ = H	*H. antidysenterica*	[27]
**15**	12*α*-Hydroxynorcona-*N*(18),1,4-trienin-3-one	R_1_ = H; R_2_ = *α*-OH	*Funtumia africana*	[33]
**16**	11*α*,l2*α*-Dihydroxynorcona-*N*(18),1,4-trienin-3-one	R_1_ = R_2_ = *α*-OH	*F. africana*	[33]
**17**	Conkurchine	R_1_ = *β*-NH_2_; R_2_ = H; △^5,6^	*Holarrhena antidysenterica*	[34]
**18**	Malouetafrine	R_1_ = O; R_2_ = H; △^4,5^	*Malouetia brachyloba*	[35]
**19**	Wrightiamine A	R_1_ = *β*-NH_2_; R_2_ = *α*-OAc	*Wrightia javanica*	[36]
**20**	Regholarrhenine A	R_1_ = *α*-OH; R_2_ = CH_3_; R_3_ = *β*-CH_3_	*Holarrhena antidysenterica*	[37]
**21**	Regholarrhenine B	R_1_ = *α*-OH; R_2_ = H; R_3_ = *β*-CH_3_	*H. antidysenterica*	[37]
**22**	Holadiene	R_1_ = H; R_2_ = R_3_ = CH_3_	*H. pubescens*	[38]
**23**	Kurchinidine	R_1_ = R_2_ = H; R_3_ = CH_3_	*H. pubescens*	[38]
**24**	Kurchilidine (I)	R_1_ = R_2_ = H; R_3_ = *β*-Et	*H. antidysenterica*	[39]
**25**	Kuchamide (II)	R_1_ = OH; R_2_ = H; R_3_ = O	*H. antidysenterica*	[39]
**26**	Holamide	R_1_ = H; R_2_ = CONHCH_3_; R_3_ = CH_3_	*H. antidysenterica*	[40]
**27**	Pubescinine	R_1_ = *α*-OAc; R_2_ = H; R_3_ = CH_3_; △^17,20^	*H. antidysenterica*	[40]
**28**	Regholarrhenine C	R_1_ = NHCH_3_; R_2_ = R_3_ = H	*H. antidysenterica*	[37]
**29**	Funtudienine	R_1_ = H; R_2_ = O; R_3_ = CH_3_	*H. antidysenterica*	[32]
**30**	Kurcholessine	R = *β*-OH	*H. antidysenterica*	[28]
**31**	Regholarrhenine E	R = *α*-OH	*H. antidysenterica*	[28]
**32**	Mokluangin B		*H. pubescens*	[26]
**33**	Isoconkuressine	R_1_ = R_2_ = H	*H. antidysenterica*	[32]
**34**	Conkuressine	R_1_ = CH_3_; R_2_ = H	*H. antidysenterica*	[32]
**35**	Mokluangin D		*H. pubescens*	[41]
**36**	Irehline		*H. pubescens*	[41]

Conessine (**1)** was the first and most common conanine type alkaloid isolated from the seeds of *Holarrhena antidysenterica* [25]. Rings A and B of the pregnane moiety were dehydrogenated to form a conjugated system comprising two double bonds in regholarrhenine C (**28**), funtudienine (**29**), mokluangin D (**35**). Compounds **14–16** and **20–27** have secondary and tertiary amino group of the nitrogen in the heterocyclic ring, respectively, but both of them lack the C-3 amino function and possess a 1,4-dien-3-one system in ring A. Mokluangin B (**32**) contains a novel structure with the amide carbonyl group instead of the methyl group at C-20, whose structure was elucidated by analysis of NMR and MS spectroscopic data [26].

##### Paravallarine type

**Fig. 3:**
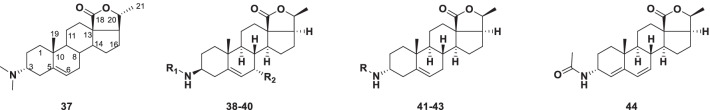
Structures of paravallarine type steroidal alkaloids **37–44**

Paravallarine type alkaloids bear a pregnane-(18 → 20)-lactone skeleton (Fig. 3) [42]. Currently, eight compounds (**37–44**) of this type have been found only in Apocynaceae family, including *Kibatalia* and *Paravallaris* (Table 2).

**Table 2:** Structures and sources of paravallarine type steroidal alkaloids **37–44**

No	Compounds	Substitution groups and others	Sources	References
**37**	20-*Epi*-kibataline		*Paravallaris macrophylla*	[43]
**38**	3-*Epi*-gitingensine	R_1_ = R_2_ = H	*Kibatalia laurifolia*	[42]
**39**	Paravallarine	R_1_ = CH_3_; R_2_ = H	*K. laurifolia*	[42]
**40**	7*α*-Hydroxyparavallarine	R_1_ = CH_3_; R_2_ = OH	*K. laurifolia*	[42]
**41**	Gitingensine	R = H	*K. laurifolia*	[42]
**42**	*N*-Methylgitingensine	R = CH_3_	*K. laurifolia*	[42]
**43**	*N*-Acetylgitingensine	R = Ac	*K. laurifolia*	[42]
**44**	Kibalaurifoline		*K. laurifolia*	[42]

The structure of 20-*epi*-kibataline (**37**) contains a rare configuration 20*R*, while the configuration 20*S* is proposed for all remaining compounds [43]. Compounds **38–40** differ from others possessing an opposite orientation at C-3. The structure of kibalaurifoline (**44**) was carefully established from 2D NMR analyses, in which the conjugated system of the two double bonds Δ^4(5)^ and Δ^6(7)^ was determined from the HMBC spectrum [42].

##### Pregnane type

**Fig. 4:**
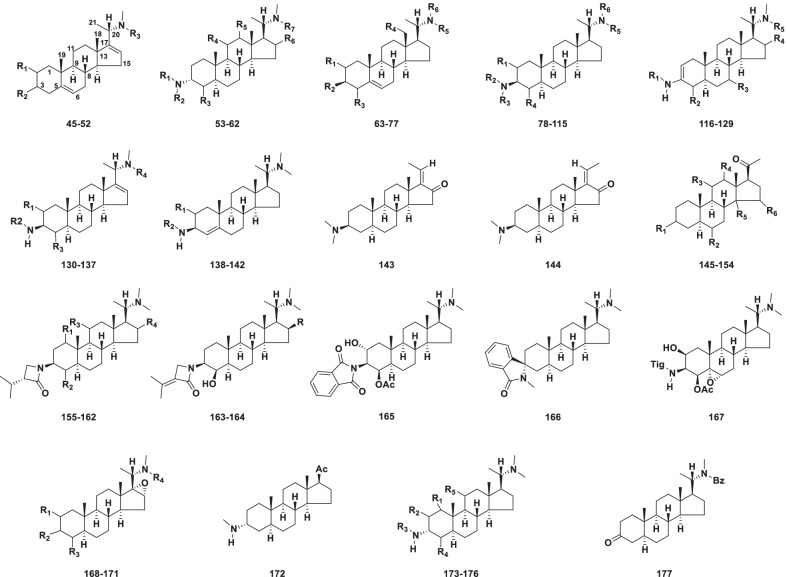
Structures of pregnane type steroidal alkaloids **45–177**

Nearly all the reported pregnane type alkaloids, share the 5*α*-pregnane steroidal skeleton with varying functionalities such as an amino function at C-3 and C-20 that may be modified by methyl, benzoyl and aliphatic groups (Fig. 4). A total of 133 new alkaloids (**45–177)** were isolated from *Sarcococca* and *Pachysandra* of the Buxaceae family and *Holarrhena* of the Apocynaceae family (Table 3).

**Table 3:** Structures and sources of pregnane type steroidal alkaloids **45–177**

No	Compounds	Substitution groups and others	Sources	References
**45**	Saracocinaene	R_1_ = H; R_2_ = *α*-N(CH_3_)_2_; R_3_ = Ac	*Sarcococca saligna*	[48]
**46**	Sarconidine	R_1_ = H; R_2_ = *β*-NHCH_3_; R_3_ = CH_3_	*S. saligna*	[49]
**47**	Salonine B	R_1_ = H; R_2_ = *β*-OCH_3_; R_3_ = CH_3_	*S. saligna*	[50]
**48**	Salignamine	R_1_ = R_3_ = H; R_2_ = *β*-OCH_3_	*S. saligna*	[51]
**49**	2-Hydroxysalignamine	R_1_ = *β*-OH; R_2_ = *β*-OCH_3_; R_3_ = CH_3_	*S. saligna*	[51]
**50**	*N*-[Formyl(methyl)amino]salonine B	R_1_ = H; R_2_ = *β*-OCH_3_; R_3_ = CHO	*S. saligna*	[51]
**51**	Wallichimine A	R_1_ = H; R_2_ = *β*-N(CH_3_)_2_; R_3_ = CH_3_	*S. wallichii*	[52]
**52**	Wallichimine B	R_1_ = R_3_ = H; R_2_ = *β*-N(CH_3_)_2_	*S. wallichii*	[52]
**53**	Sarcodine	R_1_ = R_2_ = CH_3_; R_3_ = R_4_ = R_5_ = R_6_ = H; R_7_ = Ac	*S. saligna*	[48]
**54**	Paxillarine A	R_1_ = Bz; R_2_ = R_7_ = CH_3_; R_3_ = *β*-OAc; R_4_ = R_5_ = H; R_6_ = *β*-OH	*Pachysandra axillaris*	[53]
**55**	Paxillarine B	R_1_ = Bz; R_2_ = R_7_ = CH_3_; R_3_ = *β*-OAc; R_4_ = R_6_ = H; R_5_ = *β*-OH	*P. axillaris*	[53]
**56**	Pachysamine B	R_1_ = Sen; R_2_ = R_7_ = CH_3_; R_3_ = R_4_ = R_5_ = R_6_ = H	*P. procumbens*	[46]
**57**	Pachysamine E	R_1_ = Sen; R_2_ = R_3_ = R_4_ = R_5_ = R_6_ = H; R_7_ = CH_3_	*P. terminalis*	[54]
**58**	(+)-(20*S*)-20-(Dimethylamino)-3*α*-(methylbenzoylamino)-11-methylene-5*α*-pregnane	R_1_ = Bz; R_2_ = R_3_ = R_5_ = R_6_ = H; R_4_ = CH_2_; R_7_ = CH_3_	*P. procumbens*	[55]
**59**	(+)-(20*S*)-20-(Dimethylamino)-3*α*-(methylbenzoylamino)-5*α*-pregn-12*β*-yl acetate	R_1_ = Bz; R_2_ = R_3_ = R_4_ = R_6_ = H; R_5_ = *β*-OAc; R_7_ = CH_3_	*P. procumbens*	[55]
**60**	(+)-(20*S*)-20-(Dimethylamino)-3*α*-(methylsenecioylamino)-5*α*-pregn-12*β*-ol	R_1_ = Sen; R_2_ = R_3_ = R_4_ = R_6_ = H; R_5_ = *β*-OH; R_7_ = CH_3_	*P. procumbens*	[55]
**61**	Hookerianine A	R_1_ = CO-Bn; R_2_ = R_3_ = R_4_ = R_5_ = R_6_ = H; R_7_ = CH_3_	*Sarcococca hookeriana*	[56]
**62**	Sarchookloide C	R_1_ = Tig; R_2_ = R_3_ = R_4_ = R_5_ = R_6_ = H; R_7_ = CH_3_	*S. hookeriana*	[57]
**63**	Pachyaximine A	R_1_ = R_3_ = R_4_ = H; R_2_ = OCH_3_; R_5_ = R_6_ = CH_3_	*S. saligna*; *P. procumbens*	[46, 48]
**64**	Sarsalignone	R_1_ = R_4_ = H; R_2_ = NH-Tig; R_3_ = O; R_5_ = R_6_ = CH_3_	*S. saligna*	[58]
**65**	Sarsaligenone	R_1_ = R_4_ = H; R_2_ = NH-Tig; R_3_ = O; R_5_ = R_6_ = CH_3_; △^14,15^	*S. saligna*	[58]
**66**	Epipachysamine-*E*-5-en-4-one	R_1_ = R_4_ = H; R_2_ = NH-Sen; R_3_ = O; R_5_ = R_6_ = CH_3_	*S.brevifolia*	[59]
**67**	*N*_b_-Demethylepipachysamine-*E*-5-ene-4-one	R_1_ = R_4_ = R_5_ = H; R_2_ = NH-Sen; R_3_ = O; R_6_ = CH_3_	*S. brevifolia*	[59]
**68**	Salignarine B	R_1_ = *β*-OH; R_2_ = NH-Tig; R_3_ = R_4_ = H; R_5_ = R_6_ = CH_3_	*S. saligna*	[44]
**69**	Salignarine C	R_1_ = *β*-OH; R_2_ = NH-Sen; R_3_ = R_4_ = H; R_5_ = R_6_ = CH_3_	*S. saligna*	[44]
**70**	Iso-*N*-formylchonemorphin-5-ene	R_1_ = R_3_ = R_4_ = R_5_ = H; R_2_ = N(CH_3_)_2_; R_6_ = CHO	*S. zeylanica*	[60]
**71**	Alkaloid C	R_1_ = R_3_ = R_4_ = H; R_2_ = OCH_3_; R_5_ = R_6_ = CH_3_	*S. saligna*	[50]
**72**	Salignarine F	R_1_ = R_4_ = H; R_2_ = NH-Tig; R_3_ = *β*-OH; R_5_ = R_6_ = CH_3_	*S. saligna*	[51]
**73**	Saracosine	R_1_ = R_3_ = R_4_ = H; R_2_ = N(CH_3_)_2_; R_5_ = Ac; R_6_ = CHO	*S. saligna*	[51]
**74**	Sarcodinine	R_1_ = R_3_ = R_4_ = H; R_2_ = N(CH_3_)_2_; R_5_ = R_6_ = CH_3_	*S. saligna*	[51]
**75**	5,14-Dehydro-*N*_a_-demethylsaracodine	R_1_ = R_3_ = R_4_ = H; R_2_ = NHCH_3_; R_5_ = Ac; R_6_ = CH_3_; △^14,15^	*S. saligna*	[61]
**76**	Holadysenterine	R_1_ = R_3_ = H; R_2_ = NH_2_; R_4_ = R_5_ = OH; R_6_ = Ac	*Holarrhena antidysenterica*	[62]
**77**	(20*S*)-20*α*-Cinnamoylamino-3*β*-dimethylamino-5-en-pregnane	R_1_ = R_3_ = R_4_ = R_5_ = H; R_2_ = N(CH_3_)_2_; R_6_ = COCH = CHph	*Pachysandra terminalis*	[63]
**78**	SarcovagineA	R_1_ = R_4_ = *β*-OH; R_2_ = Tig; R_3_ = H; R_5_ = R_6_ = CH_3_	*Sarcococca vegans*	[64]
**79**	Sarcovagine B	R_1_ = *α*-OH; R_2_ = Tig; R_3_ = H; R_4_ = *β*-OAc; R_5_ = R_6_ = CH_3_	*S. vegans*	[64]
**80**	Sarcovagine C	R_1_ = R_3_ = H; R_2_ = Tig; R_4_ = *β*-OAc; R_5_ = R_6_ = CH_3_	*S. vegans*; *S. hookeriana*	[64, 65]
**81**	*N*-Formylchonemorphine	R_1_ = R_2_ = R_4_ = H; R_3_ = CHO; R_5_ = R_6_ = CH_3_	*S. saligna*	[58]
**82**	Vaganine A	R_1_ = R_2_ = H; R_3_ = Sen; R_4_ = *β*-OAc; R_5_ = R_6_ = CH_3_	*S. saligna*	[58]
**83**	Sarcorine	R_1_ = R_2_ = R_4_ = H; R_3_ = Ac; R_5_ = R_6_ = CH_3_	*S. saligna*	[49]
**84**	*N*_a_-Demethylsaracodine	R_1_ = R_2_ = R_4_ = H; R_3_ = R_6_ = CH_3_; R_5_ = Ac	*S. saligna*	[66]
**85**	Saligcinnamide	R_1_ = R_4_ = H; R_2_ = R_5_ = R_6_ = CH_3_; R_3_ = Cin	*S. saligna*	[67]
**86**	*N*_a_-Methyl epipachysamine D	R_1_ = R_4_ = H; R_2_ = R_5_ = R_6_ = CH_3_; R_3_ = Bz	*S. saligna*; *S. hookeriana*	[65, 67]
**87**	Epipachysamine D	R_1_ = R_2_ = R_4_ = H; R_3_ = Bz; R_5_ = R_6_ = CH_3_	*S. saligna*	[67]
**88**	Salignenamide A	R_1_ = R_2_ = R_4_ = H; R_3_ = COCHC(CH_3_)CH(CH_3_)CH_3_; R_5_ = R_6_ = CH_3_	*S. saligna*; *S. hookeriana*	[65, 68]
**89**	2,4-Diacetoxyepipachysamine D	R_1_ = *β*-OAc; R_2_ = H; R_3_ = Bz; R_4_ = *β*-OAc; R_5_ = R_6_ = CH_3_	*S. saligna*	[68]
**90**	Iso-*N*-formylchonemorphine	R_1_ = R_4_ = R_5_ = H; R_2_ = R_3_ = CH_3_; R_6_ = CHO	*S. brevifolia*	[59]
**91**	Epipachysamine E	R_1_ = R_3_ = R_4_ = H; R_2_ = Sen; R_5_ = R_6_ = CH_3_	*Pachysandra terminalis*	[54]
**92**	11-Hydroxyepipachysamine E	R_1_ = R_2_ = R_4_ = H; R_3_ = Sen; R_5_ = R_6_ = CH_3_; 11-OH	*Sarcococca brevifolia*	[69]
**93**	Saligenamide C	R_1_ = *β*-OH; R_2_ = H; R_3_ = Tig; R_4_ = *β*-OAc; R_5_ = R_6_ = CH_3_; △^14,15^	*S. saligna*	[70]
**94**	Saligenamide F	R_1_ = R_4_ = H; R_2_ = CH_3_; R_3_ = COCHC(CH_3_)CH(CH_3_)CH_3_; R_5_ = R_6_ = CH_3_	*S. saligna*	[70]
**95**	2*β*-Hydroxyepipachysamine D	R_1_ = *β*-OH; R_2_ = R_4_ = R_5_ = H; R_3_ = Bz; R_6_ = CH_3_	*S. saligna*	[70]
**96**	Axillarine C	R_1_ = *β*-OH; R_2_ = H; R_3_ = Bz; R_4_ = *β*-OAc; R_5_ = R_6_ = CH_3_	*S. saligna*	[70]
**97**	Axillarine F	R_1_ = *β*-OH; R_2_ = H; R_3_ = Tig; R_4_ = *β*-OAc; R_5_ = R_6_ = CH_3_	*S. saligna*	[70]
**98**	Salonine A	R_1_ = *β*-OH; R_2_ = H; R_3_ = Tig; R_4_ = *β*-OH; R_5_ = R_6_ = CH_3_; △^14,15^	*S. saligna*	[50]
**99**	Dictyophlebine	R_1_ = R_2_ = R_4_ = H; R_3_ = R_5_ = R_6_ = CH_3_	*S. saligna*; *S. hookeriana*	[51, 65]
**100**	Hookerianamine A	R_1_ = R_2_ = R_4_ = H; R_3_ = R_5_ = R_6_ = CH_3_; △^14,15^	*S. hookeriana*	[71]
**101**	Isosarcodine	R_1_ = R_4_ = H; R_2_ = R_5_ = R_6_ = CH_3_; R_3_ = Ac	*S. saligna*	[72]
**102**	Hookerianamide B	R_1_ = *α*-OH; R_2_ = H; R_3_ = Sen; R_4_ = *β*-OH; R_5_ = R_6_ = CH_3_	*S. hookeriana*	[71]
**103**	Hookerianamide C	R_1_ = *β*-OAc; R_2_ = R_4_ = H; R_3_ = Sen; R_5_ = R_6_ = CH_3_	*S. hookeriana*	[71]
**104**	Hookerianamide D	R_1_ = R_4_ = H; R_2_ = R_5_ = CH_3_; R_3_ = COCHC(CH_3_)CH(CH_3_)CH_3_; R_6_ = CHO	*S. hookeriana*	[73]
**105**	Hookerianamide E	R_1_ = *β*-OAc; R_3_ = R_4_ = H; R_2_ = Sen; R_5_ = R_6_ = CH_3_; △^14,15^	*S. hookeriana*	[73]
**106**	Hookerianamide G	R_1_ = H; R_2_ = R_5_ = R_6_ = CH_3_; R_3_ = Bz; R_4_ = *β*-OAc	*S. hookeriana*	[73]
**107**	Hookerianamide I	R_1_ = R_4_ = R_5_ = H; R_2_ = R_6_ = CH_3_; R_3_ = Bz	*S. hookeriana*	[74]
**108**	Chonemorphine	R_1_ = R_2_ = R_3_ = R_4_ = H; R_5_ = R_6_ = CH_3_	*S. hookeriana*	[65]
**109**	*N*-Methypachysamine A	R_1_ = R_4_ = H; R_2_ = R_3_ = R_5_ = R_6_ = CH_3_	*S. hookeriana*	[65]
**110**	Pachysamine J	R_1_ = *α*-OH; R_2_ = R_4_ = H; R_3_ = Sen; R_5_ = R_6_ = CH_3_	*Pachysandra axillaris*	[75]
**111**	Pachysamine O	R_1_ = R_2_ = R_4_ = H; R_3_ = Cin; R_5_ = R_6_ = CH_3_	*P. axillaris*	[75]
**112**	Pachysamine P	R_1_ = R_2_ = H; R_3_ = COCH_2_C(CH_3_)C(CH_3_)CH_3_; R_4_ = *β*-OH; R_5_ = R_6_ = CH_3_	*P. axillaris*	[75]
**113**	(20*S*)-2*α*,4*β*-Bis(acetoxy)-20-(*N*,*N*-dimethylamino)-3*β*-tigloylamino-5*α*-pregnane	R_1_ = *α*-OAc; R_2_ = H; R_3_ = Tig; R_4_ = *β*-OAc; R_5_ = R_6_ = CH_3_	*Sarcococca hookeriana*	[76]
**114**	(20*S*)-20*α*-Cinnamoylamino-3*β*-dimethylamino-pregnane	R_1_ = R_4_ = R_5_ = H; R_2_ = R_3_ = CH_3_; R_6_ = COCH = CHph	*Pachysandra terminalis*	[63]
**115**	(20*S*)-(Bennzamido)-3*β*-(*N*,*N*-dimethyamino)-pregnane	R_1_ = R_4_ = H; R_2_ = R_3_ = R_6_ = CH_3_; R_5_ = Bz	*Sarcococca. saligna*	[77]
**116**	Sarcovagine D	R_1_ = Tig; R_2_ = O; R_3_ = R_4_ = H; R_5_ = CH_3_	*S. vegans*; *S. hookeriana*	[64, 65]
**117**	Sarcovagenine C	R_1_ = Tig; R_2_ = O;R_3_ = R_4_ = H; R_5_ = CH_3_; △^16,17^	*S. vegans*; *S. hookeriana*	[65, 78]
**118**	Axillaridine A	R_1_ = Bz; R_2_ = O; R_3_ = R_4_ = H; R_5_ = CH_3_	*S. saligna*; *P. procumbens*	[46, 69]
**119**	2,3-Dehydrosarsalignone	R_1_ = Tig; R_2_ = O; R_3_ = R_4_ = H; R_5_ = CH_3_; △^5,6^	*S. saligna*	[61]
**120**	14,15-Dehydrosarcovagine D	R_1_ = Tig; R_2_ = O; R_3_ = R_4_ = H; R_5_ = CH_3_; △^14,15^	*S. saligna*	[61]
**121**	Phulchowkiamide A	R_1_ = Tig; R_2_ = O; R_3_ = R_4_ = R_5_ = H	*S. hookeriana*	[72]
**122**	Hookerianamide F	R_1_ = Tig; R_2_ = O; R_3_ = R_4_ = R_5_ = H; △^14,15^	*S. hookeriana*	[71]
**123**	Hookerianamide H	R_1_ = CHO; R_2_ = O; R_3_ = R_4_ = H; R_5_ = CH_3_	*S. hookeriana*	[73]
**124**	(+)-(20*S*)-3-(Benzoylamino)-20-(dimethylamino)-5*α*-pregn-2-en-4*β*-yl acetate	R_1_ = Bz; R_2_ = *β*-OAc; R_3_ = R_4_ = H; R_5_ = CH_3_	*Pachysandra procumbens*	[46]
**125**	Pachysamine L	R_1_ = Tig; R_2_ = *β*-OAc; R_3_ = R_4_ = H; R_5_ = CH_3_	*P. axillaris*	[75]
**126**	Pachysamine M	R_1_ = Sen; R_2_ = O; R_3_ = R_4_ = H; R_5_ = CH_3_	*P. axillaris*	[75]
**127**	Pachysamine N	R_1_ = Sen; R_2_ = O; R_3_ = H; R_4_ = *β*-OH; R_5_ = CH_3_	*P. axillaris*	[75]
**128**	Sarsaligenine A	R_1_ = Sen; R_2_ = O; R_3_ = R_4_ = H; R_5_ = CH_3_; △^16,17^	*Sarcococca saligna*	[79]
**129**	Sarsaligenine B	R_1_ = Sen; R_2_ = O; R_3_ = *α*-OH; R_4_ = H; R_5_ = CH_3_	*S. saligna*	[79]
**130**	Sarcovagenines A	R_1_ = R_3_ = *β*-OH; R_2_ = Tig; R_4_ = CH_3_	*S. vegans*	[78]
**131**	Sarcovagenines B	R_1_ = *α*-OH; R_2_ = Tig; R_3_ = *β*-OAc; R_4_ = CH_3_	*S. vegans*	[78]
**132**	Salignarine D	R_1_ = R_3_ = H; R_2_ = Sen; R_4_ = CH_3_	*S. saligna*	[44]
**133**	(−)-Vaganine D	R_1_ = H; R_2_ = Sen; R_3_ = *β*-OAc; R_4_ = CH_3_	*S. coriacea*	[80]
**134**	(+)-Nepapakistamine A	R_1_ = R_3_ = *β*-OAc; R_2_ = Tig; R_4_ = H	*S. coriacea*	[80]
**135**	5,6-Dihydrosarconidine	R_1_ = R_3_ = H; R_2_ = R_4_ = CH_3_	*S. saligna*	[51]
**136**	16-Dehydrosarcorine	R_1_ = R_3_ = H; R_3_ = Ac; R_4_ = CH_3_	*S. saligna*	[61]
**137**	Hookerianamide A	R_1_ = R_3_ = *β*-OH; R_2_ = Sen; R_4_ = CH_3_	*S..hookeriana*	[72]
**138**	Saligenamide B	R_1_ = *β*-OH; R_2_ = Sen; △^14,15^	*S. saligna*	[67]
**139**	Salignarine E	R_1_ = H; R_2_ = Tig	*S. saligna*	[44]
**140**	Saligenamide D	R_1_ = *α*-OH; R_2_ = Tig; △^16,17^	*S. saligna*	[69]
**141**	2-Hydroxysalignarine E	R_1_ = *β*-OH; R_2_ = Tig	*S. saligna*	[51]
**142**	Salonine C	R_1_ = H; R_2_ = Tig; △^14,15^	*S. saligna*	[51]
**143**	*E*-salignone		*S. saligna*	[65]
**144**	*Z*-salignone		*S. saligna*	[65]
**145**	Holamine	R_1_ = *α*-NH_2_; R_2_ = R_3_ = R_4_ = H; R_5_ = R_6_ = *α*-H; △^5,6^	*Holarrhena curtisii*	[81]
**146**	3*α*-Amino-14*β*-hydroxypregnan-20-one	R_1_ = *α*-NH_2_; R_2_ = R_3_ = R_4_ = R_6_ = H; R_5_ = *β*-OH	*H. curtisii*	[81]
**147**	15*α*-Hydroxyholamine	R_1_ = *α*-NH_2_; R_2_ = R_3_ = R_4_ = H; R_5_ = *α*-H; R_6_ = *α*-OH; △^5,6^	*H. curtisii*	[81]
**148**	Pachysanone	R_1_ = O; R_2_ = H; R_3_ = *α*-OCOCH_2_C(CH_3_)C(CH_3_)CH_3_; R_4_ = *β*-OAc; R_5_ = R_6_ = H	*Pachysandra axillaris*	[45]
**149**	Pachysanonin	R_1_ = *β*-N(CH_3_)_2_; R_2_ = H; R_3_ = *α*-OCOCH_2_C(CH_3_)C(CH_3_)CH_3_; R_4_ = *β*-OAc; R_5_ = R_6_ = H	*P. axillaris*	[45]
**150**	Pachysamine Q	R_1_ = *β*-N(CH_3_)_2_; R_2_ = R_4_ = *β*-OAc; R_3_ = *α*-OCO-Bn; R_5_ = R_6_ = H	*P. axillaris*	[75]
**151**	Pachysamine R	R_1_ = *β*-N(CH_3_)_2_; R_2_ = R_4_ = *β*-OAc; R_3_ = *α*-OCOCH_2_C(CH_3_)C(CH_3_)CH_3_; R_5_ = R_6_ = H	*P. axillaris*	[75]
**152**	Terminamine F	R_1_ = *α*-NCH_3_-Sen; R_2_ = R_3_ = R_4_ = R_5_ = R_6_ = H	*P. terminalis*	[82]
**153**	Terminamine G	R_1_ = *α*-NCH_3_-Bz; R_2_ = R_3_ = R_4_ = R_5_ = R_6_ = H	*P. terminalis*	[82]
**154**	Funtumine	R_1_ = *α*-NH_2_; R_2_ = R_3_ = R_4_ = R_5_ = R_6_ = H	*Holarrhena floribunda*	[83]
**155**	(+)-(20*S*)-20-(Dimethylamino)-3-(3′*α*-isopropyl)-lactam-5*α*-pregn-2-en-4-one	R_1_ = R_3_ = R_4_ = H; R_2_ = O; △^1,2^	*Pachysandra procumbens*	[46]
**156**	(+)-(20*S*)-20-(Dimethylamino)-16*α*-hydroxy-3-(3′*α*-isopropyl)-lactam-5*α*-pregn-2-en-4-one	R_1_ = R_3_ = H; R_2_ = O; R_4_ = *α*-OH; △^1,2^	*P. procumbens*	[46]
**157**	Pachystermine A	R_1_ = R_3_ = R_4_ = H; R_2_ = O	*P. terminalis*	[54]
**158**	(+)-(20*S*)-20-(Dimethylamino)-16*α*-hydroxy-3*β*-(3′*α*-isopropyl)-lactam-5*α*-pregn-4-one	R_1_ = R_3_ = H; R_2_ = O; R_4_ = *α*-OH	*P. procumbens*	[55]
**159**	Terminamine A	R_1_ = R_3_ = H; R_2_ = O; R_4_ = *β*-OH	*P. terminalis*	[82]
**160**	Terminamine B	R_1_ = *β*-OAc; R_2_ = R_4_ = *β*-OH; R_3_ = *α*-OAc	*P. terminalis*	[82]
**161**	Terminamine C	R_1_ = *β*-OAc; R_2_ = R_4_ = *β*-OH; R_3_ = val	*P. terminalis*	[82]
**162**	Pachystermine B	R_1_ = R_3_ = R_4_ = H; R_2_ = *β*-OH	*P. terminalis*	[82]
**163**	Terminamine D	R = OH	*P. terminalis*	[82]
**164**	Terminamine E	R = H	*P. terminalis*	[82]
**165**	(+)-(20*S*)-2*α*-Hydroxy-20-(dimethylamino)-3*β*-phthalimido-5*α*-pregnan-4*β*-yl acetate		*P. procumbens*	[46]
**166**	Spiropachysine		*P. procumbens*	[46]
**167**	Salignarine A		*Sarcococca saligna*	[44]
**168**	Epoxynepapakistamin A	R_1_ = R_3_ = *β*-OAc; R_2_ = *β*-NH-Tig; R_4_ = H	*S. coriacea*	[47]
**169**	Epoxysarcovagenine D	R_1_ = R_4_ = H; R_2_ = *β*-NH-Tig; R_3_ = O; △^2,3^	*S. coriacea*	[47]
**170**	(*S*)-20-(*N*,*N*-Dimethylamino)-16*α*,17*α*-epoxy-3*β*-methoxy-pregn-5-ene	R_1_ = R_3_ = R_4_ = H; R_2_ = *β*-OCH_3_; △^5,6^	*S. hookeriana*	[76]
**171**	Hookerianine B	R_1_ = R_3_ = H; R_2_ = *α*-NH-Bz; R_4_ = CH_3_	*S. hookeriana*	[56]
**172**	*N*-methylfuntumafrine		*S. coriacea*	[47]
**173**	Pachysamines K	R_1_ = R_4_ = H; R_2_ = *α*-OH; R_3_ = Bz; R_5_ = *α*-OH	*Pachysandra axillaris*	[75]
**174**	Archosokloide A	R_1_ = R_5_ = H; R_2_ = *β*-OH; R_3_ = Tig; R_4_ = *β*-OH	*Sarcococca hookeriana*	[57]
**175**	Sarchookloide B	R_1_ = R_4_ = R_5_ = H; R_2_ = *β*-OH; R_3_ = Tig	*S. hookeriana*	[57]
**176**	4-Dehydroxyepisarcovagine A	R_1_ = *β*-OH; R_2_ = R_4_ = R_5_ = H; R_3_ = Tig; △^14,15^	*S. pruniformis*	[84]
**177**	(20*S*)-(Bennzamido)-pregnane-3-one		*S. saligna*	[77]

For alkaloids with nitrogen substituents at C-3, only **53–62** and **172–176** bear 3*α* substituents, whereas most compounds possess the 3*β* configuration. All of them except **143–154** contain nitrogen substituents at C-20, which is a common feature of pregnane type alkaloids. Salignarine A **(167)** has a novel structure with an epoxide functionality at C-5–C-6 [44]. Two compounds, pachysanone **(148)** and pachysanonin **(149)** bear a 3,4-dimethylpent-3-enoyloxy substituent at C-11, a rare functional group in natural products [45]. Compounds **155–164**, bear a (3′sopropyl)-*β*-lactam ring at the C-3 position, whereas compound **165** bears a phthalimido moiety at the same position [46]. Spiropachysine **(166)** possesses a five membered-ring spiro-lactam and a disubstituted benzene ring at C-3 [46]. Compounds **168–171** display a structural modification that has not been reported from this genus, viz. the epoxy ring at C-16/C-17. *N*-methylfuntumafrine **(172)** shows a novel structure with an acetyl group at C-17 [47].

#### Cyclopregnane alkaloids

The *Buxus* genus of the Buxaceae family is a rich source of cyclopregnane alkaloids, and 116 cyclopregnane alkaloids (**178–293**) have been reported from *B. sempervirens, B. longifolia, B. hildebrandtii, B. bodinieri, B. hyrcana, B. microphylla, B. papillosa, B. wallichiana*, *B. rugulosa, B. natalensis,* and *B. macowanii.*

Cyclopregnane alkaloids, also known as triterpenoid alkaloids, possess a unique pregnane type structure with C-4 methyl groups, a 9*β*,10*β*-cycloartenol system, and a degraded C-20 side chain. All alkaloids possess a nitrogen function at C-3 and/or C-20, which may be unmethylated, partially methylated, or fully methylated. Structurally, the majority of these alkaloids contain either a 9*β*,19-cyclo-14*α*-methylpregnane type or a 9(10 → 19)abeo-14*α*-methylpregnane type, having a characteristic substituent pattern at C-4.

##### 9*β*,19-Cyclo-14*α*-methylpregnane type

**Fig. 5:**
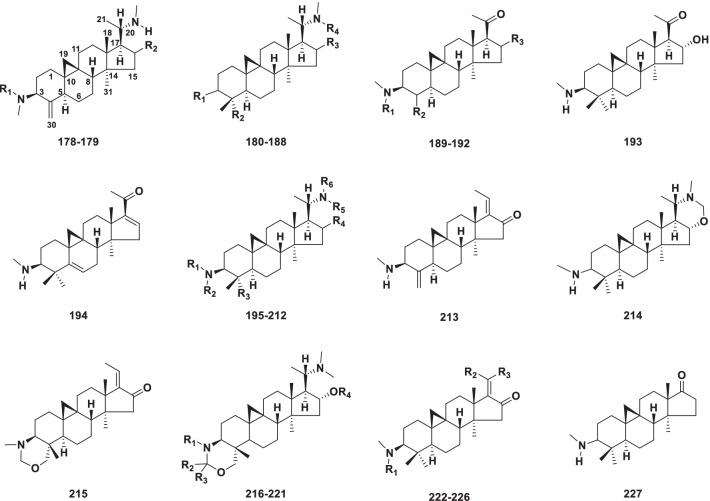
Structures of 9*β*,19-cyclo-14*α*-methylpregnane type steroidal alkaloids **178–227**

Out of the 116 cyclopregnane alkaloids, 50 (**178–227)** belong to this type, which are characteristic of the genus *Buxus* (Table 4). This type of compound is characterized by a pentacyclic 4,4,14-trimethyl-9,19-cyclopregnane skeleton (Fig. 5).

**Table 4:** Structures and sources of 9*β*,19-Cyclo-14*α*-methylpregnane type steroidal alkaloids **178–227**

No	Compounds	Substitution groups and others	Sources	References
**178**	Cyclobuxine	R_1_ = H; R_2_ = *α*-OH	*Buxus sempervirens*	[85]
**179**	Cyclobuxamidine	R_1_ = Ac; R_2_ = H	*B. longifolia*	[86]
**180**	Cyclobuxoviridine	R_1_ = O; R_2_ = R_4_ = CH_3_; R_3_ = H; △^1,2^	*B. hildebrandtii*	[91]
**181**	Buxbodine A	R_1_ = R_3_ = H; R_2_ = R_4_ = CH_3_	*B. bodinieri*	[87]
**182**	Buxbodine B	R_1_ = O; R_2_ = R_4_ = CH_3_; R_3_ = *β*-OH; △^1,2^	*B. bodinieri*	[87]
**183**	*N*_b_-Demethylcyclomikurane	R_1_ = O; R_2_ = CH_3_; R_3_ = R_4_ = H	*B. sempervirens*	[92]
**184**	Cyclimikuranine	R_1_ = O; R_2_ = R_4_ = CH_3_; R_3_ = H	*B. sempervirens*	[92]
**185**	*N*_b_-Demethylcyclobuxoviricine	R_1_ = O; R_2_ = R_4_ = CH_3_; R_3_ = *α*-OH; △^1,2^	*B. hyrcana*	[93]
**186**	Buxmicrophylline K	R_1_ = *β*-OH; R_2_ = CH_3_; R_3_ = *α*-OH; R_4_ = H	*B. microphylla*	[88]
**187**	*N*-Demethylcyclomikuranine	R_1_ = O; R_2_ = CH_3_; R_3_ = *α*-OH; R_4_ = H	*B. microphylla*	[88]
**188**	31-Demethylcyclobuxoviridine	R_1_ = O; R_2_ = H; R_3_ = H; R_4_ = CH_3_; △^1,2^	*B. hyrcana*	[94]
**189**	Cyclomicrobuxamine	R_1_ = R_3_ = H; R_2_ = CH_2_	*B. hildebrandtii*	[91]
**190**	Cyclomicrobuxeine	R_1_ = R_3_ = H; R_2_ = CH_2_; △^16,17^	*B. sempervirens*	[95]
**191**	30-Hydroxycyclomicobuxene	R_1_ = R_3_ = H; R_2_ = CH_2_OH; △^4,5^	*B. sempervirens*	[96]
**192**	Buxippine K	R_1_ = CH_3_; R_2_ = CH_2_; R_3_ = *α*-OH	*B. hyrcana*	[93]
**193**	Cyclorolfeine		*B. hildebrandtii*	[91]
**194**	Cyclobuxomicreinine		*B. longifolia*	[97]
**195**	Isodihydrocyclomicrophylline A	R_1_ = R_2_ = R_5_ = R_6_ = CH_3_; R_3_ = CH_2_OH; R_4_ = *α* -OH	*B. sempervirens*	[98]
**196**	Buxasamarine	R_1_ = R_2_ = R_3_ = CH_3_; R_4_ = *α*-OH; R_5_ = H; R_6_ = CH_3_; △^1,2^	*B. longifolia*	[86]
**197**	Buxmicrophylline C	R_1_ = H; R_2_ = Isobu; R_3_ = CH_2_OH; R_4_ = *α*-OH; R_5_ = R_6_ = CH_3_; △^6,7^	*B. microphylla*	[99]
**198**	Buxbodine D	R_1_ = R_2_ = R_3_ = CH_3_; R_4_ = R_5_ = H; R_6_ = Ac; △^6,7^	*B. bodinieri*	[87]
**199**	Buxbodine E	R_1_ = R_2_ = R_3_ = CH_3_; R_4_ = R_5_ = R_6_ = H; △^6,7^	*B. bodinieri*	[87]
**200**	Buxakashmiramine	R_1_ = Syr; R_2_ = R_5_ = R_6_ = CH_3_; R_3_ = CH_2_OH; R_4_ = H	*B. papillosa*	[100]
**201**	Cycloprotobuxine C	R_1_ = R_2_ = R_3_ = R_5_ = R_6_ = CH_3_; R_4_ = H; △^6,7^	*B. papillosa*	[100]
**202**	Cyclovirobuxeine A	R_1_ = R_2_ = R_3_ = R_5_ = R_6_ = CH_3_; R_4_ = *α*-OH; △^6,7^	*B. papillosa*	[100]
**203**	Cyclovirobuxine D	R_1_ = R_5_ = H; R_2_ = R_3_ = R_6_ = CH_3_; R_4_ = *α*-OH	*B. wallichiana*	[101]
**204**	Hyrcamine	R_1_ = H R_2_ = Tig; R_3_ = CH_2_OH; R_4_ = *α* -OAc; R_5_ = R_6_ = CH_3_	*B. hyrcana*	[93]
**205**	Buxidine	R_1_ = H R_2_ = Bz; R_3_ = CH_2_OH; R_4_ = *α*-OH; R_5_ = R_6_ = CH_3_; △^6,7^	*B. hyrcana*	[93]
**206**	Buxandrine	R_1_ = H R_2_ = Bz; R_3_ = CH_2_OH; R_4_ = *α* -OAc; R_5_ = R_6_ = CH_3_; △^6,7^	*B. hyrcana*	[93]
**207**	Buxrugulosamine	R_1_ = H; R_2_ = Ac; R_3_ = CH_3_; R_4_ = H; R_5_ = H; R_6_ = CH_3_	*B.rugulosa*	[102]
**208**	Buxmicrophylline E	R_1_ = H; R_2_ = Bz; R_3_ = CH_2_OH; R_4_ = O-Bz; R_5_ = R_6_ = CH_3_	*B. microphylla*	[103]
**209**	Buxmicrophylline F	R_1_ = H; R_2_ = Isobu; R_3_ = CH_2_OH; R_4_ = O-Bz; R_5_ = R_6_ = CH_3_; △^6,7^	*B. microphylla*	[103]
**210**	Buxmicrophylline G	R_1_ = H; R_2_ = Isofer; R_3_ = CH_2_OH; R_4_ = *α* -OH; R_5_ = R_6_ = CH_3_	*B. microphylla*	[103]
**211**	Buxmicrophylline H	R_1_ = H; R_2_ = Syr; R_3_ = CH_2_OH; R_4_ = *α* -OH; R_5_ = R_6_ = CH_3_	*B. microphylla*	[103]
**212**	Cyclonataminol	R_1_ = R_2_ = R_3_ = R_5_ = R_6_ = CH_3_; R_4_ = *α*-OH; 2-*α*-OH; △^6,7^	*B. natalensis*	[104]
**213**	*trans*-Cyclosuffrobuxinine		*B. longifolia*	[86]
**214**	Buxozine C		*B. papillosa*	[90]
**215**	Sempervirone		*B. papillosa*	[89]
**216**	Buxmicrophylline D	R_1_ = R_2_ = CH_3_; R_3_ = R_4_ = H	*B. microphylla*	[99]
**217**	Buxmicrophylline I	R_1_ = R_2_ = R_3_ = H; R_4_ = Syr	*B. microphylla*	[103]
**218**	Buxmicrophylline J	R_1_ = H; R_2_ = R_3_ = CH_3_; R_4_ = Bz	*B. microphylla*	[88]
**219**	Buxmicrophylline P	R_1_ = R_2_ = H; R_3_ = *β*-CH_3_; R_4_ = H	*B. microphylla*	[105]
**220**	Buxmicrophylline Q	R_1_ = R_2_ = H; R_3_ = *β*-CH_3_; R_4_ = Syr	*B. microphylla*	[105]
**221**	Buxmicrophylline R	R_1_ = R_2_ = H; R_3_ = *β*-CH_3_; R_4_ = Van	*B. microphylla*	[105]
**222**	Buxmicrophylline B	R_1_ = R_3_ = H; R_2_ = CH_3_	*B. microphylla*	[99]
**223**	*E*-cyclobuxaphylamine	R_1_ = R_3_ = H; R_2_ = CH_3_; △^7,8^	*B. sempervirens*	[95]
**224**	*Z*-cyclobuxaphylamine	R_1_ = R_2_ = H; R_3_ = CH_3_; △^7,8^	*B. sempervirens*	[95]
**225**	Buxbodine C	R_1_ = R_2_ = CH_3_; R_3_ = H; △^6,7^	*B. bodinieri*	[87]
**226**	Cyclobuxaphylline	R_1_ = R_2_ = CH_3_; R_3_ = H	*B. sempervirens*	[92]
**227**	17-Oxocycloprotobuxine		*B. sempervirens*	[106]

Cyclobuxine D **(178)** was the first steroidal alkaloid from *Buxus* bearing a C-4 methylene substituent [85]. Later, cyclobuxamidine **(179)** and trans-cyclosuffrobuxinine **(213)** of this type were isolated from *B. longifolia* [86]*.* Buxbodine A **(181)** has a unique structure due to the lack of a keto or an amino functionality at the C-3 position [87]. Typically, all alkaloids of this type have a C-3 amino or carbonyl group, except for buxmicrophylline K **(186)**, which has a hydroxyl group substituted at C-3 [88]. In compounds *trans*-cyclosuffrobuxinine **(213)** and sempervirone **(215),** the methylamino group at C-20 is eliminated and the secondary alcohol at C-16 is oxidized [86, 89]. Buxozine C **(214)** with a tetrahydro-oxazine ring joining the C-16*α* and the C-20 nitrogen has been isolated from *B. papillosa.* The mass spectra of compound **214** exhibit the ions at m/z 127 and 113 due to cleavage of ring D, and these ions serve as diagnostic features to determine the presence of a tetrahydro-oxazine ring in ring D [90].

##### 9(10 → 19)Abeo-14*α*-methylpregnane alkaloids

**Fig. 6:**
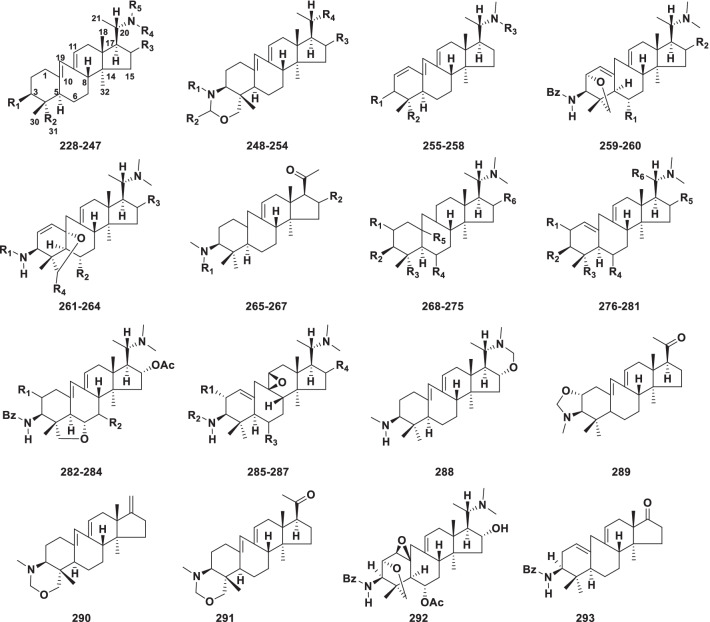
Structures of 9(10 → 19)abeo-14*α*-methylpregnane type steroidal alkaloids **228–293**

This type contains a tetracyclic system in which 9,19 bond fission has occurred to give seven-membered ring B (Fig. 6). Sixty-six alkaloids (**228–293)** were isolated from the genus *Buxus* (Table 5).

**Table 5:** Structures and sources of 9(10 → 19)abeo-14α-methylpregnane type steroidal alkaloids **228–293**

No	Compounds	Substitution groups and others	Sources	References
**228**	Buxamine A	R_1_ = N(CH_3_)_2_; R_2_ = R_4_ = R_5_ = CH_3_; R_3_ = H	*Buxus hildebrandtii*; *B. natalensis*	[91, 104]
**229**	Buxamine C	R_1_ = NHCH_3_; R_2_ = R_4_ = R_5_ = CH_3_; R_3_ = H	*B. hildebrandtii*	[91]
**230**	30-*O*-benzoyl-16-deoxybuxidienine C	R_1_ = H; R_2_ = CH_2_O-Bz; R_3_ = H; R_4_ = R_5_ = CH_3_	*B. hildebrandtii*	[91]
**231**	30-Hydroxybuxamine A	R_1_ = R_4_ = R_5_ = CH_3_; R_2_ = CH_2_OH; R_3_ = H	*B.hildebrandtii*	[91]
**232**	30-Norbuxamine A	R_1_ = R_4_ = R_5_ = CH_3_; R_2_ = R_3_ = H	*B.hildebrandtii*	[91]
**233**	*N*-benzoyl-*O*-acetylbuxalongifoline	R_1_ = NH-Bz; R_2_ = COH; R_3_ = *α*-OH; R_4_ = R_5_ = CH_3_	*B. longifolia*	[86]
**234**	16*α*-Acetoxy-buxabenzamidienine	R_1_ = NH-Bz; R_2_ = R_4_ = R_5_ = CH_3_; R_3_ = *α*-OAc	*B. longifolia*	[86]
**235**	Buxaminol C	R_1_ = NHCH_3_; R_2_ = R_4_ = R_5_ = CH_3_; R_3_ = *α*-OH	*B. sempervirens*	[95]
**236**	Papilamine	R_1_ = NHCH_3_; R_2_ = R_5_ = CH_3_; R_3_ = R_4_ = H	*B. sempervirens*	[95]
**237**	16*α*-Hydro-xypapillamidine	R_1_ = NH-Ac; R_2_ = R_4_ = R_5_ = CH_3_; R_3_ = *α*-OH	*B. papillosa*	[109]
**238**	(+)-Benzoylbuxidienine	R_1_ = NH-Bz; R_2_ = R_4_ = R_5_ = CH_3_; R_3_ = *α*-OH	*B. hyrcana*	[110]
**239**	Buxamine F	R_1_ = NH_2_; R_2_ = R_4_ = R_5_ = CH_3_; R_3_ = H	*B. sempervirens*	[106]
**240**	*N*_20_-Formylbuxaminol E	R_1_ = N(CH_3_)_2_; R_2_ = CH_3_; R_3_ = *α*-OH; R_4_ = H; R_5_ = COH	*B. sempervirens*	[111]
**241**	*O*_16_-syringylbuxaminol E	R_1_ = N(CH_3_)_2_; R_2_ = CH_3_; R_3_ = *α*-O-Syr; R_4_ = R_5_ = H	*B. sempervirens*	[111]
**242**	*N*_20_-acetylbuxamine G	R_1_ = NHCH_3_; R_2_ = CH_3_; R_3_ = H; R_4_ = H; R_5_ = Ac	*B. sempervirens*	[111]
**243**	*N*_20_-acetylbuxamine E	R_1_ = N(CH_3_)_2_; R_2_ = CH_3_; R_3_ = H; R_4_ = H; R_5_ = Ac	*B. sempervirens*	[111]
**244**	Buxakarachiamine	R_1_ = NH-COCH(OH)CH(CH_3_)_2_; R_2_ = CH_2_OH; R_3_ = H; R_4_ = R_5_ = CH_3_	*B. papillosa*	[100]
**245**	Buxahejramine	R_1_ = NH-COCH(OH)CH(CH_3_)CH_2_CH; R_2_ = CH_2_OH; R_3_ = H; R_4_ = R_5_ = CH_3_	*B. papillosa*	[100]
**246**	31-Demethylbuxaminol A	R_1_ = N(CH_3_)_2_; R_2_ = H; R_3_ = *α*-OH; R_4_ = R_5_ = CH_3_	*B. natalensis*	[104]
**247**	Buxaminol A	R_1_ = N(CH_3_)_2_; R_2_ = R_4_ = R_5_ = CH_3_; R_3_ = *α*-OH	*B. natalensis*	[104]
**248**	Moenjodarmine	R_1_ = CH_3_; R_2_ = R_3_ = H; R_4_ = N(CH_3_)_2_	*B. hildebrandtii*; *B. hyrcana*	[91, 112]
**249**	*N*_b_-Demethylharapamine	R_1_ = CH_3_; R_2_ = R_3_ = H; R_4_ = NH_2_	*B. papillosa*	[113]
**250**	Homomoenjodaramine	R_1_ = R_2_ = CH_3_; R_3_ = H; R_4_ = N(CH_3_)_2_	*B. hyrcana*	[112]
**251**	Buxhyrcamine	R_1_ = R_2_ = R_3_ = H; R_4_ = N(CH_3_) _2_	*B. hyrcana*	[94]
**252**	Macowanioxazine	R_1_ = CH_3_; R_2_ = H; R_3_ = *α*-OH; R_4_ = N(CH_3_) _2_	*B. macowanii*	[114]
**253**	16*α*-Hydroxymacowanitriene	R_1_ = CH_3_; R_2_ = H; R_3_ = *α*-OH; R_4_ = N(CH_3_) _2_; △^1,2^	*B. macowanii*	[114]
**254**	Macowanitriene	R_1_ = CH_3_; R_2_ = R_3_ = H; R_4_ = N(CH_3_) _2_; △^1,2^	*B. macowanii*	[114]
**255**	Papillotrienine	R_1_ = *β*-NHCH_3_; R_2_ = R_3_ = CH_3_	*B. papillosa*	[113]
**256**	*N*_b_-Demethylpapilliotrienine	R_1_ = *β*-NHCH_3_; R_2_ = CH_3_; R_3_ = H	*B. papillosa*	[113]
**257**	Hyrcatrienine	R_1_ = *β*-NH-Bz; R_2_ = R_3_ = CH_3_	*B. hyrcana*	[93]
**258**	31-Hydroxybuxatrienone	R_1_ = O; R_2_ = CH_2_OH; R_3_ = CH_3_	*B. macowanii*	[114]
**259**	*O*^2^-Buxafuranamine	R_1_ = H; R_2_ = H	*B. hildebrandtii*	[115]
**260**	6-Hydroxy-*O*^2^-buxafuranamine	R_1_ = OH; R_2_ = H	*B. hildebrandtii*	[115]
**261**	*O*^10^-Buxafurana-mine	R_1_ = Bz; R_2_ = OH; R_3_ = R_4_ = H	*B. hildebrandtii*	[115]
**262**	*O*^10^-Natafuranamine	R_1_ = Bz; R_2_ = OH; R_3_ = *α*-OH; R_4_ = H	*B. natalensis*	[104]
**263**	Buxusemine B	R_1_ = Bz; R_2_ = OH; R_3_ = *α*-OH; R_4_ = O	*B. sempervirens*	[108]
**264**	Buxusemine C	R_1_ = Bz; R_2_ = R_3_ = R_4_ = H	*B. sempervirens*	[108]
**265**	(−)-16-Hydroxybuxaminone	R_1_ = CH_3_; R_2_ = *α*-OH; △^10,19^	*B. sempervirens*	[97]
**266**	Semperviraminone	R_1_ = CH_3_; R_2_ = H; △^1,10^; △^16,17^	*B. sempervirens*	[95]
**267**	*N*_a_-Demethylsemperviraminone	R_1_ = R_2_ = H; △^1,10^; △^16,17^	*B. sempervirens*	[95]
**268**	Buxalongifolamidine	R_1_ = R_4_ = H; R_2_ = NH-Bz; R_3_ = CH_2_OH; R_5_ = *α*-OH; R_6_ = *α*-OAc; △^1,2^; △^9,11^	*B. longifolia*	[107]
**269**	Semperviraminol	R_1_ = *α*-OH; R_2_ = NH-Bz; R_3_ = CH_3_; R_4_ = *β*-OAc; R_5_ = /; R_6_ = H; △^1,10^	*B. sempervirens*; *B. papillosa*	[100, 106]
**270**	*N*-Benzoylbuxahyrcanine	R_1_ = R_4_ = R_6_ = H; R_2_ = NH-Bz; R_5_ = *β*-OH; R_3_ = CH_3_; △^9,11^	*B. hyrcana*	[116]
**271**	*N*-Tigloylbuxahyrcanine	R_1_ = R_4_ = R_6_ = H; R_2_ = NH-Tig; R_3_ = CH_3_; R_5_ = *β*-OH; △^9,11^	*B. hyrcana*	[116]
**272**	*N*-Isobutyroyl-buxahyrcanine	R_1_ = R_4_ = R_6_ = H; R_2_ = Isobu; R_3_ = CH_3_; R_5_ = *β*-OH; △^9,11^	*B. hyrcana*	[116]
**273**	Hyrcanone	R_1_ = R_4_ = R_6_ = H; R_2_ = NH-Bz; R_3_ = CH_3_; R_5_ = /; 11-O; △^1,10^	*B. hyrcana*	[93]
**274**	2*α*,16*α*,31-Triacetyl-9,11-dihydrobuxiran	R_1_ = R_6_ = *α*-OAc; R_2_ = NH-Bz; R_3_ = CH_2_ OAc; R_4_ = H; R_5_ = /; △^1,10^	*B. hyrcana*	[117]
**275**	Macowamine	R_1_ = R_4_ = R_5_ = R_6_ = H; R_2_ = NCH_3_-Van; R_3_ = CH_2_OH; △^9,11^	*B. macowanii*	[114]
**276**	16*α*-Hydroxy-*N*_a_-benzoylbuxadine	R_1_ = R_4_ = H; R_2_ = NH-Bz; R_3_ = CH_3_; R_5_ = *α*-OH; R_6_ = OH	*B. sempervirens*	[95]
**277**	*N*_b_-Dimethylbuxupapine	R_1_ = R_4_ = R_5_ = H; R_2_ = N(CH_3_)_2_; R_3_ = R_6_ = CH_3_	*B. papillosa*	[109]
**278**	(+)-16*α*,31-Diacetylbuxadine	R_1_ = R_4_ = H; R_2_ = NH-Bz; R_3_ = CH_2_OAc; R_5_ = *α*-OAc; R_6_ = CH_3_	*B. sempervirens*	[92]
**279**	Hyrcanol	R_1_ = *β*-OH; R_2_ = NH-Bz; R_3_ = R_6_ = CH_3_; R_4_ = *α*-OAc; R_5_ = H	*B. hyrcana*	[93]
**280**	Buxabenzacinine	R_1_ = R_3_ = H; R_2_ = NH-Bz; R_4_ = CH_2_OAc; R_5_ = *α*-OH; R_6_ = CH_3_	*B. hyrcana*	[93]
**281**	2*α*,16*α*,31-Triacetylbuxiran	R_1_ = *α*-OAc; R_2_ = NH-Bz; R_3_ = H; R_4_ = CH_2_OAc; R_5_ = *α*-OAc; R_6_ = CH_3_	*B. hyrcana*	[117]
**282**	Cyclovirobuxeine F	R_1_ = R_2_ = H	*B. longifolia*	[86]
**283**	(+)-*O*^6^-Buxafurandiene	R_1_ = *α*-OH; R_2_ = *β*-OH	*B. hyrcana*	[110]
**284**	(+)-7-deoxy-*O*^6^-Buxafurandiene	R_1_ = *α*-OH; R_2_ = *β*-H	*B. hyrcana*	[110]
**285**	2*α*,16*α*-Diacetoxy-9*β*,11b-epoxybuxamidine	R_1_ = OAc; R_2_ = Bz; R_3_ = H; R_4_ = *α*-OAc	*B. papillosa*	[89]
**286**	Buxapapillinine	R_1_ = OAc; R_2_ = Bz; R_3_ = *α*-OAc; R_4_ = H	*B. sempervirens*; *B. hyrcana*	[106, 110]
**287**	Buxusemine D	R_1_ = R_4_ = H; R_2_ = Bz; R_3_ = *α*-OAc	*B. sempervirens*	[108]
**288**	Papillozine C		*B. papillosa*	[89]
**289**	Sempervirooxazolidine		*B. sempervirens*	[96]
**290**	Hyrcanine		*B. hyrcana*	[112]
**291**	Buxaquamarine		*B. hyrcana*	[110]
**292**	*O*^2^-Natafuranamine		*B. natalensis*	[104]
**293**	17-Oxo-3-benzoylbuxadine		*B. hyrcana*	[94]

Alkaloids **248–254** and **290–291** are members of the class having a tetrahydro-oxazine moiety incorporated in ring A, while in compound **288** an oxazine ring is attached to ring D [89]. The presence of this ring can be easily recognized by the ^1^H NMR spectrum exhibiting the presence of two pairs of AB doublets at δ 3.20–4.50 [91]. Compounds **255–258** belong to a unique class having a conjugated triene system at △^1,2^, △^10,19^ and △^9,11^. Compounds **259–264**, **282–284** and **292** belong to the rarely occurring class having an additional tetrahydrofuran ring incorporated in their structures through the ether linkage between C-10, C-2, or C-10 and C-23. Buxalongifolamidine (**268**) and **270–272** containing a hydroxyl group at C-10 may support the plausible biosynthesis of the ether linkage in these alkaloids [107]. Compounds **285–287** and **292** are rare cyclopregnane alkaloids featuring an epoxy motif [108]. Sempervirooxazolidine (**289**) also represents a novel structure having an oxazolidine moiety incorporated in its structure at C-2 and C-3 [96]. The compound 17-Oxo-3-benzoylbuxadine (**293**) having a carbonyl group at C-17 has been isolated from *B. hyrcana* [94].

#### Cholestane alkaloids

Based on the carbon framework, C_27_ alkaloids can be divided into two types: C-nor-D-homosteroidal alkaloids and cholestane alkaloids. The former, usually referred to as *Veratrum* steroidal alkaloids, characterised by a five-membered C-ring and six-membered D-ring system, can be further divided into cevanine, veratramine, and jervine types. The latter, usually named *Solanum* steroidal alkaloids, containing the common ABCD steroid skeleton, generally occurring as glycosides, can be grouped into spirosolane, solanidine and verazine types.

A total of 310 new members (**294–603)** were derived mainly from the genus *Solanum* in the Solanaceae family, and the genera *Veratrum* and *Fritillaria* in Liliaceae family.

##### Cevanine type

**Fig. 7:**
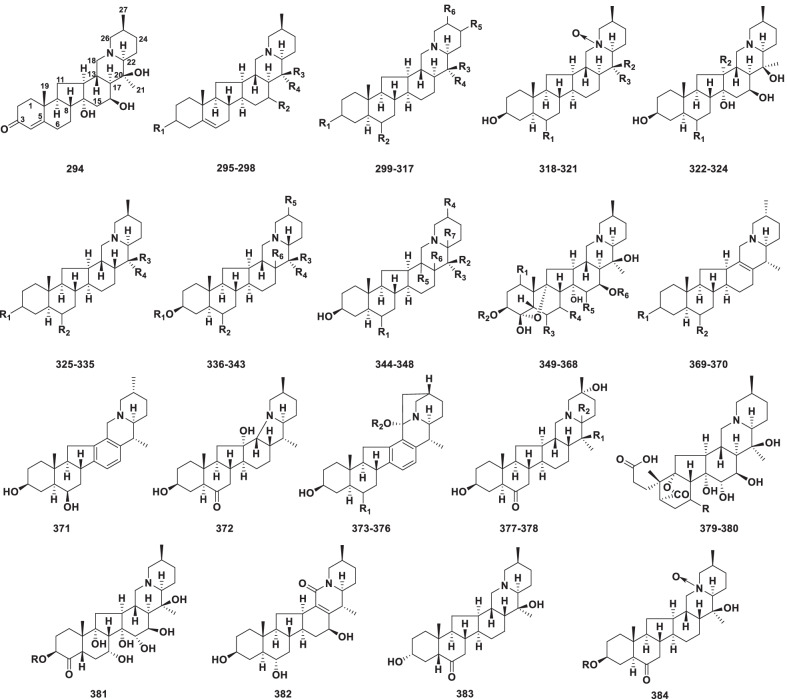
Structures of cevanine type steroidal alkaloids **294–384**

Members of the cevanine type are characterized by the hexacyclic benzo [7, 8] fluoreno[2,1-*β*]quinolizine nucleus (Fig. 7) [15]. This type is the largest representative group of C-nor-D-homosteroidal alkaloids, and currently comprises 91 new members (**294–384)** from *Veratrum* and *Fritillaria* genera in the Liliaceae family (Table 6).

**Table 6:** Structures and sources of cevanine type steroidal alkaloids **294–384**

No	Compounds	Substitution groups and others	Sources	References
**294**	Veratrenone		*Veratrum album*	[118]
**295**	Shinonomenine	R_1_ = *β*-OH; R_2_ = R_4_ = H; R_3_ = CH_3_	*V. grandiflorum*	[127]
**296**	Veraflorizine	R_1_ = *β*-OH; R_2_ = H; R_3_ = OH; R_4_ = CH_3_	*V. grandiflorum*	[127]
**297**	Fritillarizine	R_1_ = *α*-OH; R_2_ = H; R_3_ = OH; R_4_ = CH_3_	*Fritillaria verticillata*	[128]
**298**	Veramarine-3-yl formate	R_1_ = *β*-OCOH; R_2_ = *β*-OH; R_3_ = OH; R_4_ = CH_3_	*Veratrum nigrum*	[129]
**299**	Baimonidine:	R_1_ = *α*-OH; R_2_ = *β*-OH; R_3_ = OH; R_4_ = CH_3_; R_5_ = H; R_6_ = *β*-CH_3_	*Fritillaria verticillata*	[130]
**300**	Isoverticine	R_1_ = R_2_ = *β*-OH; R_3_ = OH; R_4_ = CH_3_; R_5_ = H; R_6_ = *β*-CH_3_	*F. verticillata*	[130]
**301**	Isobaimonidine	R_1_ = R_2_ = *α*-OH; R_3_ = OH; R_4_ = CH_3_; R_5_ = H; R_6_ = *β*-CH_3_	*F. verticillata*	[119]
**302**	3-*β*-d-Petilinineglucoside	R_1_ = d-Glc; R_2_ = *α*-OH; R_3_ = H; R_4_ = CH_3_; R_5_ = H; R_6_ = *α*-CH_3_	*Fritillaria ussuriensis*	[131]
**303**	Ebeiedinone	R_1_ = *β*-OH; R_2_ = O; R_3_ = H; R_4_ = CH_3_; R_5_ = H; R_6_ = *β*-CH_3_	*F. ebeiensis*	[132]
**304**	Delafrine	R_1_ = R_2_ = *β*-OH; R_3_ = CH_3_; R_4_ = H; R_5_ = H; R_6_ = *β*-CH_3_	*F. delavayi*	[133]
**305**	Delafrinone	R_1_ = *β*-OH; R_2_ = O; R_3_ = CH_3_; R_4_ = H; R_5_ = H; R_6_ = *β*-CH_3_	*F. delavayi*	[133]
**306**	Zhebeinine	R_1_ = *β*-OH; R_2_ = *α*-OH; R_3_ = OH; R_4_ = CH_3_; R_5_ = H; R_6_ = *α*-CH_3_	*F. thunbergii*	[134]
**307**	Puqiedinone	R_1_ = *β*-OH; R_2_ = O; R_3_ = H; R_4_ = CH_3_; R_5_ = H; R_6_ = *α*-CH_3_	*F. puqiensis*	[135]
**308**	Zhebeinone	R_1_ = *β*-OH; R_2_ = O; R_3_ = OH; R_4_ = CH_3_; R_5_ = H; R_6_ = *α*-CH_3_	*F. thunbergii*	[136]
**309**	Dongbeinine	R_1_ = *β*-OH; R_2_ = O; R_3_ = CH_3_; R_4_ = H; R_5_ = H; R_6_ = *β*-CH_3_	*F. thunbergii*	[137]
**310**	Dongbeirine	R_1_ = *β*-OH; R_2_ = O; R_3_ = CH_3_; R_4_ = H; R_5_ = H; R_6_ = *α*-CH_3_	*F. thunbergii*	[137]
**311**	Ebeiedine	R_1_ = R_2_ = *β*-OH; R_3_ = H; R_4_ = CH_3_; R_5_ = H; R_6_ = *β*-CH_3_	*F. ebeiensis*	[138]
**312**	Impericine	R_1_ = R_2_ = *β*-OH; R_3_ = CH_3_; R_4_ = H; R_5_ = H; R_6_ = *β*-CH_3_; △^23,24^	*F. imperialis*	[139]
**313**	Forticine	R_1_ = R_2_ = *β*-OH; R_3_ = CH_3_; R_4_ = H; R_5_ = H; R_6_ = *β*-CH_3_	*F. imperialis*	[139]
**314**	Lichuanine	R_1_ = R_2_ = *β*-OH; R_3_ = CH_3_; R_4_ = H; R_5_ = H; R_6_ = *α*-CH_3_	*F. lichuanensis*	[140]
**315**	Puqiedine	R_1_ = R_2_ = *β*-OH; R_3_ = H; R_4_ = CH_3_; R_5_ = H; R_6_ = *α*-CH_3_	*F. puqiensis*	[141]
**316**	3*α*-Puqiedin-7-ol	R_1_ = R_3_ = *α*-OH; R_2_ = *β*-OH; R_4_ = H; R_5_ = H; R_6_ = *β*-CH_3_	*F. puqiensis*	[141]
**317**	Yibeinone F	R_1_ = O-*β*-d-Glc; R_2_ = *α*-OH; R_3_ = H; R_4_ = CH_3_; R_5_ = *α*-OH; R_6_ = *β*-CH_3_	*F. pallidiflora*	[142]
**318**	Verticine *N*-oxide	R_1_ = *α*-OH; R_2_ = OH; R_3_ = CH_3_	*F. thunbergii*	[143]
**319**	Verticinone *N*-oxide	R_1_ = O; R_2_ = OH; R_3_ = CH_3_	*F. thunbergii*	[143]
**320**	Isoverticine-*β*-*N*-oxide	R_1_ = *β*-OH; R_2_ = OH; R_3_ = CH_3_	*F. wabuensia*	[144]
**321**	Lichuanisinine	R_1_ = *β*-OH; R_2_ = CH_3_; R_3_ = H	*F. lichuanensis*	[140]
**322**	Pingbeimine A	R_1_ = *α*-OH; R_2_ = H	*F. ussuriensis*	[145]
**323**	Pingbeimine B	R_1_ = *α*-OH; R_2_ = OH	*F. ussuriensis*	[146]
**324**	Pingbeimine C	R_1_ = O; R_2_ = H	*F. ussuriensis*	[147]
**325**	Delavine	R_1_ = R_2_ = *β*-OH; R_3_ = H; R_4_ = CH_3_	*F. delavayi*	[148]
**326**	Hupeheninoside	R_1_ = *β*-d-Glc; R_2_ = *β*-OH; R_3_ = H; R_4_ = CH_3_	*F. hupehensis*	[149]
**327**	Delavinone	R_1_ = R_3_ = H; R_2_ = O; R_4_ = CH_3_	*F. delavayi*	[148]
**328**	Hupehenirine	R_1_ = O; R_2_ = *β*-OH; R_3_ = H; R_4_ = CH_3_	*F. hupehensis*	[150]
**329**	Yibeinoside A	R_1_ = *O*-*β*-d-Glc; R_2_ = O; R_3_ = H; R_4_ = CH_3_	*F. pallidiflora*	[151]
**330**	Hupehemonoside	R_1_ = *β*-d-Glc; R_2_ = O; R_3_ = OH; R_4_ = CH_3_	*F. delavayi*	[152]
**331**	Delavine-3-*O*-*β*-d-Glucopyranoside	R_1_ = *O*-*β*-d-Glc; R_2_ = *β*-OH; R_3_ = H; R_4_ = CH_3_	*F. persica*	[153]
**332**	Yubeinine	R_1_ = *α*-OH; R_2_ = O; R_3_ = OH; R_4_ = CH_3_	*F. yuminensis*	[154]
**333**	Yubeiside	R_1_ = O; R_2_ = *β*-*O*-*β*-d-Glc; R_3_ = R_4_ = H	*F. yuminensis*	[154]
**334**	Hupeheninate	R_1_ = Ac; R_2_ = *β*-OH; R_3_ = H; R_4_ = CH_3_	*F. delavayi*	[155]
**335**	Imperialine	R_1_ = *β*-OH; R_2_ = O; R_3_ = OH; R_4_ = CH_3_	*F. pallidiflora*	[156]
**336**	Chuanbeinone	R_1_ = R_3_ = H; R_2_ = O; R_4_ = CH_3_; R_5_ = *β*-CH_3_; R_6_ = *β*-H	*F. delavayi*	[157]
**337**	Hareperminside	R_1_ = d-Glc; R_2_ = *β*-OH; R_3_ = H; R_4_ = CH_3_; R_5_ = *α*-CH_3_; R_6_ = *β*-H	*F. karelinii*	[158]
**338**	Tortifoline	R_1_ = R_4_ = H; R_2_ = *β*-OH; R_3_ = CH_3_; R_5_ = *β*-CH_3_; R_6_ = *β*-H	*F. tortifolia*	[159]
**339**	Siechuansine	R_1_ = H; R_2_ = *α*-OH; R_3_ = OH; R_4_ = CH_3_; R_5_ = *α*-CH_3_; R_6_ = *β*-H	*F. siechuanica*	[160]
**340**	Songbeinone	R_1_ = R_4_ = H; R_2_ = O; R_3_ = CH_3_; R_5_ = *β*-CH_3_; R_6_ = *β*-H	*F. unibracteata*	[161]
**341**	Yibeinoside B	R_1_ = d-Glc; R_2_ = O; R_3_ = H; R_4_ = CH_3_; R_5_ = *β*-CH_3_; R_6_ = *β*-H	*F. pallidiflora*	[151]
**342**	Persicanidine B/Harepermine	R_1_ = R_3_ = H; R_2_ = *β*-OH; R_4_ = CH_3_; R_5_ = *α*-CH_3_; R_6_ = *β*-H	*F. karelinii*; *F. persica*	[158, 162]
**343**	Yibeinone D	R_1_ = d-Glc; R_2_ = O; R_3_ = CH_3_; R_4_ = OH; R_5_ = *β*-CH_3_; R_6_ = *α*-H	*F. pallidiflora*	[156]
**344**	Wanpeinine A	R_1_ = *α*-OH; R_2_ = OH; R_3_ = CH_3_; R_4_ = *β*-CH_3_; R_5_ = *α*-H; R_6_ = R_7_ = *β*-H	*F. anhuiensis*	[163]
**345**	Persicanidine A	R_1_ = *β*-OH; R_2_ = H; R_3_ = CH_3_; R_4_ = *α*-CH_3_; R_5_ = R_7_ = *α*-H; R_6_ = *β*-H	*F. persica*	[164]
**346**	Yibeirine	R_1_ = *β*-OH; R_2_ = OH; R_3_ = CH_3_; R_4_ = *β*-CH_3_; R_5_ = R_7_ = *β*-H; R_6_ = *α*-H	*F. pallidiflora*	[123]
**347**	Yibeinone C	R_1_ = O; R_2_ = OH; R_3_ = CH_3_; R_4_ = *α*-CH_3_; R_5_ = R_7_ = *β*-H; R_6_ = *α*-H	*F. pallidiflora*	[156]
**348**	Yibeinone E	R_1_ = O; R_2_ = CH_3_; R_3_ = H; R_4_ = *α*-CH_3_; R_5_ = R_6_ = *α*-H; R_7_ = H	*F. pallidiflora*	[165]
**349**	Germaline	R_1_ = R_3_ = R_6_ = H; R_2_ = COC(OH)(CH_3_)CH(OAc)CH_3_; R_4_ = *α*-OH; R_5_ = *α*-*O*-2-methylbutyroyl	*Veratrum. lobelianum*	[166]
**350**	Germatetrine	R_1_ = R_3_ = R_6_ = H; R_2_ = COC(OH)(CH_3_)CH(OAc)CH_3_; R_4_ = *α*-OAc; R_5_ = *α*-*O*-2-methylbutyroyl	*V. lobelianum*	[166]
**351**	Stenophylline A	R_1_ = R_3_ = R_6_ = H; R_2_ = Ang; R_4_ = *α*-OH; R_5_ = *α*-O-Ang	*V. stenophyllum*	[167]
**352**	Maackinine	R_1_ = R_3_ = R_6_ = H; R_2_ = Ang; R_4_ = *α*-OAc; R_5_ = *α*-O-Ang	*V. maackii*	[168]
**353**	Verussurinine	R_1_ = R_2_ = R_3_ = H; R_4_ = R_5_ = *α*-OH; R_6_ = 2-methylbutyroyl	*V. nigrum var. ussuriense*	[169]
**354**	Verussurine	R_1_ = R_3_ = R_6_ = H; R_2_ = Ver; R_4_ = *α*-OAc; R_5_ = *α*-OCOCH(CH_3_)CH_2_CH_3_	*V. nigrum var. ussuriense*	[170]
**355**	Verabenzoamine	R_1_ = R_3_ = R_6_ = H; R_2_ = Ver; R_4_ = *α*-OH; R_5_ = *α*-OCOCH(CH_3_)CH_2_CH_3_	*V. nigrum var. ussuriense*	[170]
**356**	Angeloylzygadenine	R_1_ = R_3_ = R_4_ = R_6_ = H; R_2_ = Ang; R_5_ = *α*-OH	*V. viride*	[171]
**357**	Zygadenine	R_1_ = R_2_ = R_3_ = R_4_ = R_6_ = H; R_5_ = *α*-OH	*V. viride*	[171]
**358**	Germine	R_1_ = R_2_ = R_3_ = R_6_ = H; R_4_ = *β*-OH; R_5_ = *α*-OH	*V. viride*	[171]
**359**	15-*O*-Methylbutyroylgermine	R_1_ = R_2_ = R_3_ = R_6_ = H; R_4_ = *β*-OH; R_5_ = *α*-O-Methylbutyroyl	*V. viride*	[171]
**360**	Neojerminalanine	R_1_ = *α*-OAc; R_2_ = R_3_ = R_6_ = H; R_4_ = *α*-OH; R_5_ = *α*-OOCOCH(CH_3_)CH_2_CH_3_	*V. album*	[172]
**361**	15-Angeloylgermine	R_1_ = R_2_ = R_3_ = R_6_ = H; R_4_ = *α*-OH; R_5_ = Ang	*V. taliense*	[173]
**362**		R_1_ = R_3_ = R_6_ = H; R_2_ = Ac; R_4_ = *α*-OAc; R_5_ = Ang	*V. dahuricum*	[174]
**363**		R_1_ = R_3_ = R_5_ = R_6_ = H; R_2_ = Ver; R_4_ = *α*-OH	*V. dahuricum*	[174]
**364**		R_1_ = R_3_ = R_6_ = H; R_2_ = Ac; R_4_ = *α*-OH; R_5_ = Ang	*V. dahuricum*	[174]
**365**		R_1_ = R_3_ = R_6_ = H; R_2_ = Ver; R_4_ = *α*-OH; R_5_ = Ang	*V. dahuricum*	[174]
**366**	15-*O*-(2-Methylbutanoyl)-3-*O*-veratroylprotoverine	R_1_ = R_6_ = H; R_2_ = Ver; R_3_ = R_4_ = *α*-OH; R_5_ = *α*-OCOCH(CH_3_)CH_2_CH_3_	*V. nigrum*	[175]
**367**	Veramadine A	R_1_ = R_3_ = R_5_ = R_6_ = H; R_2_ = Ver; R_4_ = *α*-OH	*V. maackii var. japonicum*	[176]
**368**	Veramadine B	R_1_ = R_2_ = R_3_ = R_4_ = R_5_ = R_6_ = H	*V. maackii var. japonicum*	[176]
**369**	Ebeienine	R_1_ = R_2_ = *β*-OH	*Fritillaria ebeiensis*	[138]
**370**	Ziebeimine	R_1_ = *α*-OH; R_2_ = *β*-OH	*F. ebeiensis*	[132]
**371**	Heilonine		*F. ussuriensis*	[121]
**372**	Pingbeinone		*F. ussuriensis*	[120]
**373**	Ussuriedine	R_1_ = *β*-OH; R_2_ = H	*F. ussuriensis*	[177]
**374**	Ussuriedinone	R_1_ = O; R_2_ = H	*F. ussuriensis*	[177]
**375**	Ussurienine	R_1_ = *β*-OH; R_2_ = CH_3_	*F. ussuriensis*	[177]
**376**	Ussurienone	R_1_ = O; R_2_ = CH_3_	*F. ussuriensis*	[177]
**377**	Taipaienine	R_1_ = H; R_2_ = *β*-H	*F. taipaiensis*	[122]
**378**	Yibeisine	R_1_ = OH; R_2_ = *α*-H	*F. pallidiflora*	[123]
**379**	Neoverataline A	R = H	*Veratrum taliense*	[125]
**380**	Neoverataline B	R = *α*-OH	*V. taliense*	[125]
**381**		R = Ver	*V. nigrum*	[126]
**382**	Frithunbol A		*Fritillaria thunbergii*	[178]
**383**	Frititorine A		*F. tortifolia*	[124]
**384**	Frititorine B	R = d-Glc	*F. tortifolia*	[124]

Veratrenone (**294**), the first alkaloid with a cevanine skeleton from *V. album*, was investigated in 1974 [118]. The structure of isobaimonidine (**301**), the C-6 epimer of baimonidine (**299**), was deduced by chemical transformation [119]. Eleven glycoalkaloids (**302**, **317**, **326**, **329–331, 333, 337, 341**, **343** and **384**) have been isolated from various *Fritillaria* species in cevanine-type alkaloids. Alkaloid pingbeinone (**372**) has a unique structure with a lack of a C-18 methylene unit, and its structure could be unequivocally established by X-ray diffraction of its corresponding hydroiodide salt [120]. Alkaloids **349–368** belong to the rarely occurring class of cholestane alkaloids having a tetrahydrofuran ring incorporated in their structures. Heilonine (**371**), the first example with an aromatic D-ring in the group of cevanine alkaloids, was isolated from *Fritillaria ussuriensis* in the group of Kaneko [121]. Compounds **373–376** are four unique steroidal alkaloids with a seven-membered G-ring formed by a connection between C-18 and C-27. Taipaienine (**377**) [122] and yibeisine (**378**) [123], which are unique in bearing a C-25 hydroxyl moiety as of a cevanine system, have been isolated from *Fritillaria taipaiensis* and *Fritillaria pallidiflora*, respectively. Compounds **318–321** and frititorine B (**384**) [124] are steroidal alkaloid *N*-oxide derivatives. Neoverataline A (**379**) and neoverataline B (**380**), having a novel 3,4-secocevane-4,9-olid-3-oic acid skeleton, were obtained from the genus *Veratrum* [125]. Compound **381** possesses a rare 9-hydroxy moiety within cevanine-type alkaloids [126].

##### Veratramine type

**Fig. 8:**
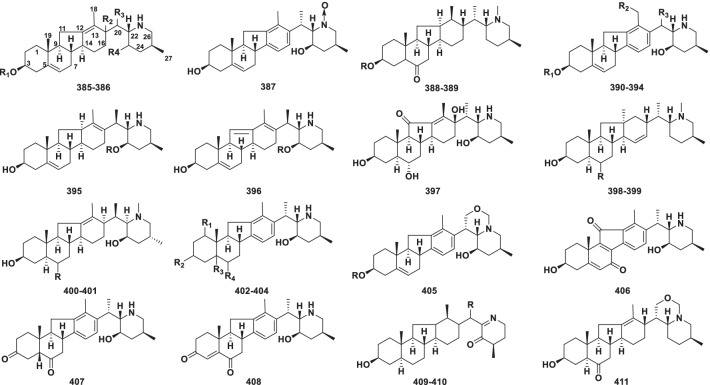
Structures of veratramine type steroidal alkaloids **385–411**

The veratramine type of steroidal alkaloids, in which ring E of cevane has been opened at C-18 (Fig. 8). Compounds **385–411**, a total of 27 veratramine alkaloids, have been found in *Veratrum* and *Fritillaria* (Table 7).

**Table 7:** Structures and sources of veratramine type steroidal alkaloids **385–411**

No	Compounds	Substitution groups and others	Sources	References
**385**	Hosukinidine	R_1_ = R_2_ = R_4_ = H; R_3_ = *β*-CH_3_	*Veratrum grandiflorum*	[180]
**386**	Veratramanol A	R_1_ = X; R_2_ = *β*-H; R_3_ = *α*-CH_3_; R_2_ = *β*-OAc	*Veratrum maackii var. japonicum*	[181]
**387**	Veratramine-*N*-oxide		*V. mentzeanum*	[182]
**388**	Ningpeisine	R = H	*Fritillaria ningguoensis*	[183]
**389**	Ningpeisinoside	R = d-Glc	*F. ningguoensis*	[183]
**390**	20-Isoveratramine	R_1_ = H; R_2_ = H; R_3_ = *β*-CH_3_	*Veratrum patulum*	[184]
**391**	Veratramine-3-yl acetate	R_1_ = Ac; R_2_ = H; R_3_ = *α*-CH_3_	*V. nigrum*	[129]
**392**	Veratramine	R_1_ = H; R_2_ = H; R_3_ = *α*-CH_3_	*V. dahuricum*	[185]
**393**	Veratrosine	R_1_ = d-Glc; R_2_ = H; R_3_ = *α*-CH_3_	*V. dahuricum*	[185]
**394**	Veratravine E	R_1_ = H; R_2_ = OH; R_3_ = *α*-CH_3_	*Veratrum taliense*	[186]
**395**	23-*O*-*β*-d-Glucopyranosyl-20-isoveratramine	R = d-Glc	*V. patulum*	[187]
**396**	(22*S*,23*R*,25*S*)-23-*O*-*β*-d-glucopyranosyl-5,11,13-veratratriene-3b,23-diol	R = d-Glc	*V. patulum*	[187]
**397**	Veramarine		*V. album*	[172]
**398**	Impranine	R = O	*Fritillaria imperialis*	[179]
**399**	Dihydroimpranine	R = *β*-OH	*F. imperialis*	[179]
**400**	Puqienine A	R = *β*-OH	*F. puqiensis*	[188]
**401**	Puqienine B	R = O	*F. puqiensis*	[188]
**402**	Yibeinone B	R_1_ = H; R_2_ = *β*-OH; R_3_ = *α*-H; R_4_ = O	*F. pallidiflora*	[156]
**403**	Veratravine F	R_1_ = *α*-OH; R_2_ = *α*-OH; R_3_ = *β*-H; R_4_ = H	*Veratrum taliense*	[186]
**404**	Veratravine G	R_1_ = *α*-OH; R_2_ = *β*-OH; R_3_ = *β*-H; R_4_ = H	*Veratrum taliense*	[186]
**405**	Veratravine A	R = d-Glc	*Veratrum taliense*	[186]
**406**	Veratravine B		*Veratrum taliense*	[186]
**407**	Veratravine C		*Veratrum taliense*	[186]
**408**	Veratravine D		*Veratrum taliense*	[186]
**409**	△^5^(20*R*,24*R*)23-oxo-24-methylsolacongetidine	R = *β*- CH_3_	*Veratrum grandiflorum*	[189]
**410**	△^5^(20*S*,24*R*)23-oxo-24-methylsolacongetidine	R = *α*-CH_3_	*V. grandiflorum*	[189]
**411**	Zhebeisine		*Fritillaria thunbergii*	[190]

Thirteen alkaloids, **387**, **390–394**, **402–408** containing an aromatic D-ring are unusual in C_27_ steroidal alkaloids, and concurrently **387** is a steroidal alkaloid *N*-oxide derivative. A chemical investigation of the hypogeal parts of *Fritillaria imperialis* furnished two unique bases, impranine (**398**) and dihydroimpranine (**399**), which have a methyl group at C-12. This is the first time the novel “impranane” class derived from the veratramine skeleton has been found in the genus *Fritillaria* [179]. Veratravine A (**405**) and zhebeisine (**411**) contain a new oxazinane ring F forming a rare 6/6/5/6/6/6 fused-ring system.

##### Jervine type

**Fig. 9:**
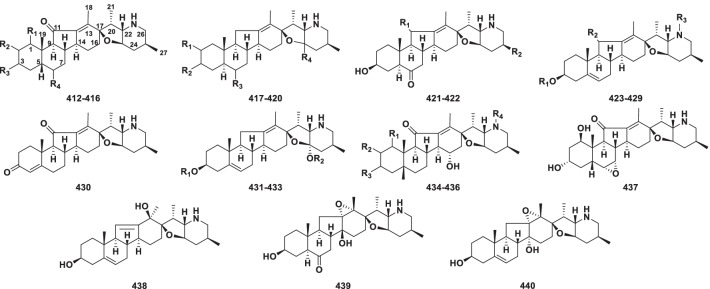
Structures of jervine type steroidal alkaloids **412–440**

The steroidal alkaloids of the jervine subgroup are hexacyclic compounds that have the tetrahydrofuran E ring fused onto a methylpiperidine F ring system forming an ether bridge between carbon atoms C_17_ and C_23_ (Fig. 9) [15]. The jervine type currently consists of 29 new members (**412−440)** from *Veratrum* and *Fritillaria* (Table 8).

**Table 8:** Structures and sources of jervine type steroidal alkaloids **412–440**

No	Compounds	Substitution groups and others	Sources	References
**412**	Verdine	R_1_ = *β*-OH; R_2_ = H; R_3_ = R_4_ = *α*-OH	*Veratrum dahuricum*	[191]
**413**	1-Hydroxy-5,6-dihydrojervine	R_1_ = *α*-OH; R_2_ = R_4_ = H; R_3_ = *β*-OH	*V. album*	[196]
**414**	2*β*-Hydroxyverdine	R_1_ = R_2_ = *β*-OH; R_3_ = R_4_ = *α*-OH	*V. dahuricum*	[197]
**415**	(1*β*,3*α*,5*β*)-1,3-Dihydroxyjervanin-12-en-11- one	R_1_ = *β*-OH; R_2_ = R_4_ = H; R_3_ = *α*-OH	*V. nigrum*	[198]
**416**	Veratraline C	R_1_ = R_3_ = *α*-OH; R_2_ = R_4_ = H	*V. taliense*	[199]
**417**	Kuroyurinidine	R_1_ = *β*-OH; R_2_ = *α*-OH; R_3_ = *β*-OH; R_4_ = *α*-H	*Fritillaria camtschatcensis*	[193]
**418**	23-Isokuroyurinidine	R_1_ = *β*-OH; R_2_ = *α*-OH; R_3_ = *β*-OH; R_4_ = *β*-H	*F. maximowiczii*	[194]
**419**	Frithunbol B	R_1_ = H; R_2_ = *β*-OH; R_3_ = O; R_4_ = *β*-H	*F. thunbergii*	[178]
**420**	Frititorinec	R_1_ = H; R_2_ = *α*-OH; R_3_ = O; R_4_ = *α*-H	*Fritillaria tortifolia*	[124]
**421**	Yibeissine	R_1_ = *β*-OH; R_2_ = *β*-CH_3_	*F. pallidiflora*	[200]
**422**	Tortifolisine	R_1_ = H; R_2_ = *α*-CH_3_	*F. tortifolia*	[201]
**423**	Verapatulin	R_1_ = H; R_2_ = O; R_3_ = COOCH_3_	*Veratrum patulum*	[184]
**424**	*O*-Acetyljervine	R_1_ = Ac; R_2_ = O; R_3_ = H	*V. album*	[202]
**425**	Methyljervine-*N*-3′-propanoate	R_1_ = H; R_2_ = O; R_3_ = (CH_2_)_2_COOCH_3_	*V. album*	[202]
**426**	Neoverapatuline	R_1_ = H; R_2_ = *α*-OH; R_3_ = COOCH_3_	*V. nigrum*	[198]
**427**	Jervine	R_1_ = R_3_ = H; R_2_ = O	*V. dahuricum*	[185]
**428**	Jervine-3-yl formate	R_1_ = COH; R_2_ = O; R_3_ = H	*V. nigrum*	[129]
**429**	Veratraline A	R_1_ = H; R_2_ = O; R_3_ = CH_2_NHAc	*V. taliense*	[199]
**430**	Jervinone		*V. album*	[196]
**431**	23-Methoxycyclopamine	R_1_ = H; R_2_ = CH_3_	*V. nigrum*	[175]
**432**	Cyclopamine	R_1_ = R_2_ = H	*V. californicum*	[4]
**433**	23-methoxycyclopamine 3-*O*-*β*-d-glucopyranoside	R_1_ = d-Glc; R_2_ = CH_3_	*V. maackii*	[203]
**434**	Veraussine A	R_1_ = R_3_ = *α*-OH; R_2_ = *β*-OH; R_4_ = COOEt	*V. nigrum var. ussuriense*	[204]
**435**	Veraussine B	R_1_ = R_3_ = *α*-OH; R_2_ = *β*-OH; R_4_ = COOCH_3_	*V. nigrum var. ussuriense*	[204]
**436**	(1*β*,3*β*,5*β*)-1,3-Dihydroxyjervanin-12(13)-en-11-one	R_1_ = R_3_ = *β*-OH; R_2_ = R_4_ = H	*V. nigrum*	[129]
**437**	6,7-Epoxyverdine		*V. taliense*	[195]
**438**	Jerv-5,11-diene-3*β*,13*β*-diol		*V. nigrum*	[129]
**439**	Yibeinone A		*Fritillaria pallidiflora*	[156]
**440**	Veratraline B		*Veratrum taliense*	[199]

Verdine (**412**) was first separated from the bulbs of *Veratrum dahuricum* in 1980 [191], and its structure was finally elucidated in 1984 by X-ray diffraction [192]. Two new steroidal alkaloids, kuroyurinidine (**417**) [193] and 23-isokuroyurinidine (**418**) [194], bearing C-2*β*, C-3*α*, and C-6*β* hydroxyl groups, were found in the genus *Fritillaria*. Whole plants of *Veratrum taliense* have yielded a novel steroidal alkaloid, 6,7-epoxyverdine (**437**), whose structure with an epoxide functionality at C-5/C-6 was determined by 2D NMR spectroscopic analysis [195]. Yibeinone A (**439**) features a jervine skeleton with a rare 12*α*,13*α*-epoxy ring [156].

##### Spirosolane type

**Fig. 10:**
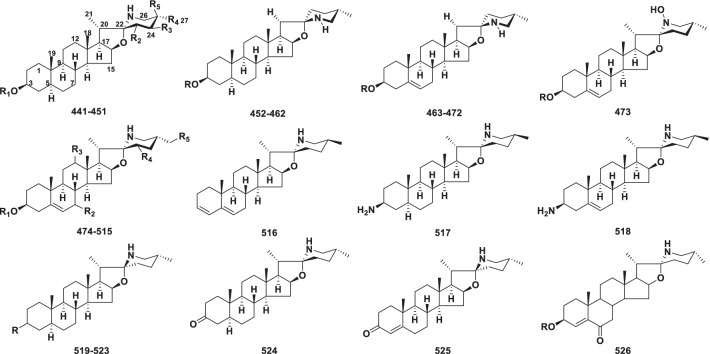
Structures of spirosolane type steroidal alkaloids **441–526**

Spirosolane alkaloids (**441–526**) have a unique 1-oxa-6-azaspiro[4.5] decane ring system in ring E, which can form a spirosolane 22-*α* N type and 22-*β* N type (Fig. 10) [205]. They were reported from *Solanum* and *Lycopersicon* in the Solanaceae family, *Fritillaria meleagris* and *Lilium longiflorum* in the Liliaceae family (Table 9).

**Table 9:** Structures and sources of spirosolane type steroidal alkaloids **441–526**

No	Compounds	Substitution groups and others	Sources	References
**441**	*β*-Soladulcine	R_1_ = Solatriose; R_2_ = R_3_ = R_5_ = H; R_4_ = CH_3_	*Solanum dulcamara*	[210]
**442**	Soladulcidine	R_1_ = R_2_ = R_3_ = R_4_ = R_5_ = H	*S. dulcamara*	[211]
**443**	Soladulcine A	R_1_ = Chacotriose; R_2_ = R_3_ = R_5_ = H; R_4_ = CH_3_	*S. dulcamara*	[212]
**444**	Soladulcine B	R_1_ = Lycotetraose; R_2_ = R_3_ = R_5_ = H; R_4_ = CH_3_	*S. dulcamara*	[212]
**445**	Dihydrosolasuaveoline	R_1_ = l-Rha-(1 → 2)-[D-Glc-(1 → 2)-d-Glc-(1 → 4)]-d-Gal; R_2_ = R_3_ = R_5_ = H; R_4_ = CH_3_	*S. suaveolens*	[213]
**446**	Solalyratine A	R_1_ = d-Xyl-(1 → 3)-d-Gal; R_2_ = R_3_ = R_5_ = H; R_4_ = CH_3_	*S. lyratum*	[214]
**447**	Solalyratine B	R_1_ = [d-Xyl-(1 → 2)-d-Glc-(1 → 4)-d-Gal]; R_2_ = R_3_ = R_5_ = H; R_4_ = CH_3_	*S. lyratum*	[214]
**448**	Esculeoside A	R_1_ = Lycotetraose; R_2_ = OAc; R_3_ = R_4_ = H; R_5_ = CH_2_-*O*-d-Glc	*Lycopersicon esculentum* var. *cerasiforme*	[206]
**449**	Lycotetraose G	R_1_ = Lycotetraose; R_2_ = OAc; R_3_ = *O*-d-Glc; R_4_ = CH_3_; R_5_ = H	*Lycopersicon esculentum* var. *cerasiforme*	[206]
**450**	22-Imido-3-[4ʹ-(6ʺ-deoxy-*α*-l-mannoside)-*β*-d-glucoside]-5-dehydro spirostane	R_1_ = l-Rha-(1 → 4)-d-Glc; R_2_ = R_3_ = R_4_ = H; R_5_ = CH_3_	*Solanum xanthocarpum*	[215]
**451**	Neorickiioside B	R_1_ = Lycotetraose; R_2_ = OH; R_3_ = R_5_ = H; R_4_ = CH_3_	*S. neorickii*	[216]
**452**	*β*_2_-Tomatine	R = d-Xyl-(1 → 3)-d-Glc-(1 → 4)-d-Gal	*Lycopersicon esculentum*	[217]
**453**	Dihydro-*β*-Solamarine	R = Chacotriose	*Solanum dulcamara*	[218]
**454**	Polyanine	R = d-Xyl-(1 → 2)-d-Xyl-(1 → 3)-d-Glc	*S. polyadenium*	[219]
**455**	Sisunine	R = Commertetraose	*S. ajanhuiri*	[220]
**456**	Tomatidine-3-*O*-*β*-d-glucopyranoside	R = d-Glc	*S. arboreum*	[221]
**457**	Tomatidine-3-*O*-*β*-[d-xylopyranosyl-(1 → 6)]-*β*-d-glucopyranoside	R = d-Xyl-(1 → 6)-d-Glc	*S. arboreum*	[221]
**458**	Tomatidine	R = H	*Lycopersicon esculentum*	[222]
**459**	*α*-Tomatine	R = Lycotetraose	L*. esculentum*	[216]
**460**	*β*-Tomatine	R = d-Glc-(1 → 2)-d-Glc-(1 → 4)-d-Gal	*Solanum nigrum*	[223]
**461**	*γ*-Tomatine	R = d-Glc-(1 → 4)-d-Gal	*S. tuberosum*	[223]
**462**	*Δ*-Tomatine	R = d-Gal	*Lycopersicon esculentum*	[223]
**463**	*γ*-Solamarine	R = l-Rha-(1 → 4)-d-Glc	*Solanum dulcamara*	[224]
**464**	*γ*_1_-Solamarine	R = l-Rha-(1 → 2)-d-Glc	*S. dulcamara*	[225]
**465**	*δ*-Solamarine	R = d-Glc-(1 → 3)-d-Gal	*S. dulcamara*	[225]
**466**	22,25-Diepisycophantine	R = [D-Xyl-(1 → 2)-l-Rha-(1 → 4)]-l-Rha-(1 → 2)-d-Glc	*S. sycophanta*	[226]
**467**	Solaculine A	R = [D-Xyl-(1 → 2)-l-Rha-(1 → 4)]-l-Rha-(1 → 2)-d-Glc	*S. aculeastrum*	[227]
**468**	Tomatidenol	R = H	*S. auiculare*	[228]
**469**	(22*S*, 25*S*)-spirosol-5-en-3*β*-yl *O*-*β*-d-glucopyranosyl-(1 → 4)-*O*-[*α*-l-rhamnopyranosyl-(1 → 2)]-*β*-d-glucopyranoside	R = d-Glc-(1 → 4)-[L-Rha-(1 → 2)]-d-Glc	*Fritillaria meleagris*	[229]
**470**	*β*-Solamarine	R = d-Glc-(1 → 4)-[L-Rha-(1 → 2)]-d-Glc	*S. nigrum*	[223]
**471**	*α*-Solamarine	R = Solatriose	*Solanum nigrum*	[223]
**472**	Dehydrotomatine	R = Lycotetraose	*Lycopersicon esculentum*	[223]
**473**	*N*-hydroxysolamargine	R = Chacotriose	*S. robustum*	[230]
**474**	3-*O*-*β*-lycotetraoside of solasodine	R_1_ = Lycotetraose; R_2_ = R_3_ = R_4_ = R_5_ = H	*S. japonense*	[231]
**475**	Spirosolane *β*-d-glucopyranoside deriv	R_1_ = Chacotriose; R_2_ = R_4_ = H; R_3_ = *β*-OH; R_5_ = OH	*S. nigrum*	[232]
**476**	Solaverine I	R_1_ = Chacotriose; R_2_ = R_3_ = R_5_ = H; R_4_ = OH	*S. toxicarium*; *S. verbascifolium*	[233]
**477**	Solaverine II	R_1_ = d-Glc-(1 → 3)-[L-Rha-(1 → 2)]-d-Gal; R_2_ = R_3_ = R_5_ = H; R_4_ = OH	*S. toxicarium*; *S. verbascifolium*	[233]
**478**	Solaverine III	R_1_ = Chacotriose;R_2_ = R_3_ = H; R_4_ = R_5_ = OH	*S. toxicarium*; *S. verbascifolium*	[233]
**479**	Incanumine	R_1_ = [D-Xyl-(1 → 4)-l-Rha-(1 → 4)]-d-Xyl-(1 → 3)-d-Glc; R_2_ = R_3_ = R_4_ = R_5_ = H	*S. incanum*	[234]
**480**	(23*S*)-23-Hydroxyanguivine	R_1_ = d-Xyl-(1 → 3)-[L-Rha-(1 → 2)]-d-Glc; R_2_ = R_3_ = R_5_ = H; R_4_ = OH	*S. uporo*	[233]
**481**	*β*_1_-Solamargine	R_1_ = l-Rha-(1 → 2)-d-Glc; R_2_ = R_3_ = R_4_ = R_5_ = H	*S. robustum*	[235]
**482**	Anguivine	R_1_ = [D-Xyl-(1 → 3)-l-Rha-(1 → 2)]-d-Glc; R_2_ = R_3_ = R_4_ = R_5_ = H	*S. anguivi*	[236]
**483**	Robustine	R_1_ = l-Ara-(1 → 3)-[L-Rha-(1 2)]-[L-Rha-(1 → 4)]-d-Glc; R_2_ = R_3_ = R_4_ = R_5_ = H	*S. robustum*	[235]
**484**	Ravifoline	R_1_ = l-Rha-(1 → 4)-[L-Rha-(1 → 2)]-d-Xyl; R_2_ = R_3_ = R_4_ = R_5_ = H	*S. platanifolium*	[237]
**485**	(3*β*,22*α*,25R)-Spirosol-5-en-3-yl 6-deoxy-*α*-l-mannopyranoside	R_1_ = l-Rha; R_2_ = R_3_ = R_4_ = R_5_ = H	*S. unguiculatum*	[238]
**486**	Robeneoside A	R_1_ = Chacotriose; R_2_ = R_4_ = R_5_ = H; R_3_ = *α*-OH	*S. lycocarpum*	[238]
**487**	3-*O*-*α*-l-rhamnopyranosyl-(1 → 2)-*α*-l-rham-nopyranosyl-(1 → 4)-*β*-d-galactopyranosyl solasodine	R_1_ = l-Rha-(1 → 4)-[L-Rha-(1 → 2)]-d-Gal; R_2_ = R_3_ = R_4_ = R_5_ = H	*S. unguiculatum*	[238]
**488**	Sycophantine	R_1_ = [D-Xyl-(1 → 2)-l-Rha-(1 → 4)]-l-Rha-(1 → 2)-d-Glc; R_2_ = R_3_ = R_4_ = R_5_ = H	*S. coccineum*	[239]
**489**	Solanelagnin	R_1_ = l-Rha-(1 → 4)-[L-Rha-(1 → 3)]-d-Glc; R_2_ = R_3_ = R_4_ = R_5_ = H	*S. elaeagnifolium*	[240]
**490**	12-Hydroxysolasonine	R_1_ = Solatriose; R_2_ = R_4_ = R_5_ = H; R_3_ = *β*-OH	*S. uporo*	[241]
**491**	Isoanguivine	R_1_ = d-Xyl-(1 → 3)-[L-Rha-(1 → 2)]-d-Gal; R_2_ = R_3_ = R_4_ = R_5_ = H	*S. uporo*	[241]
**492**	Solashabanine	R_1_ = [D-Glc-(1 → 6)-d-Glc-(1 → 3)]-l-Rha-(1 → 2)-d-Gal; R_2_ = R_3_ = R_4_ = R_5_ = H	*S. suaveolens*	[213]
**493**	(23*S*)-23-Hydroxyisoanguivine	R_1_ = d-Xyl-(1 → 3)-[L-Rha-(1 → 2)]-d-Gal; R_2_ = R_3_ = R_5_ = H; R_4_ = OH	*S. uporo*	[241]
**494**	Solasuaveoline	R_1_ = l-Rha-(1 → 2)-[D-Glc-(1 → 2)-d-Glc-(1 → 4)-d-Gal; R_2_ = R_3_ = R_4_ = R_5_ = H	*S. suaveolens*	[213]
**495**	(25*R*)-3*β*-[*O*-*α*-l-Rhamnopyranosyl-(1 → 2)-[*O*-*β*-d-glucopyranosyl-(1 → 4)-*O*-*α*-l-rhamnopyranosyl-(1 → 4)]-*β*-d-glucopyranosyl]-22*α*-spirosol-5-ene	R_1_ = [D-Glc-(1 → 4)-l-Rha-(1 → 4)]vRha-(1 → 2)-d-Glc; R_2_ = R_3_ = R_4_ = R_5_ = H	*S. aculeastrum*	[242]
**496**	Arudoine	R_1_ = d-Xyl-(1 → 3)-[L-Rha-(1 → 2)]-[L-Rha-(1 → 4)]-d-Glc; R_2_ = R_3_ = R_4_ = R_5_ = H	*S. arundo*	[243]
**497**	Robeneoside B	R_1_ = Solatriose; R_2_ = R_4_ = R_5_ = H; R_3_ = *α*-OH	*S. lycocarpum*	[244]
**498**	27-Hydroxysolamargine	R_1_ = Chacotriose; R_2_ = R_3_ = R_4_ = H; R_5_ = OH	*S. asperum*	[244]
**499**	12-Hydroxysolamargine	R_1_ = Chacotriose; R_2_ = R_4_ = R_5_ = H; R_3_ = *β*-OH	*S. lycocarpum*	[245]
**500**	*α*-Solamargine	R_1_ = Chacotriose; R_2_ = R_3_ = R_4_ = R_5_ = H	*S. macrocarpon; S. aethiopicum*	[246]
**501**	*α*-Solasonine	R_1_ = Solatriose; R_2_ = R_3_ = R_4_ = R_5_ = H	*S. macrocarpon; S. aethiopicum*	[246]
**502**	(22*R*,25*R*)-spirosol-5-en-3*β*-yl	R_1_ = d-Glc-(1 → 4)-[L-Rha-(1 → 2)]-d-Glc; R_2_ = R_3_ = R_4_ = R_5_ = H	*Lilium longiflorum*	[247]
**503**	*O*-L-rhamnopyranosyl-(1 → 2)-[6-O-acetyl-*β*-d-glucopyranosyl-(1 → 4)]-*β*-d-glucopyranoside	R_1_ = 6-Ac-d-Glc-(1 → 4)-[L-Rha-(1 → 2)]-d-Glc; R_2_ = R_3_ = R_4_ = R_5_ = H	L *longiflorum*	[247]
**504**	(22*R*,25*R*)-16*β*-H-22*α*-N-spirosol-3*β*-ol-5-ene-3-*O*-*α*-L-rhamnopyranosyl-(1 → 2)-[*α*-L-rhamnopyranosyl-(1 → 4)]-*β*-d-glucopyranoside	R_1_ = Chacotriose; R_2_ = R_3_ = R_4_ = R_5_ = H; C_16_ = *R* configuration	*Solanum surattense*	[207]
**505**	*γ*-Solamargine	R_1_ = d-Glc; R_2_ = R_3_ = R_4_ = R_5_ = H	*S. nigrum*	[248]
**506**	*β*_1_-Solasonine	R_1_ = l-Rha-(1 → 2)-d-Gal; R_2_ = R_3_ = R_4_ = R_5_ = H	*S. nigrum*	[248]
**507**	Solanigroside P	R_1_ = l-Rha-(1 → 4)-d-Glc; R_2_ = R_4_ = R_5_ = H; R_3_ = *α*-OH	*S. nigrum*	[248]
**508**	Khasianine	R_1_ = l-Rha-(1 → 4)-d-Glc; R_2_ = R_3_ = R_4_ = R_5_ = H	*S. nigrum*	[249]
**509**	*β*_2_-Solasonine	R_1_ = d-Glc-(1 → 4)-d-Gal; R_2_ = R_3_ = R_4_ = R_5_ = H	*S. nigrum*	[250]
**510**	7*α*-Hydroxy-khasianine	R_1_ = l-Rha-(1 → 4)-d-Glc; R_2_ = *α*-OH; R_3_ = R_4_ = R_5_ = H	*S. nigrum*	[250]
**511**	7*α*-Hydroxy-solamargine	R_1_ = Chacotriose; R_2_ = *α*-OH; R_3_ = R_4_ = R_5_ = H	*S. nigrum*	[250]
**512**	7*α*-Hydroxy-solasonine	R_1_ = Solatriose; R_2_ = *α*-OH; R_3_ = R_4_ = R_5_ = H	*S. nigrum*	[250]
**513**	Solasodine	R_1_ = R_2_ = R_3_ = R_4_ = R_5_ = H	*Lycopersicon esculentum; Solanum nigrum; S.dulcamara*	[223]
**514**	*γ*-Solasonine	R_1_ = d-Gal; R_2_ = R_3_ = R_4_ = R_5_ = H	*S. nigrum*	[223]
**515**	(25*R*)-22*α*N-spirosol-5(6)-en-3*β*-ol-7-oxo-3-*O*-*α*-Lrhamnopyranosyl-(1 → 2)-[*α*-l-rhamnopyranosyl-(1 → 4)]-*β*-d-glucopyranoside	R_1_ = Chacotriose; R_2_ = O; R_3_ = R_4_ = R_5_ = H	*S. nigrum*	[251]
**516**	Solasodiene		*S. torvum*	[252]
**517**	(22*R*,25*R*)3*β*-amino-5*α*-spirosolane		*S. triste*	[208]
**518**	(22*R*,25*R*)3*β*-amino-5-spirosolene		*S. triste*	[208]
**519**	(22*S*,25*S*)-3*β*-aminospirosol-5-ene	R = *β*-NH_2_; △^5,6^	*S. arboreum*	[209]
**520**	Soladunalinidine	R = *β*-NH_2_	*S. arboreum*	[209]
**521**	3-*Epi-*soladunalinidine	R = *α*-NH_2_	*S. arboreum*	[209]
**522**	Caavuranamide	R = *β*-NHCHO	*S. caavurana*	[253]
**523**	5-Tomatidan-3-one	R = O	*S. caavurana*	[253]
**524**	5*β*-solasodan-3-one		*S. aviccculare*	[254]
**525**	4-Tomatiden-3-one		*S. caavurana*	[253]
**526**	(25*R*)-22*αN*-spirosol-4(5)-en-3*β*-ol-6-oxo-3-*O*-*α*-Lrhamnopyranosyl-(1 → 2)-[*α*-l-rhamnopyranosyl-(1 → 4)]-*β*-d-glucopyranoside	R = Chacotriose	*S. nigrum*	[251]

Spirosolane alkaloids generally occur as glycosides. Compounds **441– 451** have no double bond between C-5 and C-6, and the nitrogen atom in the F ring is always in the *α*-orientation (22-*α* N). Ring F can contain other moieties, such as hydroxyl or acetyl groups. Most glycosidic units are attached to the aglycone at the hydroxyl group at C-3, but some of them may be attached at other locations, such as C-6, C-7, C-23, C-25, and C-27. For example, esculeoside A (**448**) and lycotetraose G (**449**) have one glucose linked to ring-F at C-25 and C-23, respectively [206]. Compounds **474–515** are the largest members in spirosolane alkaloids, with 22-*β* N and double bonds between C-5 and C-6. Almost all spirosolane alkaloids at C-16 are in the *β*-orientation, however, **504** is an exception since it possesses a 16 *β*-H in its E ring [207]. There are many substitutions and changes in these compounds, such as **475**, **478** and **498** have a hydroxy group on C-27 in the F ring, and **486**, **497**, **499** and **507** have a hydroxy group on C-12 in the C ring. Five rare C-3 amino spirosolane alkaloids, **517–521** [208, 209] were isolated from aerial parts of the genus *Solanum.*

##### Solanidine type

**Fig. 11:**
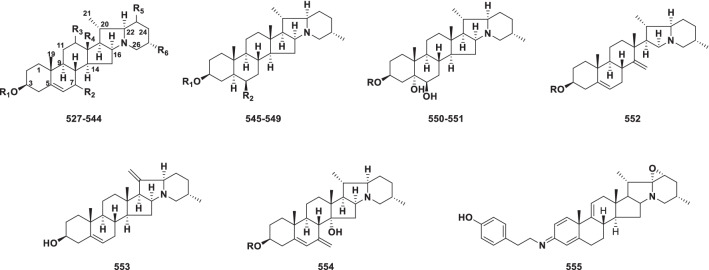
Structures of solanidine type steroidal alkaloids **527–555**

In the solanidine type, the side-chain of a C_27_ steroid has been converted into an indolizidine ring (Fig. 11). Solanidine alkaloids (**527–555**) currently include 29 novel members from *Veratrum*, *Fritillaria* and *Solanum* (Table 10).

**Table 10:** Structures and sources of solanidine type steroidal alkaloids **527–555**

No	Compounds	Substitution groups and others	Sources	References
**527**	*α*-Chaconine	R_1_ = Chacotriose; R_2_ = R_3_ = R_5_ = H; R_4_ = R_6_ = CH_3_	*Solanum chacoense*	[224]
**528**	*α*-Solanine	R_1_ = Solatriose; R_2_ = R_3_ = R_5_ = H; R_4_ = R_6_ = CH_3_	*S. tuberosum*; *S. nigrum*	[255]
**529**	Leptine I	R_1_ = Chacotriose; R_2_ = R_3_ = H; R_4_ = R_6_ = CH_3_; R_5_ = OAc	*S. chacoense*	[224]
**530**	Leptinine I	R_1_ = Chacotriose; R_2_ = R_3_ = H; R_4_ = R_6_ = CH_3_; R_5_ = OH	*S. orbignianum*	[258]
**531**	Leptine II	R_1_ = Solatriose; R_2_ = R_3_ = H; R_4_ = R_6_ = CH_3_; R_5_ = OAc	*S. chacoense*	[224]
**532**	Leptinine II	R_1_ = Solatriose; R_2_ = R_3_ = H; R_4_ = R_6_ = CH_3_; R_5_ = OH	*S. orbignianum*	[258]
**533**	Dehydrodemissine	R_1_ = Lycotetraose; R_2_ = R_3_ = R_5_ = H; R_4_ = R_6_ = CH_3_	*S. commersonii*	[259]
**534**	Dehydrocommersonine	R_1_ = Commertetraose; R_2_ = R_3_ = R_5_ = H; R_4_ = R_6_ = CH_3_	*S. chacoense*	[224]
**535**	Solanidine	R_1_ = R_2_ = R_3_ = R_5_ = H; R_4_ = R_6_ = CH_3_	*S. tuberosum*	[260]
**536**	Epirubijervin	R_1_ = R_2_ = R_5_ = H; R_3_ = *β*-OH; R_4_ = R_6_ = CH_3_	*Veratrum grandiflorum*	[180]
**537**	Camtschatcanidine	R_1_ = R_2_ = R_3_ = R_5_ = H; R_4_ = CH_3_; R_6_ = CH_2_OH	*Fritillaria camtschatcensis*	[261]
**538**	Oligoglycoside	R_1_ = [l-Rha-(1 → 2)][D-Glc-(1 → 4)]-d-Glc; R_2_ = R_3_ = R_5_ = H; R_4_ = R_6_ = CH_3_	*F. thunbergii*	[262]
**539**	(22*S*,25*S*)-solanid-5-en-3*β*-ol	R_1_ = R_2_ = R_3_ = R_5_ = H; R_4_ = R_6_ = CH_3_	*Fritillaria anhuiensis*	[256]
**540**	(3*β*,7*β*)-7-Hydroxysolanid-5-en-3-yl 6-deoxy-*α*-l-mannopyranosyl-(1 → 2)-[6-deoxy-*α*-l-mannopyranosyl-(1 → 4)]-*β*-d-glucopyranoside	R_1_ = 6-deoxy-l-Man-(1 → 2)-[6-deoxy-l-Man-(1 → 4)]-d-Glu; R_2_ = *β*-OH; R_3_ = R_5_ = H; R_4_ = R_6_ = CH_3_	*Solanum tuberosum*	[257]
**541**	(3*β*)-7-Oxosolanid-5-en-3-yl 6-deoxy-*α*-l-mannopyranosyl-(1 → 2)-[6-deoxy-*α*-l-mannopyranosyl-(1 → 4)]-*β*-d-glucopyranoside	R_1_ = 6-deoxy-l-Man-(1 → 2)-[6-deoxy-l-Man-(1 → 4)]-d-Glc; R_2_ = O; R_3_ = R_5_ = H; R_4_ = R_6_ = CH_3_	*S. tuberosum*	[257]
**542**	(3*β*)-7-Oxosolanid-5-en-3-yl 6-Deoxy-*α*-l-mannopyranosyl-(1 → 2)-[*β*-d-glucopyranosyl-(1 → 3)]-*β*-d-galactopyranoside	R_1_ = 6-deoxy-l-Man-(1 → 2)-[D-Glc-(1 → 3)]-d-Gal; R_2_ = O; R_3_ = R_5_ = H; R_4_ = R_6_ = CH_3_	*S. tuberosum*	[257]
**543**	Isorubijervine	R_1_ = R_2_ = R_3_ = R_5_ = H; R_4_ = CH_2_OH; R_6_ = CH_3_	*Veratrum viride*	[263]
**544**	Rubijervine	R_1_ = R_2_ = R_5_ = H; R_3_ = *β*-OH; R_4_ = R_6_ = CH_3_	*V. taliense*	[263]
**545**	Demissine	R_1_ = Lycotetraose; R_2_ = H	*Solanum chacoense*; *S. commersonii*	[264]
**546**	Commersonine	R_1_ = Commertetraose; R_2_ = H	*S. chacoense*; *S. commersonii*	[264]
**547**	Demissidine	R_1_ = R_2_ = H	*S. tuberosum*	[265]
**548**	Dihydro-*β*_1_-Chaconine	R_1_ = l-Rha-(1 → 2)-d-Glc; R_2_ = H	*S. juzepczukii*; *S. curtilobum*	[266]
**549**	Dihydrosolanine	R_1_ = Solatriose; R_2_ = H	*S. juzepczukii*; *S. curtilobum*	[266]
**550**	(22*R*,25*S*)-solanid-5-enine-3*β*,5*α*,6*β*-triol	R = H	*Fritillaria delavayi*	[133]
**551**	(3*β*,5*α*,6*β*)-5,6-Dihydroxysolanidan-3-yl 6-Deoxy-*α*-l-mannopyranosyl-(1 → 2)-[6-deoxy-*α*-l-mannopyranosyl-(1 → 4)]-*β*-d-glucopyranoside	R = 6-deoxy-l-Man-(1 → 2)-[6-deoxy-l-Man-(1 → 4)]-d-Glc	*Solanum tuberosum*	[257]
**552**	15,16-Seco-22*α*H,25*β*H-solanida-5,14-dien-3*β*-ol-*O*-*β*-d-glucopyranosyl-(1 → 4)-*β*-d-xylopyranoside	R = d-Glc-(1 → 4)]-d-Xyl	*Fritillaria maximowiczii*	[194]
**553**	(22*S*,25*S*)-Solanid-5,20(21)-dien-3*β*-ol		*F. anhuiensis*	[256]
**554**	(3*β*)-14-Hydroxysolanid-5-en-3-yl 4-*O*-(6-Deoxy-*α*-l-mannopyranosyl)-*β*-d-glucopyranoside	R = 4-*O*-(6-deoxy-l-Man)-d-Glc	*Solanum tuberosum*	[257]
**555**	(*E*)-*N*-[80(4-hydroxyphenyl)ethyl]-22*α*,23*α*-epoxy-solanida-1,4,9-trien-3-imine		*S. campaniform*	[267]

*α*-Solanine (**528)** was found mainly in the tuber of potato (*Solanum tuberosum* L.) and in the whole plant of the nightshade (*Solanum nigrum* Linn.) of the Solanaceae family [255]. The bulbs of *F. delavayi* yielded (22*R*,25*S*)-solanid-5-enine-3*β*,5*α*,6*β*-triol (**550)** [133], the first example with a glycol moiety at the A- and B-rings in the group of solanidine alkaloids. An investigation of *V. dahuricum* furnished unusual glycoalkaloid **552** [194], which represents the first member of a new class with a 15,16-secosolanida-5,14-diene skeleton. The two novel compounds, **553** [256] and **554** [257], bearing a methylene substituent at C-20 and C-7, respectively, were structurally elucidated by extensive 2D NMR analysis.

##### Verazine type

**Fig. 12:**
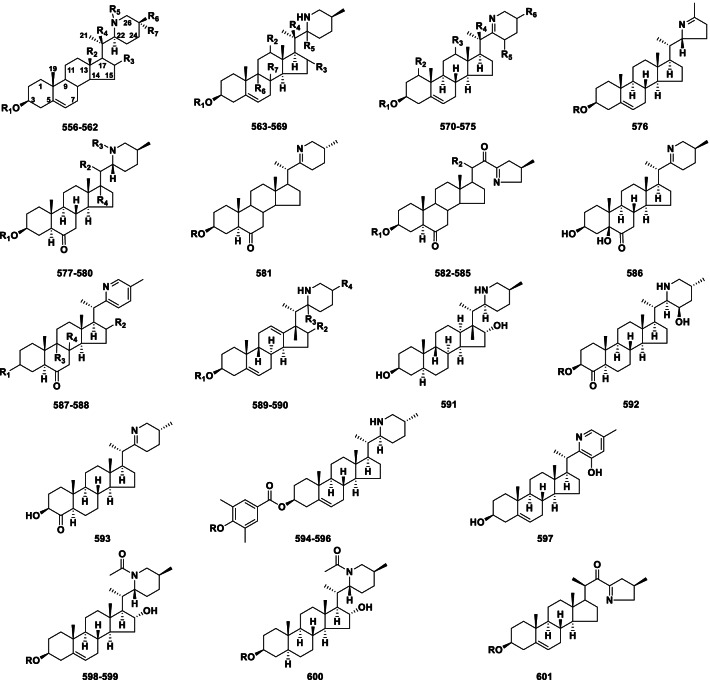
Structures of verazine type steroidal alkaloids **556–601**

Members of the verazine type, having a 22/23,26-epiminocholestane skeleton, are characterized by the absence of ring E and the presence of a piperidine ring D and consist of 46 new members (**556–601)** (Fig. 12). They were obtained from *Veratrum*, *Fritillaria*, *Allium victorialis* and *Zygadenus sibiricus* in the Liliaceae family, and only one *Solanum* species, *Solanum Hypomalacophyllum* (Table 11)*.*

**Table 11:** Structures and sources of verazine type steroidal alkaloids **556–601**

No	Compounds	Substitution groups and others	Sources	References
**556**	Hapepunine	R_1_ = R_4_ = R_5_ = R_7_ = H; R_2_ = R_6_ = CH_3_; R_3_ = *β*-OH	*Fritillaria camtschatcensis*	[272]
**557**	Anrakorinine	R_1_ = R_4_ = R_5_ = R_7_ = H; R_2_ = CH_2_OH; R_3_ = *β*-OH; R_6_ = CH_3_	*F. camtschatcensis*	[272]
**558**	Hapepunine 3-*O*-*α*-l-rhamnopyranosyl-(1 → 2)-*β*-d-glucopyranoside	R_1_ = d-Glc(2 → 1)-l-Rha; R_2_ = R_6_ = CH_3_; R_3_ = *β*-OH; R_4_ = R_5_ = R_7_ = H	*F. thunbergii*	[262]
**559**	Pingbeidinoside	R_1_ = H; R_2_ = R_5_ = R_7_ = CH_3_; R_3_ = *α*-OH; R_4_ = OH; R_6_ = *O*-d-Glc	*F. ussuriensis*	[273]
**560**	Pingbeinine	R_1_ = R_4_ = H; R_2_ = R_5_ = CH_3_; R_3_ = *β*-OH; R_6_ = OH; R_7_ = CH_3_	*F. ussuriensis*	[274]
**561**	Pingbeininoside	R_1_ = d-Glc; R_2_ = R_5_ = CH_3_; R_3_ = *β*-OH; R_4_ = H; R_6_ = OH; R_7_ = CH_3_	*F. ussuriensis*	[274]
**562**	Hapepunine 3-*O*-*β*-cellobioside	R_1_ = d-Glc(4 → 1)-d-Glc; R_2_ = R_6_ = CH_3_; R_3_ = *β*-OH; R_4_ = R_5_ = R_7_ = H	*F. maximowiczii*	[193]
**563**	Muldamine	R_1_ = R_2_ = R_4_ = H; R_3_ = *α*-OAc; R_5_ = R_6_ = *α*-H; R_7_ = *β*-H	*Veratrum californicum*	[275]
**564**	Stenophylline B	R_1_ = R_2_ = R_3_ = H; R_4_ = OH; R_5_ = R_6_ = *β*-H; R_7_ = *α*-H	*V. stenophyllum*	[268]
**565**	Vertaline B	R_1_ = R_2_ = H; R_3_ = *β*-OH; R_4_ = OH; R_5_ = R_7_ = *β*-H; R_6_ = *α*-H	*V. taliense*	[276]
**566**	Veramiline-3-*O*-*β*-d-glucopyranoside	R_1_ = d-Glc; R_2_ = R_3_ = R_4_ = H; R_5_ = R_6_ = *β*-H; R_7_ = *α*-H	*V. taliense*	[277]
**567**	Stenophylline-*β*-3-*O*-*β*-d-glucopyranoside	R_1_ = d-Glc; R_2_ = R_3_ = H; R_4_ = OH; R_5_ = R_6_ = *β*-H; R_7_ = *α*-H	*V. taliense*	[277]
**568**	Veramivirine	R_1_ = R_3_ = R_4_ = H; R_2_ = *β*-OH; R_5_ = R_7_ = *β*-H; R_6_ = *α*-H	*V. viride*	[278]
**569**	Oblonginine	R_1_ = R_2_ = R_4_ = H; R_3_ = *β*-OH; R_5_ = R_7_ = *β*-H; R_6_ = *α*-H	*V. oblongum*	[279]
**570**	Verazinine	R_1_ = d-Glc; R_2_ = R_3_ = R_4_ = R_5_ = H; R_6_ = *β*-CH_3_	*Zygadenus sibiricus*	[280]
**571**	Veranigrine	R_1_ = R_3_ = R_4_ = R_5_ = H; R_2_ = *β*-OH; R_6_ = *β*-CH_3_	*Veratrum nigrum*	[281]
**572**	Veramitaline	R_1_ = R_2_ = R_4_ = R_5_ = H; R_3_ = *α*-OH; R_6_ = *β*-CH_3_	*V. nigrum*	[281]
**573**	(20*R*,25*R*)-12*β*-*O*-acetyl-20*β*-hydroxyisoverazine	R_1_ = R_2_ = R_5_ = H; R_3_ = *β*-OAc; R_4_ = OH; R_6_ = *α*-CH_3_	*V. grandiflorum*	[282]
**574**	(20*R*,25*R*)-12*β*-*O*-acetyl-20*β*-hydroxyisoverazine-3-*O*-*β*-d-glucopyranoside	R_1_ = d-Glc; R_2_ = R_5_ = H; R_3_ = *β*-OAc; R_4_ = OH; R_6_ = *α*-CH_3_	*V. grandiflorum*	[282]
**575**	(20*R*,25*R*)-isoveralodinine	R_1_ = d-Glc; R_2_ = R_4_ = H; R_3_ = *β*-OAc; R_5_ = O; R_6_ = *α*-CH_3_	*V. grandiflorum*	[282]
**576**	Rhamnoveracintine	R = l-Rha	*V. album*	[269]
**577**	Puqietinone	R_1_ = H; R_2_ = *α*-CH_3_; R_3_ = CH_3_; R_4_ = *α*-H	*Fritillaria puqiensis*	[188]
**578**	Yibeinoside C	R_1_ = d-Glc(1 → 4)-d-Gal; R_2_ = *β*-CH_3_; R_3_ = H; R_4_ = *β*-H	*F. pallidiflora*	[283]
**579**	*N*-Demethylpuqietinone	R_1_ = R_3_ = H; R_2_ = *α*-CH_3_; R_4_ = *α*-H	*F. puqiensis*	[188]
**580**	Puqietinonoside	R_1_ = d-Glc; R_2_ = *α*-CH_3_; R_3_ = CH_3_; R_4_ = *α*-H	*F. puqiensis*	[188]
**581**	(25*R*)-22,26-Epimino-3*β*-hydroxy-5*α*-cholest-22(*N*)-en-6-one 3-*O*-*β*-d-glucopyranoside	R = d-Glc	*F. persica*	[284]
**582**	(25*R*)-23,26-Epimino-3*β*-hydroxy-5*α*-cholest-23(*N*)-en-6,22-dione	R_1_ = H; R_2_ = *α*-CH_3_	*F. persica*	[284]
**583**	(25*R*)-23,26-Epimino-3b-hydroxy-5*α*-cholest-23(*N*)-en-6,22-dione 3-*O*-*β*-d-glucopyranoside	R_1_ = d-Glc; R_2_ = *α*-CH_3_	*F. persica*	[284]
**584**	(20*R*,25*R*)-23,26-Epimino-3b-hydroxy-5*α*-cholest-23(*N*)-en-6,22-dione	R_1_ = H; R_2_ = *β*-CH_3_	*F. persica*	[284]
**585**	(20*R*,25*R*)-23,26-Epimino-3b-hydroxy-5a-cholest-23(*N*)-en-6,22-dione 3-*O*-*β*-d-glucopyranoside	R_1_ = d-Glc; R_2_ = *β*-CH_3_	*F. persica*	[284]
**586**	Ebeietinone		*F. ebeiensis*	[270]
**587**	Verdinine	R_1_ = *β*-OAc; R_2_ = *β*-OH; R_3_ = *β*-H; R_4_ = *α*-H	*Veratrum lobelianum*	[271]
**588**	Fetisinine	R_1_ = *α*-OH; R_2_ = H; R_3_ = *α*-H; R_4_ = *β*-H	*Fritillaria imperialis*	[179]
**589**	Diacetylveralkamine	R_1_ = Ac; R_2_ = *α*-OAc; R_3_ = *α*-H; R_4_ = *β*-CH_3_	*Veratrum lobelianum*	[285]
**590**	veralinine 3-*O*-*α*-l-rhamnopyranosyl-(1 → 2)-*β*-d-glucopyranoside	R_1_ = l-Rha-(1 → 2)-d-Glc; R_2_ = H; R_3_ = *β*-H; R_4_ = *α*-CH_3_	*V. grandiflorum*	[189]
**591**	Tetrahydroveralkamine		*V. lobelianum*	[285]
**592**	Deacetoxysolaphyllidine 3-*O*-*β*-d-glucopyranoside		*Solanum hypomalacophyllum*	[286]
**593**	4-Keto-5,6-dihydro-(20*S*)-verazine		*S. hypomalacophyllum*	[286]
**594**	Allumine A	R = H	*Allium victorialis*	[287]
**595**	Allumine B	R = d-Glc	*A. victorialis*	[287]
**596**	Allumine C	R = d-Glc-CH_2_OCO(CH_2_)_10_CH_3_	*A. victorialis*	[288]
**597**	Isoecliptalbine		*Veratrum maackii*	[203]
**598**	Spiraloside A	R = l-Rha-(1 → 4)-d-Glc	*Solanum spirale*	[289]
**599**	Spiraloside B	R = d-Glc	*Solanum spirale*	[289]
**600**	Spiraloside C	R = d-Glc	*Solanum spirale*	[289]
**601**	Tomatillidine 3-*O*-*β*-d-glucopyranoside Veratrum dahuricum	R = X	*V. dahuricum*	[197]

The alkaloidal fraction of *Veratrum stenophyllum* gave a new 3*β*,20*β*-dihydroxy-△^5^–22,26-epiminocholestane alkaloid, Stenophylline B (**564**). Its structure was established on the basis of spectroscopic comparisons with known verazine-type alkaloids [268]. Rhamnoveracintine (**576**), having a five-membered heterocyclic ring and l-rhamnose as structural features, is the first example of a C_26_ steroidal alkaloid from the aerial parts of a *Veratrum* species [269]. Ebeietinone (**586**), the first example of a verazine type alkaloid with a 5*β*-hydroxyl group, was structurally assigned based on MS and NMR and confirmed by X-ray crystallography [270]. Verdinine (**587**) [271], fetisinine (**588**) [179] and isoecliptalbine (**597**) [203], exhibiting a pyridine ring as a structural feature, were pyridyl-pregnane-type steroidal alkaloids, and their structural assignment was performed by extensive spectroscopic techniques and some chemical transformations.

##### Others

**Fig. 13:**
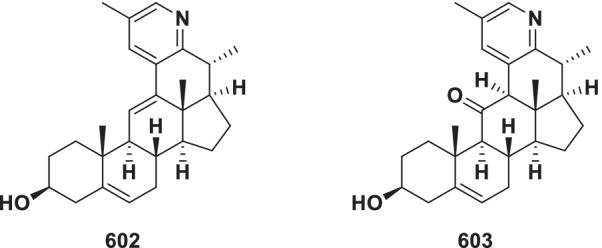
Structures of others cholestane steroidal alkaloids **602–603**

Two distinctive alkaloids, veragranine A (**602**) and veragranine B (**603**), featuring a 6/6/6/5/6/6 polycyclic structure (Fig. 13), in which a previously unidentified linkage of C-12/23 generates a rigid skeleton, resulting in a new subtype of cholestane steroidal alkaloid, were isolated from *Veratrum grandiflorum* [290].

#### Miscellaneous monomeric steroidal alkaloids

##### Samandarines

**Fig. 14:**
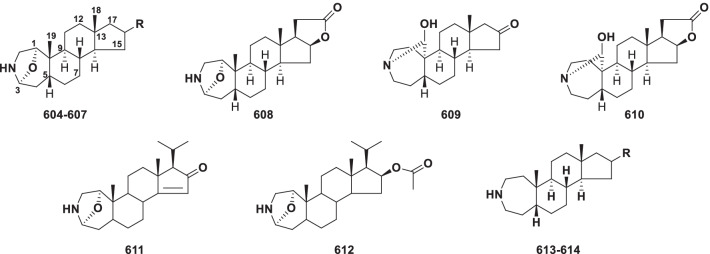
Structures of samandarines **604–614**

Approximately 11 samandarines (**604–614**) are a unique class of steroidal alkaloids isolated and characterized from terrestrial salamanders of the genus *Salamandra* (Table 12). They differ from other types since they are built by a seven-membered A-ring with nitrogen at position 3. Therefore, they belong to the uncommon group of 3-aza-A-homo-5*α*,10*α*-androstans, an androstane with an N-enlarged A-ring (Fig. 14) [291].

**Table 12:** Structures and sources of samandarines **604–614**

No	Compounds	Substitution groups and others	Sources	References
**604**	Samandarine	R = *β*-OH	*Salamandra maculosa*	[293]
**605**	Samandarone	R = O	*S. maculosa*	[294]
**606**	*O*-acetylsamandarine	R = *β*-OAc	*S. maculosa*	[295]
**607**	*O*-(*S*)-3-hydroxybutanoylsamandarine	R = *β*-OCOCH_2_CH(*α*-OH)CH_3_	*S. salamandra*	[296]
**608**	Samandaridine		*S. maculosa*	[294]
**609**	Cycloneosamandione		*S. maculosa*	[292]
**610**	Cycloneosamandaridin		*S. maculosa*	[297]
**611**	Samandenone		*S. maculosa*	[298]
**612**	Samandinine		*S. maculosa*	[299]
**613**	Samanine	R = *β*-OH	*S. maculosa*	[300]
**614**	Samanone	R = O	*S. salamandra*	[296]

Samandarines can be further grouped according to their constitution. The first group consists of molecules with an oxazolidine system present, including **604–607**, **608** and **610–612**. Members of the second group, e.g. cycloneosamandion (**609**), lack an intact oxazolidine system, whereas they share a carbinolamine function [292]. A third group consists of samandarines in which both the oxazolidine system and the carbinolamine group are missing, and only samanine (**613**) and samanone (**614**) were described from this group.

##### Batrachotoxins

**Fig. 15:**
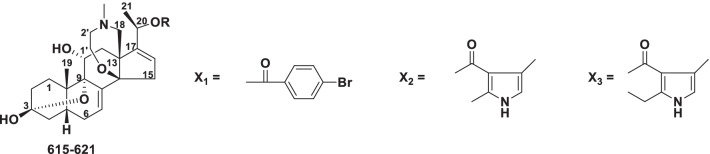
Structures of batrachotoxins **615–621**

Only 7 batrachotoxins (**615–621**) were isolated in minute quantities from the skins of poison arrow frogs (*Phyllobates aurotaenia*) as well as from the skins and feathers of New Guinea birds (genus *Pitohui* and *Iflita*) (Table 13). They exhibit novel structural features, including a steroid-based pentacyclic core skeleton, an intramolecular 3-hemiketal, and a seven-membered oxazapane ring (Fig. 15) [301].

**Table 13:** Structures and sources of batrachotoxins **615–621**

No	Compounds	Substitution groups and others	Sources	References
**615**	Batrachotoxinin A	R = H	*Phyllobates aurotaenia*	[302]
**616**	Pseudobatrachotoxin	R = X_1_	*P. aurotaenia*	[302]
**617**	Batrachotoxin	R = X_2_	*P. aurotaenia*	[302]
**618**	Homobatrachotoxin	R = X_3_	*P. aurotaenia; Pitohui dichrous*	[302, 303]
**619**	Batrachotoxinin A-20*R*-*cis*-crotonate	R = COCHCHCH_3_	*Ifrita kowaldi*	[304]
**620**	Batrachotoxinin A-20*R*-3′-hydroxypentanoate	R = COCH_2_CH(OH)CH_2_CH_3_	*I. kowaldi*	[304]
**621**	Batrachotoxinin A-20*R*-acetate	R = Ac	*I. kowaldi*	[304]

The structure of pseudobatrachotoxin (**616**) is the 20*α*-p-bromobenzoate of batrachotoxinin A (**615**) [302]. Batrachotoxin (**617**) is the 20*α* ester of **615** with 2,4-dimethylpyrrole-3-carboxylic acid [302], while homobatrachotoxin (**618**) is the 20*α* ester of **615** with 2-ethyl-4-methylpyrrole-3-carboxylic acid [303]. These structures were confirmed by partial synthesis of batrachotoxin selective acylation.

##### Plakinamines

**Fig. 16:**
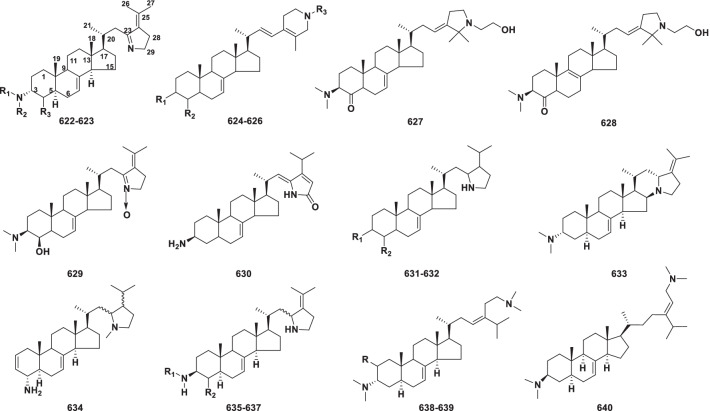
Structures of plakinamines **622–640**

Considerable research effort has been focused on the discovery of new bioactive natural products from marine animals. A number of new steroidal alkaloids have been isolated in the process, mostly from marine invertebrates. A marine sponge of the genus *Plakina* and *Corticium* sp. yielded nineteen new steroidal alkaloids (**622–640**), namely, plakinamine (Table 14). Plakinamines have modified ergostane-type steroidal cores, as they possess nitrogen substitution at C-3 in the A ring and linear or cyclized nitrogenous side chains (Fig. 16) [305].

**Table 14:** Structures and sources of plakinamines **622–640**

No	Compounds	Substitution groups and others	Sources	References
**622**	Plakinamine A	R_1_ = R_2_ = R_3_ = H	*Plakina* sp.	[308]
**623**	Plakinamine F	R_1_ = R_2_ = CH_3_; R_3_ = O	*Corticium* sp.	[309]
**624**	Plakinamine B	R_1_ = *α*-NHCH_3_; R_2_ = H; R_3_ = CH_3_	*Plakina* sp.	[308]
**625**	Plakinamine H	R_1_ = *β*-N(CH_3_)_2_; R_2_ = O; R_3_ = H	*Corticium* sp.	[306]
**626**	4*α*-Hydroxydemethylplakinamine B	R_1_ = *α*-NH_2_; R_2_ = *β*-OH; R_3_ = CH_3_	*Corticium* sp.	[306]
**627**	Plakinamines C		*Corticium* sp.	[310]
**628**	Plakinamines D		*Corticium* sp.	[310]
**629**	Plakinamine E		*Corticium* sp.	[309]
**630**	Plakinamine G		*Corticium* sp.	[306]
**631**	Tetrahydroplakinamine A	R_1_ = *α*-NH_2_; R_2_ = H	*Corticium* sp.	[306]
**632**	Dihydroplakinamine K	R_1_ = *β*-NH_2_; R_2_ = *β*-OAc	*Corticium niger*	[307]
**633**	Plakinamine I		*C. niger*	[307]
**634**	Plakinamine J		*C. niger*	[307]
**635**	Plakinamine K	R_1_ = CH_3_; R_2_ = *β*-OAc	*C. niger*	[307]
**636**	Plakinamine N	R_1_ = R_2_ = H	*C. niger*	[311]
**637**	Plakinamine O	R_1_ = H; R_2_ = *β*-OAc	*C.niger*	[311]
**638**	Plakinamine L	R = H	*Corticium* sp.	[305]
**639**	Plakinamine M	R = *β*-OH	*Corticium* sp.	[312]
**640**	Plakinamine P		*Plakina* sp.	[313]

Plakinamine G (**630**) bearing a rare side chain with an *α*,*β*-unsaturated *γ*-lactam ring was structurally assigned by 2D NMR spectroscopy and accurate mass measurements (HR-EIMS) [306]. Most plakinamines contain a substituted pyrrolidine ring in the steroidal side chain, only in plakinamine I (**633**) the pyrrolidine nitrogen forms an additional fused piperidine ring system [307]. The first natural representative of steroidal alkaloids with a double bond at C-2 and an amine substituent at C-4 was plakinamine J (**634**) [307]. Three new steroidal alkaloids, plakinamine L, M and P (**638–640**), have unprecedented acyclic side chains, while other compounds contain cyclized nitrogenous side chains.

##### Cortistatins

**Fig. 17:**
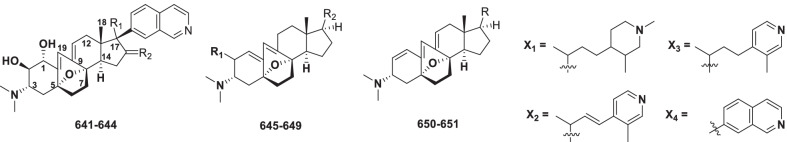
Structures of cortistatins **641–651**

Kobayashi et al. isolated a new family of abeo-9(10-19)-androstane-type steroidal alkaloids with oxabicyclo[3.2.1]octane called cortistatins, from the Indonesian marine sponge *Corticium simplex* (Fig. 17, Table 15) [314]. Up to now, this family has 11 members (**641–651**), with the B and C rings connected through an interesting and characteristic oxo-bridge.

**Table 15:** Structures and sources of cortistatins **641–651**

No	Compounds	Substitution groups and others	Sources	References
**641**	Cortistatin A	R_1_ = H; R_2_ = H; H	*Corticium simplex*	[315]
**642**	Cortistatin B	R_1_ = H; R_2_ = *α*-H; *β*-OH	*C. simplex*	[315]
**643**	Cortistatin C	R_1_ = H; R_2_ = O	*C. simplex*	[315]
**644**	Cortistatin D	R_1_ = OH; R_2_ = O	*C. simplex*	[315]
**645**	Cortistatin E	R_1_ = H; R_2_ = X_1_	*C. simplex*	[316]
**646**	Cortistatin G	R_1_ = H; R_2_ = X_2_	*C. simplex*	[316]
**647**	Cortistatin H	R_1_ = H; R_2_ = X_3_	*C. simplex*	[316]
**648**	Cortistatin K	R_1_ = H; R_2_ = X_4_	*C. simplex*	[317]
**649**	Cortistatin L	R_1_ = *β*-OH; R_2_ = X_4_	*C. simplex*	[317]
**650**	Cortistatin F	R = X_1_	*C. simplex*	[316]
**651**	Cortistatin J	R = X_4_	*C. simplex*	[317]

Cortistatins A–D (**641–644**) and J–L (**651, 648–649**) have a 5-membered E-ring decorated with a unique isoquinoline moiety at C-17.

### Dimeric steroidal alkaloids

#### Cephalostatins

**Fig. 18:**
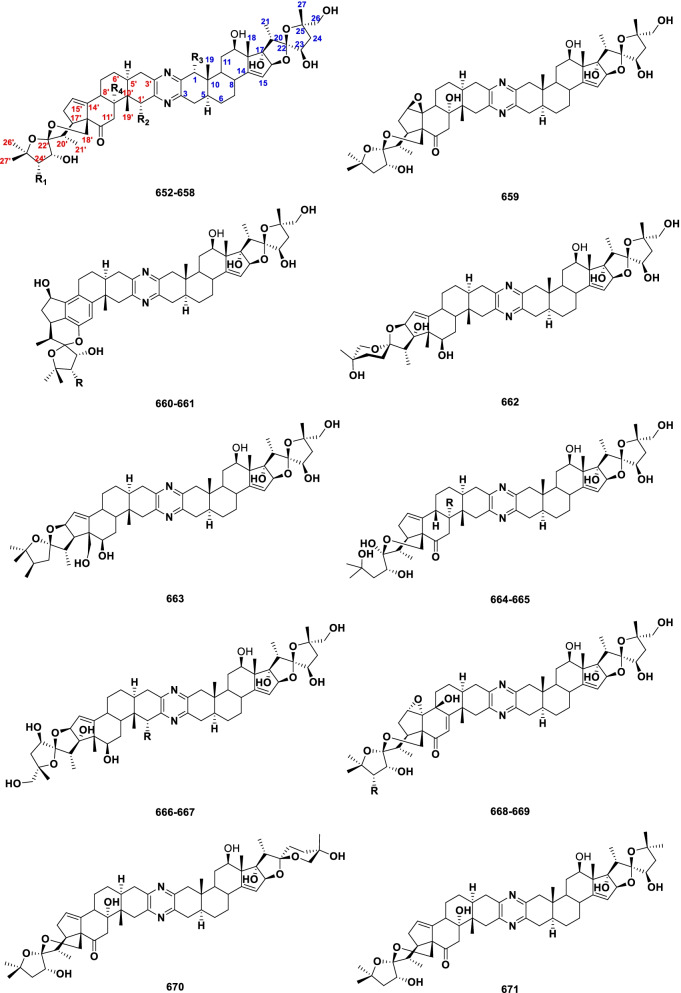
Structures of cephalostatins **652–671**

The 20 cephalostatins (**652–671**) have been isolated only in one marine organism: *Cephalodiscus gilchristi*, a tiny marine worm predominantly found in shallow and temperate waters (Table 16). The structure of cephalostatins, characterized by an adissymmetric bis-steroidal pyrazine framework, consisting of 13 rings is quite unusual (Fig. 18) [20].

**Table 16:** Structures and sources of cephalostatins **652–671**

No	Compounds	Substitution groups and others	Sources	References
**652**	Cephalostatin 1	R_1_ = R_2_ = R_3_ = R_4_ = H	*Cephalodiscus gilchristi*	[322]
**653**	Cephalostatin 2	R_1_ = R_2_ = R_3_ = H; R_4_ = OH	*C. gilchristi*	[323]
**654**	Cephalostatin 3	R_1_ = CH_3_; R_2_ = R_3_ = H; R_4_ = OH	*C. gilchristi*	[323]
**655**	Cephalostatin 10	R_1_ = R_2_ = H; R_3_ = OCH_3_; R_4_ = OH	*C. gilchristi*	[318]
**656**	Cephalostatin 11	R_1_ = R_3_ = H; R_2_ = OCH_3_; R_4_ = OH	*C. gilchristi*	[318]
**657**	Cephalostatin 18	R_1_ = R_2_ = R_4_ = H; R_3_ = OCH_3_	*C. gilchristi*	[324]
**658**	Cephalostatin 19	R_1_ = R_3_ = R_4_ = H; R_2_ = OCH_3_	*C. gilchristi*	[324]
**659**	Cephalostatin 4		*C. gilchristi*	[323]
**660**	Cephalostatin 5	R = CH_3_	*C. gilchristi*	[320]
**661**	Cephalostatin 6	R = H	*C. gilchristi*	[320]
**662**	Cephalostatin 7		*C. gilchristi*	[325]
**663**	Cephalostatin 8		*C. gilchristi*	[325]
**664**	Cephalostatin 9	R = H	*C. gilchristi*	[325]
**665**	Cephalostatin 20	R = OH	*C. gilchristi*	[326]
**666**	Cephalostatin 12	R = H	*C. gilchristi*	[319]
**667**	Cephalostatin 13	R = OH	*C. gilchristi*	[319]
**668**	Cephalostatin 14	R = H	*C. gilchristi*	[327]
**669**	Cephalostatin 15	R = CH_3_	*C. gilchristi*	[327]
**670**	Cephalostatin 16		*C. gilchristi*	[321]
**671**	Cephalostatin 17		*C. gilchristi*	[321]

The atypical C-22′ spiroketals involving C-18′ in most cephalostatins 1–4 (**652−654**, **659**), 9–11 (**664**, **655−656**), 14–19 (**668−671**, **657−658**) and C-12′ in cephalostatins 5 (**660)** and cephalostatins 6 (**661)** are also noteworthy. Cephalostatins 10 (**655**), 11 (**656**) [318], and 13 (**667**) [319] with an oxygen substituent (OMe or OH) at the 1*α*- or 1′*α*-positions, close to the central pyrazine ring, are rare in this type alkaloids. Both cephalostatins 5 (**660**) and 6 (**661**) contain an aromatic C′ ring that is rather unusual in naturally occurring steroids [320]. The only symmetric cephalostatin 12 (**666**) containing two identical steroid units is unique [319]. Cephalostatin 16 (**670**), the only compound contains the [4.5] spiroketal system with the unusual 22*S* configuration (not yet confirmed by synthesis) in the right side steroid unit, whereas other cephalostatins have a common [4.4] spiroketal system in the right steroidal unit [321].

#### Ritterazines

**Fig. 19:**
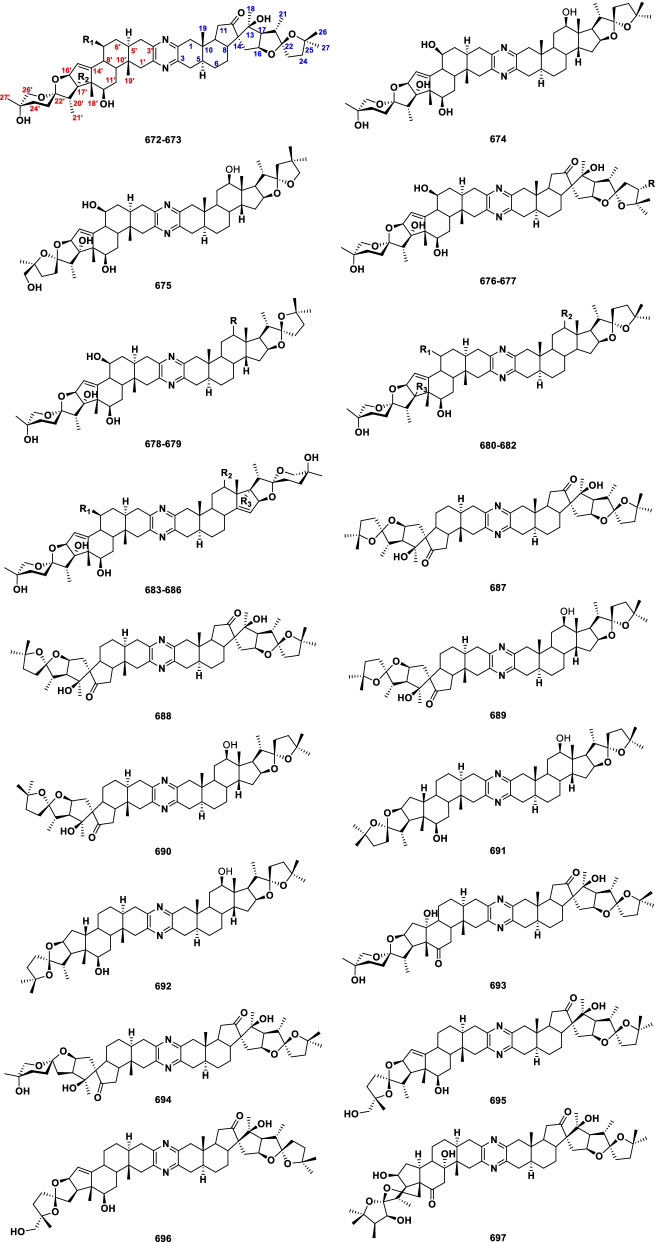
Structures of ritterazines **672–697**

The ritterazine class of steroidal alkaloids comprises 26 compounds (**672–697**), all of which were found in the lipophilic extract of the tunicate *Ritterella tokioka* collected off the Izu Peninsula by Fusetani and colleagues (Table 17). They are spiroketal-containing steroidal heterodimers (Fig. 19). Ritterazines and cephalostatins share common structural features, in which two highly oxygenated hexacyclic steroidal units are fused via a pyrazine ring at C-2 and C-3 and both side chains of the steroidal units form either [4.4] or [4.5] spiroketals [20].

**Table 17:** Structures and sources of ritterazines **672–697**

No	Compounds	Substitution groups and others	Sources	References
**672**	Ritterazine A	R_1_ = R_2_ = OH	*Ritterella tokioka*	[332]
**673**	Ritterazine T	R_1_ = R_2_ = H	*R. tokioka*	[333]
**674**	Ritterazine B		*R. tokioka*	[328, 329]
**675**	Ritterazine C		*R. tokioka*	[328]
**676**	Ritterazine D	R = H	*R. tokioka*	[330]
**677**	Ritterazine E	R = CH_3_	*R. tokioka*	[330]
**678**	Ritterazine F	R = *β*-OH	*R. tokioka*	[330]
**679**	Ritterazine H	R = O	*R. tokioka*	[330]
**680**	Ritterazine G	R_1_ = R_2_ = *β*-OH; R_3_ = *α*-OH; △^14,15^	*R. tokioka*	[330]
**681**	Ritterazine I	R_1_ = *β*-OH; R_2_ = O; R_3_ = *α*-OH	*R. tokioka*	[330]
**682**	Ritterazine Y	R_1_ = R_3_ = H; R_2_ = *β*-OH	*R. tokioka*	[333]
**683**	Ritterazine J	R_1_ = R_3_ = OH; R_2_ = *β*-OH	*R. tokioka*	[330]
**684**	Ritterazine K	R_1_ = H; R_2_ = *β*-OH; R_3_ = OH	*R. tokioka*	[330]
**685**	Ritterazine L	R_1_ = R_3_ = H; R_2_ = *β*-OH	*R. tokioka*	[330]
**686**	Ritterazine M	R_1_ = R_3_ = H; R_2_ = *α*-OH	*R. tokioka*	[330, 331]
**687**	Ritterazine N		*R. tokioka*	[333]
**688**	Ritterazine O		*R. tokioka*	[333]
**689**	Ritterazine P		*R. tokioka*	[333]
**690**	Ritterazine Q		*R. tokioka*	[333]
**691**	Ritterazine R		*R. tokioka*	[333]
**692**	Ritterazine S		*R. tokioka*	[333]
**693**	Ritterazine U		*R. tokioka*	[333]
**694**	Ritterazine V		*R. tokioka*	[333]
**695**	Ritterazine W		*R. tokioka*	[333]
**696**	Ritterazine X		*R. tokioka*	[333]
**697**	Ritterazine Z		*R. tokioka*	[333]

**Fig. 20:**
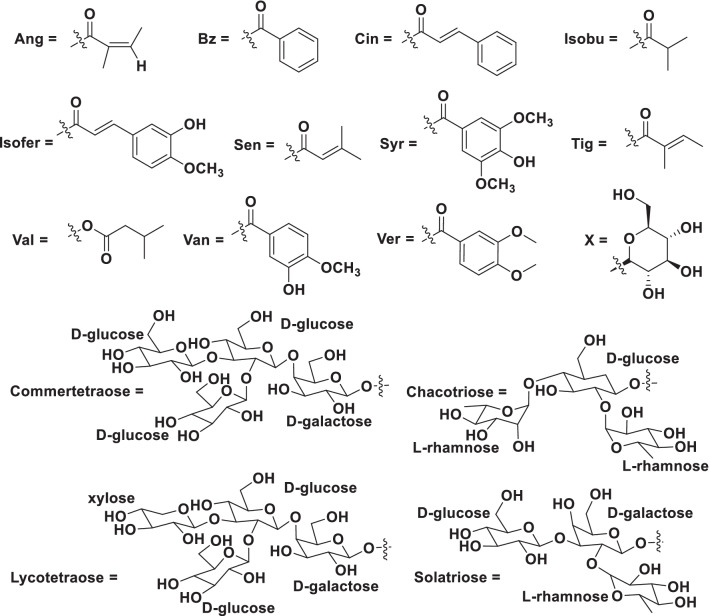
Structural abbreviations used in this review

The hydroxyl groups of cephalostatins at C-12, C-17, C-23, and C-26 are more oxygenated in the right side hemispheres than in ritterazines, which is hydroxylated only at C-12, while the left side hemisphere ritterazines are more oxygenated, with C-7′, C-12′, C-17′ and C-25′ being hydroxylated. All cephalostatins contain *β*-hydroxyl oxygen substituents at the C-12 position, while some ritterazines bear carbonyl groups at this position. In the original paper, the configuration of ritterazine B (**674**) at the spiro carbon atom was mistaken for the same as in cephalostatin 1 in 1995 [328]. However, this has been recently revised by Phillips and Shair, who synthesized the right half of ritterazine B in 2007 [329]. Ritterazines J–M (**683–686**), exhibited the presence of the [4.5] spiroketal system on both sides of the alkaloid molecule, but only one of them, ritterazine K (**684**), was symmetrical [330]. In the original paper ritterazine M (**686**) was erroneously assigned as the *S* configuration at C-22, along with an incorrect configuration at C-12 [330]. A chemical synthesis of this compound by Fuchs et al. allowed correcting the structure [331]. Ritterazines A (**672**), T (**673**), D (**676**), E (**677**), N (**687**), O (**688**), U-X (**693–696**), and Z (**697**) have a unique five-membered C ring on their right side, which is a rearranged steroid nucleus, the same as *Veratrum* alkaloids. Structural abbreviations used in this review are illustrated in Fig. 20.

## Biological activities

### Anticancer effects

Most steroidal alkaloids showed anticancer activity as cytotoxicity with the IC_50_ values listed in Table 18. Among the seven human cancer cell lines SMMC-772, A-549, SK-BR-3, PANC-1, K562, SGC7901 and HL-60, the most sensitive cell line according to sarcovagine D (**116**) was SK-BR-3, which had an IC_50_ value of 2.25 μM. [79]. Holamine (**145**) and funtumine (**154**) exhibited anticancer activity against human colon adenocarcinoma (HT-29) with IC_50_ values of 31.06 and 22.36 μM, respectively. The study demonstrated that **145** and **154** induced cytotoxicity through the induction of apoptosis in HeLa, MCF-7, and HT-29 cancer cells [83]. Then, they induced apoptosis through the elevation of reactive oxygen species (ROS), mitochondrial function modulation, the perturbation of F-actin polymerization, and caspase-3 induction, which were all more prominent in HeLa cells [334].

**Table 18:** Cytotoxic activity of steroidal alkaloids against tumor cell lines

Compounds	Cells	Activity	References
Mokluangin A (**10**)	Small cell lung cancer (NCI-H187)	IC_50_ = 30.6 μM	[41]
Irehline (**36**)	NCI-H187	IC_50_ = 27.7 μM	[41]
3-*Epi-*gitingensine (**38**)	Oral epidermoid carcinoma (KB)	IC_50_ = 21.2 μM	[42]
Paravallarine (**39**)	KB	IC_50_ = 12.8 μM	[42]
Pachysamine E (**57**)	Mouse lymphoid neoplasm (P388)	IC_50_ = 0.46 μg/mL	[54]
	Parental and the Adriamycin (doxorubicin)-resistant subline of mouse leukemia (P388/ADM)	IC_50_ = 0.45 μg/mL	[54]
Hookerianine A (**61**)	Colon cancer (SW480)	IC_50_ = 10.97 ± 1.36 μM	[56]
	Human prostate cancer (PC3)	IC_50_ = 32.97 ± 3.78 μM	[56]
	Breast adenocarcinoma (MCF-7)	IC_50_ = 37.70 ± 0.99 μM	[56]
	Human myelogenous leukemia (K562)	IC_50_ = 11.86 ± 0.82 μM	[56]
Vaganine A (**82**)	Breast cancer (MB-MDA-231)	IC_50_ = 0.18 μM	[82]
Epipachysamine D (**87**)	Human myeloid leukemia (HL-60)	IC_50_ = 2.96 μM; IC_50_ = 2.87 μM	[75, 79]
	Breast adenocarcinoma (MCF-7)	IC_50_ = 28.92 ± 1.22 μM	[56]
Epipachysamine E (**91**)	Human melanoma (B16)	IC_50_ = 2.5 μg/mL	[54]
	Shionogi carcinoma (SC115)	IC_50_ = 3.4 μg/mL	[54]
	Mouse lymphoid neoplasm (P388)	IC_50_ = 0.56 μg/mL	[54]
	Parental and the adriamycin (doxorubicin)-resistant subline of mouse leukemia (P388/ADM)	IC_50_ = 0.66 μg/mL	[54]
Sarcovagine D (**116**)	Hepatocellular carcinoma (SMMC-7721)	IC_50_ = 16.69 μM	[75]
	Lung cancer (A-549)	IC_50_ = 11.17 μM	[75]
	Breast cancer (SK-BR-3)	IC_50_ = 4.17 μM; IC_50_ = 2.25 μM	[75, 79]
	Pancreatic cancer (PANC-1)	IC_50_ = 10.76 μM; IC_50_ = 2.70 μM	[75, 79]
	Human myeloid leukemia (K562)	IC_50_ = 3.53 μM	[79]
	Gastric carcinoma (SGC7901)	IC_50_ = 4.87 μM	[79]
Sarsaligenine A (**128**)	Human myeloid leukemia (HL-60)	IC_50_ = 2.87 μM	[79]
	Human myeloid leukemia (K562)	IC_50_ = 8.48 μM	[79]
	Gastric carcinoma (SGC7901)	IC_50_ = 29.94 μM	[79]
	Breast cancer (SK-BR-3)	IC_50_ = 10.14 μM	[79]
	Pancreatic cancer (PANC-1)	IC_50_ = 12.34 μM	[79]
Sarsaligenine B (**129**)	Human myeloid leukemia (HL-60)	IC_50_ = 3.61 μM	[79]
	Human myeloid leukemia (K562)	IC_50_ = 17.10 μM	[79]
	Gastric carcinoma (SGC7901)	IC_50_ = 21.53 μM	[79]
	Breast cancer (SK-BR-3)	IC_50_ = 17.89 μM	[79]
	Pancreatic cancer (PANC-1)	IC_50_ = 32.84 μM	[79]
Holamine (**145**)	Human myeloid leukemia (HL-60)	IC_50_ = 24.22 μM	[75]
	Human colon adenocarcinoma (HT-29)	IC_50_ = 31.06 μM	[83]
	Human cervical cancer (HeLa)	IC_50_ = 51.42 μM	[83]
	Human breast adenocarcinoma (MCF-7)	IC_50_ = 42.82 μM	[83]
	Non-cancerous human fibroblast (KMST-6)	IC_50_ = 102.95 μM	[83]
Pachysanonin (**149**)	Lewis lung carcinoma (LLC)	IC_50_ = 2.0 ± 0.3 μg/mL	[83]
Funtumine (**154**)	Human colon adenocarcinoma (HT-29)	IC_50_ = 22.36 μM	[83]
	Human cervical cancer (HeLa)	IC_50_ = 46.17 μM	[83]
	Human breast adenocarcinoma (MCF-7)	IC_50_ = 52.69 μM	[83]
	Non-cancerous human fibroblast (KMST-6)	IC_50_ = 85.45 μM	[83]
Pachystermine A (**157**)	Human melanoma (B16)	IC_50_ = 6.3 μg/mL	[54]
	Breast cancer (MB-MDA-231)	IC_50_ = 0.32 μM	[82]
Terminamine A (**159**)	MB-MDA-231	IC_50_ = 0.18 μM	[82]
Terminamine B (**160**)	MB-MDA-231	IC_50_ = 0.20 μM	[82]
Terminamine C (**161**)	MB-MDA-231	IC_50_ = 0.08 μM	[82]
Terminamine D (**163**)	MB-MDA-231	IC_50_ = 0.20 μM	[82]
Terminamine E (**164**)	MB-MDA-231	IC_50_ = 0.07 μM	[82]
Hookerianine B (**171**)	Colon cancer (SW480)	IC_50_ = 5.97 ± 0.13 μM	[56]
	Human hepatocarcinoma (SMMC-7721)	IC_50_ = 16.19 ± 0.56 μM	[56]
	Human prostate cancer (PC3)	IC_50_ = 11.57 ± 0.86 μM	[56]
	Breast adenocarcinoma (MCF-7)	IC_50_ = 19.44 ± 1.70 μM	[56]
	Human myelogenous leukemia (K562)	IC_50_ = 7.95 ± 0.02 μM	[56]
Veratramine (**392**)	Lung cancer (A549)	IC_50_ = 8.9 μmol/L	[185]
	Pancreatic cancer (PANC-1)	IC_50_ = 14.5 μmol/L	[185]
	Hh-dependent (SW1990)	IC_50_ = 26.1 μmol/L	[185]
	Hh-dependent (NCI-H249)	IC_50_ = 8.5 μmol/L	[185]
	Human glioma (SF188)	IC_50_ = 97.8 μmol/L	[198]
Germine (**358**)	Hh-dependent (SW1990)	IC_50_ = 47.2 μmol/L	[185]
	Hh-dependent (NCI-H249)	IC_50_ = 24.1 μmol/L	[185]
Cyclopamine (**432**)	Lung cancer (A549)	IC_50_ = 14.4 μmol/L	[185]
	pancreatic cancer (PANC-1)	IC_50_ = 29.3 μmol/L	[185]
	Hh-dependent (SW1990)	IC_50_ = 48.6 μmol/L	[185]
	Hh-dependent (NCI-H249)	IC_50_ = 4.4 μmol/L	[185]
	Human pancreatic adenocarcinoma (HPAF-2)	IC_50_ = 8.79 ± 0.94 μM	[335]
	Human pancreatic adenocarcinoma cell line Panc 10.05	IC_50_ = 11.33 ± 0.41 μM	[335]
	Human pancreatic adenocarcinoma cell line Panc 8.13	IC_50_ = 14.49 ± 0.85 μM	[335]
	Human pancreatic adenocarcinoma cell line Panc 2.03	IC_50_ = 16.57 ± 0.27 μM	[335]
	Human pancreatic adenocarcinoma cell line AsPC-1	IC_50_ = 16.74 ± 1.30 μM	[335]
	Human pancreatic adenocarcinoma cell line CFPAC-1	IC_50_ = 19.59 ± 0.32 μM	[335]
	Human pancreatic adenocarcinoma cell line BxPC-3	IC_50_ = 36.17 ± 0.31 μM	[335]
	Human pancreatic adenocarcinoma cell line S2013	IC_50_ = 45.09 ± 1.27 μM	[335]
*α*-Tomatine (**459**)	Breast cancer (MDA-MB-231)	IC_50_ = 26.4 ± 3.6 μg/mL	[348]
	Gastric adenocarcinoma (KATO-III)	IC_50_ = 16.4 ± 10.0 μg/mL	[348]
	Prostate cancer (PC3)	IC_50_ = 3.0 ± 0.3 μg/mL	[348]
*α*-Solamargine (**500**)	Human adenocarcinoma (H441)	IC_50_ = 3.0 μM	[345]
	Squamous cell lung carcinoma (H520)	IC_50_ = 6.7 μM	[345]
	Large cell lung cancer (H661)	IC_50_ = 7.2 μM	[345]
	Small cell lung cancer (H69)	IC_50_ = 5.8 μM	[345]
	Cervical carcinoma (HeLa)	IC_50_ = 6.0 μM	[346]
	Lung cancer (A549)	IC_50_ = 8.0 μM	[346]
	Breast adenocarcinoma (MCF-7)	IC_50_ = 2.1 μM	[346]
	Human myelogenous leukemia (K562)	IC_50_ = 5.2 μM	[346]
	Colon cancer cell line (HCT116)	IC_50_ = 3.8 μM	[346]
	Human primary glioblastoma (U87)	IC_50_ = 3.2 μM	[346]
	Liver cancer (HepG2)	IC_50_ = 2.5 μM	[346]
*α*-Solanine (**528**)	HepG_2_	IC_50_ = 14.47 μg/mL	[255]
Plakinamine H (**625**)	Rat glioma (C6)	IC_50_ = 9.0 μg/mL	[306]
Plakinamine G (**630**)	C6	IC_50_ = 6.8 μg/Ml	[306]
Tetrahydroplakinamine A (**631**)	C6	IC_50_ = 1.4 μg/mL	[306]
Dihydroplakinamine K (**632**)	Human colon tumor (HCT-116)	IC_50_ = 1.4 μM	[307]
Plakinamine I (**633**)	HCT-116	IC_50_ = 5.2 μM	[307]
Plakinamine J (**634**)	HCT-116	IC_50_ = 10.6 μM	[307]
Plakinamine K (**635**)	HCT-116	IC_50_ = 6.1 μM	[307]
Cephalostatin 1 (**652**)	Pancreas adenocarcinoma (BXPC-3)	GI_50_ = 0.044 nM	[326]
	Breast adenocarcinoma (MCF-7)	GI_50_ = 0.099 nM	[326]
	Glioblastoma (SF-268)	GI_50_ = 1.60 nM	[326]
	Human lung large cell carcinoma (NCI-H460)	GI_50_ = 0.044 nM	[326]
	Colon carcinoma (KM20L2)	GI_50_ = 0.066 nM	[326]
	Human prostate adenocarcinoma (DU-145)	GI_50_ = 0.11 nM	[326]
Cephalostatin 2 (**653**)	Pancreas adenocarcinoma (BXPC-3)	GI_50_ = 0.022 nM	[326]
	Breast adenocarcinoma (MCF-7)	GI_50_ = 0.022 nM	[326]
	Glioblastoma cells (SF-268)	GI_50_ = 0.12 nM	[326]
	Human lung large cell carcinoma (NCI-H460)	GI_50_ = 0.0056 nM	[326]
	Colon carcinoma (KM20L2)	GI_50_ = 0.0060 nM	[326]
	Human prostate adenocarcinoma (DU-145)	GI_50_ = 0.11 nM	[326]
Cephalostatin 9 (**664**)	Pancreas adenocarcinoma (BXPC-3)	GI_50_ = 14 nM	[326]
	Breast adenocarcinoma (MCF-7)	GI_50_ = 110 nM	[326]
	Glioblastoma (SF-268)	GI_50_ = 150 nM	[326]
	Human lung large cell carcinoma (NCI-H460)	GI_50_ = 39 nM	[326]
	Colon carcinoma (KM20L2)	GI_50_ = 58 nM	[326]
Cephalostatin 20 (**665**)	Pancreas adenocarcinoma (BXPC-3)	GI_50_ = 16 nM	[326]
	Breast adenocarcinoma (MCF-7)	GI_50_ = 22 nM	[326]
	Glioblastoma (SF-268)	GI_50_ = 36 nM	[326]
	Human lung large cell carcinoma (NCI-H460)	GI_50_ = 6.00 nM	[326]
	Colon carcinoma (KM20L2)	GI_50_ = 7.20 nM	[326]
	Human prostate adenocarcinoma (DU-145)	GI_50_ = 210 nM	[326]
Ritterazine A (**672**)	Murine leukemia (P388)	IC_50_ = 0.0035 μg/mL	[333]
Ritterazine T (**673**)	P388	IC_50_ = 0.46 μg/mL	[333]
Ritterazine B (**674**)	P388	IC_50_ = 0.00015 μg/mL	[333]
Ritterazine C (**675**)	P388	IC_50_ = 0.092 μg/mL	[333]
Ritterazine D (**676**)	P388	IC_50_ = 0.016 μg/mL	[333]
Ritterazine E (**677**)	P388	IC_50_ = 0.0035 μg/mL	[333]
Ritterazine F (**678**)	P388	IC_50_ = 0.00073 μg/mL	[333]
Ritterazine H (**679**)	P388	IC_50_ = 0.016 μg/mL	[333]
Ritterazine G (**680**)	P388	IC_50_ = 0.00073 μg/mL	[333]
Ritterazine I (**681**)	P388	IC_50_ = 0.014 μg/mL	[333]
Ritterazine Y (**682**)	P388	IC_50_ = 0.0035 μg/mL	[333]
Ritterazine J (**683**)	P388	IC_50_ = 0.013 μg/mL	[333]
Ritterazine K (**684**)	P388	IC_50_ = 0.0095 μg/mL	[333]
Ritterazine L (**685**)	P388	IC_50_ = 0.010 μg/mL	[333]
Ritterazine M (**686**)	P388	IC_50_ = 0.015 μg/mL	[333]
Ritterazine N (**687**)	P388	IC_50_ = 0.46 μg/mL	[333]
Ritterazine O (**688**)	P388	IC_50_ = 2.1 μg/mL	[333]
Ritterazine P (**689**)	P388	IC_50_ = 0.71 μg/mL	[333]
Ritterazine Q (**690**)	P388	IC_50_ = 0.57 μg/mL	[333]
Ritterazine R (**691**)	P388	IC_50_ = 2.1 μg/mL	[333]
Ritterazine S (**692**)	P388	IC_50_ = 0.46 μg/mL	[333]
Ritterazine U (**693**)	P388	IC_50_ = 2.1 μg/mL	[333]
Ritterazine V (**694**)	P388	IC_50_ = 2.1 μg/mL	[333]
Ritterazine W (**695**)	P388	IC_50_ = 3.2 μg/mL	[333]
Ritterazine X (**696**)	P388	IC_50_ = 3.0 μg/mL	[333]
Ritterazine Z (**697**)	P388	IC_50_ = 2.0 μg/mL	[333]

Cyclopamine (**432**), a Hedgehog (Hh) signaling pathway antagonist, was first identified as a potent teratogen in animals. Among the nine human pancreatic cell lines examined, the IC_50_ values of cyclopamine ranged from 8.79 to more than 30 µM [335]. In addition, **432** also showed prominent anticancer effects, including small-cell lung cancer (SCLC) [336], oral squamous cell carcinoma (OSCC) [337], breast cancer [338], pancreatic cancer [339], hepatocellular carcinoma (HCC) [340] and human erythroleukemia cells [341]. Furthermore, **432** induced apoptosis in HCC cells through inhibition of the Sonic Hh signaling pathway by downregulating Bcl-2 [340]. In addition, **432** could induce apoptosis and upregulate cyclooxygenase-2 (COX-2) expression which plays a crucial role in the proliferation and differentiation of leukemia cells [341].

Tomatidine (**458**) and solasodine (**513**), important alkaloids found in a large number of *Solanum* species, exerted cytotoxic activity against HBL-100 cells [342]. They had a weak inhibitory effect on MCF-7, HT-29 and HeLa cells by blocking the cell cycle in the G_0_/G_1_ phase [343]. *α*-Solamargine (**500**) and *α*-solasonine (**501**), the two glycosides of **513**, differed only in their carbohydrate moieties, which are used in the treatment of keratoses, basal cell carcinomas, and squamous cell carcinomas [344]. Moreover, **500** was significantly cytotoxic to the human tumor cell lines H441, H520, H661, H69, HeLa, A549, MCF-7, K562, HCT116, U87 and HepG2 with IC_50_ values from 2.1 to 8.0 μM [345, 346]. The cellular and molecular mechanism of **500** anti-human breast cancer cells HBL-100, ZR-75-1 and SK-BR-3 were investigated, and it was concluded that this compound could activate apoptotic proteins and inhibite anti-apoptotic, so it has great potential as an anti-human breast cancer candidate drug [347]. The target of *α*-solanine (**528**) inducing apoptosis in HepG_2_ cells seemed to be mediated by the inhibition of the expression of Bcl-2 protein [255].

Cephalostatin 1–20 (**652–671**) were significantly cytotoxic to the human tumor cell lines BXPC-3, MCF-7, SF-268, NCI-H460, KM20L2 and DU-145. Of these compounds, cephalostatin 2 (**653**) was the most active compound, with GI_50_ (growth inhibition of 50%) values in the range of 0.0056–0.11 nM. Importantly, compared with the cephalostatins 9 (**664**) and 20 (**665**), the inhibitory effects of **653** and cephalostatin 1 (**652**) were significantly increased by 100–1000 times. From this evidence, it was clear that the spirostanol structure must be intact and was the critical center for antineoplastic activities. The opening of the left-side spiro-ring significantly reduced the inhibition of these carcinoma cells. A significant contribution of the presence of a hydroxy group at C-8′ to antineoplastic potency was evident by comparing the activity of cephalostatins 2 (**653**) and 1 (**652**), which was further supported by the cancer growth inhibitory activity of cephalostatins 20 (**665**) and 9 (**664**). Compounds **653** and **665**, in which the hydroxy substitution at C-8′, had considerably increased activity compared with compounds **652** and **664**, respectively [326].

Ritterazines A–Z (**672–697**) were all significantly cytotoxic to the human tumor cell lines of P388 murine leukemia cells. Of these compounds, ritterazine B (**674**) was the most active with an IC_50_ value of 0.00015 μg/mL. The presence of both the terminal 5/6 spiroketal and the hydroxyl groups was found to be especially important for pronounced inhibition of P388 cells. Ritterazines B (**674**) and F (**678**), which have terminal 5/6 spiroketal, showed high cytotoxicity against P388 cells, whereas ritterazine C, possessing 5/5 spiroketal structure, showed a lower significant level of cytotoxicity [333].

### Anticholinergic effects

Some pregnane and cyclopregnane type alkaloids are distributed in many genera of Apocynaceae and display significant anticholinergic activity. Cholinesterase (ChE), divided into two enzymes acetylcholinesterase (AChE) and butyrylcholinestarase (BChE), have been identified as potential targets in the treatment of AD, myasthenia gravis and glaucoma. The IC_50_ values of AChE and BchE inhibited by most steroidal alkaloids are listed in Table 19.

**Table 19:** Cholinesterase-inhibiting activities of steroidal alkaloids

Compounds	IC_50_/μM		References
	AChE	BChE	
Salonine B (**47**)	n.a	4.5	[50]
2-Hydroxysalignamine (**49**)	82.5	20.9	[51]
*N*-[Formyl(methyl)amino]salonine B (**50**)	48.6	10.5	[51]
Sarsalignone (**64**)	7	2.2	[68]
Sarsaligenone (**65**)	5.8	4.3	[70]
Alkaloid C (**71**)	48.6	10.5	[50]
Salignarine F (**72**)	30.2	1.9	[51]
Saracosine (**73**)	20	3.8	[51]
Sarcodinine (**74**)	40	12.5	[51]
Sarcovagine C (**80**)	8	0.3	[74]
Vaganine A (**82**)	8.6	2.3	[70]
Sarcorine (**83**)	70	10.3	[70]
Saligcinnamide (**85**)	20	4.8	[70]
*N*_a_-Methyl epipachysamine D (**86**)	10.1	3.2	[71]
Epipachysamine D (**87**)	28.9	2.8	[51]
Salignenamide A (**88**)	50.6	4.6	[70]
Iso-*N*-Formylchonemorphine (**90**)	6.3	4.07	[51]
Saligenamide C (**93**)	61.3	38.3	[70]
Saligenamide F (**94**)	6.3	4.1	[70]
2*β*-Hydroxyepipachysamine D (**95**)	78.2	28.9	[70]
Axillarine C (**96**)	227.9	18	[70]
Axillarine F (**97**)	182.4	18.2	[70]
Salonine A (**98**)	33.4	32.7	[50]
Dictyophlebine (**99**)	6.2	3.6	[51]
Hookerianamine A (**100**)	18.9	0.9	[71]
Isosarcodine (**101**)	10.3	1.9	[72]
Hookerianamide B (**102**)	26.4	0.7	[71]
Hookerianamide C (**103**)	23.2	0.6	[71]
Hookerianamide E (**105**)	15.9	6	[73]
Hookerianamide G (**106**)	11.4	1.5	[73]
Hookerianamide I (**107**)	34.1	0.3	[74]
Sarcovagine D (**116**)	2.2	2.3	[71]
Sarcovagenine C (**117**)	1.5	0.7	[71]
Axillaridine A (**118**)	5.21	2.5	[70]
2,3-Dehydrosarsalignone (**119**)	7	32.3	[61]
Phulchowkiamide A (**121**)	0.5	0.4	[71]
Hookerianamide F (**122**)	1.6	7.2	[73]
Hookerianamide H (**123**)	2.9	1.9	[74]
(−)-Vaganine D (**133**)	46.9	10	[80]
5,6-Dihydrosarconidine (**135**)	20.3	1.9	[51]
16-Dehydrosarcorine (**136**)	12.5	3.9	[61]
Hookerianamide A (**137**)	82.7	200	[71]
Saligenamide D (**140**)	185.2	23.7	[70]
2-Hydroxysalignarine E (**141**)	16	6.9	[51]
Salonine C (**142**)	7.8	32.3	[51]
Buxasamarine (**196**)	25.4	0.7	[100]
Cycloprotobuxine C (**201**)	38.8	2.7	[100]
Cyclovirobuxeine A (**202**)	105.7	2	[100]
(+)-Benzoylbuxidienine (**238**)	35	No	[111]
Hyrcatrienine (**257**)	No	1.7	[93]
Hyrcanone (**273**)	145	20	[93]
(+)-*O*^6^-Buxafurandiene (**283**)	17	No	[111]
(+)-7-Deoxy-*O*^6^-buxafurandiene (**284**)	13	No	[111]
Impericine (**312**)	67.97 ± 2.46	1.607	[139]
Forticine (**313**)	> 500	100.5 ± 0.445	[139]
Delavine (**325**)	105.5 ± 1.45	1.706 ± 0.11	[139]
Persicanidine A (**345**)	352.2 ± 4.03	4.245 ± 0.079	[139]

*n.t.* not tested, *n.a.* not active

Phulchowkiamide A (**121**), containing a carbonyl group at C-4 along with the tigloylamino moiety at position C-3, was found to be the most potent inhibitor of AChE and BChE among these alkaloids with IC_50_ values of 0.5 and 0.4 μM, respectively [71]. Similarly, compounds such as sarsalignone (**64**) [68], sarsaligenone (**65**) [70], sarcovagine D (**116**), sarcovagenine C (**117**) [71], and hookerianamide F (**122**) [73], which have in common with **121**, displayed higher inhibitory activity than other compounds. In general, the *α*,*β*-unsaturated carbonyl group and tigloylamino moiety might be considered to be important factors to increase the activity.

From the list, we found that some alkaloids, including axillarine C (**96**), hookerianamide B (**102**), hookerianamide C (**103**), saligenamide D (**140**), cyclovirobuxeine A (**202**), hyrcanone (**273**), impericine (**312**), delavine (**325**), and persicanidine A (**345**), appeared to be more selective inhibitors of BChE. The presence of a C-2*β* hydroxy group, as in 2-hydroxysalignamine (**49**), saligenamide C (**93**), axillarine C (**96**), axillarine F (**97**), salonine A (**98**), and hookerianamide A (**137**) caused a negative effect on the inhibitory activity towards both AChE and BChE. In general, pregnane alkaloids were more selective than cyclopregnane alkaloids towards AChE and BChE. This might be due to the effect of the C-4 methyl groups and the cyclopropane ring in cyclopregnane alkaloids that decreased the activity.

### Antimicrobial effects

Steroidal alkaloids are considered a part of plant chemical defenses against various pathogens, namely, fungi, bacteria, and viruses. Epipachysamine-*E*-5-ene-4-one (**66**) and iso-*N*-formylchonemorphine (**90**) showed strong antibacterial activity against a wide range of pathogenic bacteria (*Bacillus cereus*, *Klebsiella pneumoniae*, *Staphylococcus aureus* and *Pseudom aeruginosa*) with minimum inhibitory concentrations (MICs) of 0.0312–0.2500 (mg/mL), compared with the widely used antibiotics amoxicillin and ampicillin (0.0625–0.2500 mg/mL) [59]. The five pregnane alkaloids sarcovagine C (**80**), hookerianamide I (**107**), chonemorphine (**108**), *N*-methypachysamine A (**109**) and hookerianamide H (**123**), were all active in antibacterial properties against *Bacillus subtilis* with MIC values of lower than 20 μg/mL, and most of them displayed moderate to good antibacterial activities against *Micrococcus luteus*, *Streptococcus faecalis*, and *Pseudomonas pallida* [349]. As saligcinnamide (**85**), *N*_a_-methyl epipachysamine D (**86**) and epipachysamine D (**87**) had the same skeleton, and possessed potent antibacterial activity against seven human pathogenic bacteria with inhibition zones ranging from 6 to 12 mm [67].

(+)-16*α*,31-Diacetylbuxadine (**278**) exhibited significant antibacterial activity with zones of inhibition (ZI) of 14–19 mm against *K. pneumoniae* and *Salmonella typhi* and moderate to weak activity (ZI = 4–12 mm) against other seven human pathogenic bacterias [92]. Neoverataline A (**379**), neoverataline B (**380**), stenophylline B (**564**), veramiline-3-*O*-*β*-d-glucopyranoside (**566**) and jervine (**427**) were tested for antifungal properties against the phytopathogens *Phytophthora capisis* and *Rhizoctonia cerealis,* among which **380**, **564** and **566** displayed strong activity against *P. capisis* with MICs at 120, 80 and 80 μg/mL, respectively. The MIC of triadimefon, a positive control, against *P. capisis* was 80 μg/mL [125].

Tomatidine (**458**) potentiated the action of several aminoglycoside antibiotics (gentamicin, kanamycin, tobramycin, amikacin and streptomycin) against *S. aureus*, and the synergy between **458** and aminoglycosides could help reduce the incidence of resistance. Furthermore, **458** affected the haemolytic ability of *S. aureus* and repressed several agr-regulated virulence factors [350]. *α*-Chaconine (**527**), *α*-solanine (**528**), *α*-solamargine (**500**), *α*-solasonine (**501**), and *α*-tomatine (**459**) showed antimalarial activity, among which the most active compound **527** had no additive effect with **528**. When orally administered at 7.5 mg/kg/day for 4 days, **527** suppressed the parasitemia level by 71.38% [351]. Among the four mycobacterial species, *Mycobacterium tuberculosis*, *Mycobacterium avium*, *Mycobacterium intracellulare* and *Mycobacterium simiae*, plakinamine P (**640**) exhibited the strongest antibacterial effect against *M. tuberculosis*, giving a MIC of 1.8 μg/mL [313].

### Anti-inflammatory and analgesic effects

Solasodine (**513**) significantly reduced the inflammatory reaction to carrageenan-induced rat paw oedema from 19.5 to 56.4% [352]. In addition, the antinociceptive activity of **513** was evaluated by a hot plate, formalin, and writhing tests. **513** caused a significant decrease in nociception at a dose of 8 mg/kg in acetic acid-induced mice abdominal constrictions, with a maximum inhibition of 61%, compared to indomethacin (74%). It could also significantly reduce the painful sensation caused by formalin and produce a significant increase in the pain threshold in the hot plate test. Overall, the results suggested that **513** may possess analgesic activity through both central and peripheral mechanisms [353].

The data provided by Chiu et al*.* suggested that tomatidine (**458**) inhibited NF-κB nuclear translocation and c-Jun N-terminal kinase activation, thereby decreasing the expression of COX-2 and inducible cytotoxic nitric oxide (NO) synthase, which might be beneficial for anti-inflammatory therapy. They also found **458** had a better anti-inflammatory effect than solasodine (**513**) in Lipopolysaccharide (LPS)-stimulated RAW 264.7 mouse macrophages [354].

*α*-Chaconine (**527**) and solanidine (**535**) were responsible for the anti-inflammatory effect, which was dependent on reducing the production of interleukin-2 and interleukin-8 induced by canidin A in Jurkat cells, and the induced NO production by LPS stimulated macrophages [355].

### Anti-myocardial ischemia effects

*Buxus microphylla* is often used to treat cardiovascular and cerebrovascular diseases as *a* folk medicine in China. Cyclovirobuxine D (**203**) was the most potent component contributing to the anti-myocardial ischemia effects of the “huangyangning” tablet. This Chinese drug is used to treat cardiovascular and cerebrovascular diseases and has been developed successfully for more than 10 years in China. In the myocardial ischemia model induced by isoprenaline or pituitrin, 1.1 and 2.2 mg/kg cyclovirobuxine D could improve model rat plasma superoxide dismutase (SOD) activation, and reduce the plasma MDA, LDH, and phosphocreatine kinase (CPK) contents of model rats [356]. The main mechanism of **203** in treating acute myocardial ischemia may be attributed to inhibiting blood stasis and thrombosis, enhancing NO release, and opening K_ATP_ channels [357]. In addition, data from a study of rats with congestive heart failure showed significant benefits after oral administration of **203**, indicating that it may be a promising and useful drug in the treatment of cardiac dysfunction [358].

### Anti-giogenesis effects

Cortistatins, novel steroidal alkaloids extracted from *Corticium sponge*, showed highly selective anti-proliferative activity against human umbilical vein endothelial cells (HUVECs), which inhibited the formation of original capillaries, a process known as angiogenesis [314]. Among the eleven cortistatins A–J (**641−651**), cortistatins A (**641**) and J (**651**) showed the most strongest anti-proliferative action against HUVECs with IC_50_ values of 1.8 and 8 nM, which were 3000 and 300**–**1100 times more selective than normal human dermalfibroblast (NHDF) and tumor cell murine neuroblastoma cells (Neuro2A), respectively. [315].

### Others

Among the four conanine-type alkaloids, conessine (**1**), conimin (**9**), mokluangin A (**10**), and irehline (**36**), **36** showed the most effective antimalarial activity (IC_50_ = 1.2 μM) against *Plasmodium falciparum,* comparable to that of the positive control dihydroartemisinine with an IC_50_ of 3.7 nM [41].

The anti-tussive activity of three steroidal alkaloids was also investigated. yibeinone C (**347**), imperialine (**335**), yibeinone B (**402**), and showed an apparent concentration-dependent relaxation of isolated tracheal preparation, amongst **347** and **335** showed significant effects with pA_2_ values of 6.19 and 8.41, and EC_50_ values of 0.65 μmol/L and 4.40 nmol/L, respectively [156].

The five steroidal alkaloids puqienine A (**400**), puqienine B (**401**), puqietinone (**577**), *N*-demethylpuqietinone (**579**), and puqietinonoside (**580**) could significantly prolong the latent period and reduce the number of coughs in ammonia-induced mouse cough models at doses of 5 and 10 mg/kg, confirming their antitussive activity compared to the positive control codeine. The presence of these compounds may be responsible for the traditional use of *Fritillaria puqiensis* in cough remedies [188].

Plakinamines J (**634**), N (**636**), and O (**637**), containing a substituted pyrrolidine ring, showed potent antiproliferative activity against seven human colon carcinoma cell lines with mean GI_50_ values of 11.5, 2.4 and 1.4 μM, respectively, whereas plakinamine I (**633**) with the pyrrolidine nitrogen formed an additional fused piperidine ring system that exhibited relatively weak activity [311].

## Toxicity

Jervine (**427**) and cyclopamine (**432**), veratrum alkaloids isolated from *Veratrum californicum*, had prominent teratogenic activity to produce synophthalmia and related cephalic malformations in sheep, cattle, goats and rabbits [359, 360]. In addition, the presence of C-5, C-6 olefinic linkages in the framework of jervanes was found to be a critical structural factor to enhance teratogenicity induction [361].

In both pregnant and nonpregnant mice, tomatidine (**458**), solasodine (**513**), and solanidine (**535**) induced an increase in liver weight after being fed a diet containing 2.4 mmol/kg of these aglycones for 14 days [362].

In terms of the LC_50_ and EC_50_ after 96 h of exposure, *α*-chaconine (**527**) was teratogenic and more embryotoxic than *α*-solanine (**528**) in frogs. The carbohydrate side chain attached to the 3-OH group of solanidine (**535**), the only difference between these two compounds, appeared to be an important factor in governing teratogenicity [363].

A pathophysiological study showed that isorubijervine (**543**) and rubijervine (**544**) were highly toxic compounds with LD_50_ values of 1.14 and 1.77 mg/kg in mice, respectively. They also exerted the strongest ability to inhibit the sodium channel Na_V_1.5, which plays an essential role in cardiac physiological function [263].

## Summary

Natural steroidal alkaloids with diverse bioactivities and high toxicity keep them one of the highlighted types of natural products. In this review, the structural diversity and biological activities of 697 natural steroidal alkaloids have been summarized and it is likely that many more steroidal alkaloids with novel structures will be discovered, especially rings E and F. Additionally, the high medicinal potential of cyclovirobuxine D, cyclopamine, *α*-solamargine, *α*-solasonine, cephalostatin 1 and many other members of this intriguing family of natural products is far from being exploited. Therefore, future research in this field will further contribute to understanding their full potential in drug development.
